# Structural and functional analysis of mRNA export regulation by the nuclear pore complex

**DOI:** 10.1038/s41467-018-04459-3

**Published:** 2018-06-13

**Authors:** Daniel H. Lin, Ana R. Correia, Sarah W. Cai, Ferdinand M. Huber, Claudia A. Jette, André Hoelz

**Affiliations:** Division of Chemistry and Chemical Engineering, California Institute of Technology, 1200 East California Boulevard, Pasadena, CA 91125 USA

## Abstract

The nuclear pore complex (NPC) controls the passage of macromolecules between the nucleus and cytoplasm, but how the NPC directly participates in macromolecular transport remains poorly understood. In the final step of mRNA export, the DEAD-box helicase DDX19 is activated by the nucleoporins Gle1, Nup214, and Nup42 to remove Nxf1•Nxt1 from mRNAs. Here, we report crystal structures of Gle1•Nup42 from three organisms that reveal an evolutionarily conserved binding mode. Biochemical reconstitution of the DDX19 ATPase cycle establishes that human DDX19 activation does not require IP_6_, unlike its fungal homologs, and that Gle1 stability affects DDX19 activation. Mutations linked to motor neuron diseases cause decreased Gle1 thermostability, implicating nucleoporin misfolding as a disease determinant. Crystal structures of human Gle1•Nup42•DDX19 reveal the structural rearrangements in DDX19 from an auto-inhibited to an RNA-binding competent state. Together, our results provide the foundation for further mechanistic analyses of mRNA export in humans.

## Abstract (web summary)

The export of mRNA to the cytosol depends on the nuclear pore complex (NPC) and the activation of the helicase DDX19, but their interplay in humans remains poorly understood. Here, the authors present a structural and functional analysis of DDX19 activation, revealing how the human NPC regulates mRNA export.

## Introduction

**Fig. 1:**
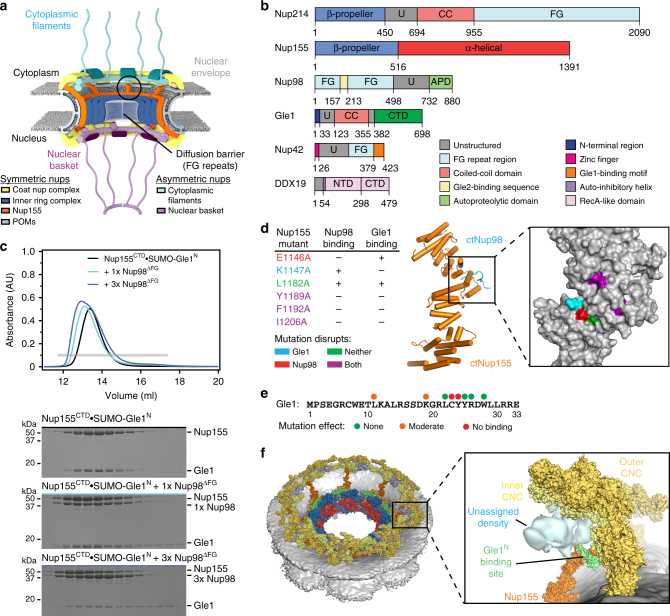
Gle1 is anchored to the nuclear pore complex through a competitive interaction with Nup98. **a** Cartoon schematic of the human nuclear pore complex (NPC). The circle highlights the region of the NPC to which the proteins used in this study localize. **b** Domain schematics for nucleoporins used in this study. Protein names and boundaries correspond to the human proteins. **c** Size exclusion chromatography (SEC) analysis of the interaction between Nup155^CTD^, SUMO-Gle1^N^, and Nup98^∆FG^. Purified Nup155^CTD^**•**SUMO-Gle1^N^ complex was mixed with the indicated amounts of Nup98^∆FG^ and loaded onto a Superdex 200 10/300 GL column. The gray bar indicates the fractions visualized with Coomassie-stained SDS-PAGE gels. **d** Left: table summarizing SEC analysis of Nup155^CTD^ variants for Nup98^∆FG^ and SUMO-Gle1^N^ binding. See also Supplementary Fig. 2. Right: the homologous positions were colored on the *C*. *thermophilum* Nup170•Nup145N structure (PDB ID 5HB0), indicating that the same binding surface is recognized by Nup98^∆FG^ and SUMO-Gle1^N^[2]. **e** Summary of the effect of Gle1^N^ alanine substitution variants on Nup155^CTD^ binding. Colored dots above the sequence of Gle1^N^ indicate the effect of the substitution, green for wild-type levels of complex formation, orange for reduced binding, or red for complete disruption. See also Supplementary Fig. 2. **f** Identification of the Gle1 binding site suggests that the unassigned cytoplasmic density adjacent to bridging Nup155 molecules could contain Gle1 and its binding partners. Left: surface representation of the composite structure of the NPC^2^. Right: zoomed view of unassigned cytoplasmic density, with Nup155 shown in orange, Gle1^N^ binding site colored in green, and coat nucleoporin complexes shown in yellow

The flow of genetic information requires newly transcribed and processed mRNAs to be exported from the nucleus to the cytoplasm through nuclear pore complexes (NPCs). NPCs are massive macromolecular machines perforating the nuclear envelope, each composed of ~1000 protein subunits (collectively termed nucleoporins) totaling to a molecular mass of ~120 MDa in humans^1^. By fusing the inner and outer nuclear membranes, NPCs create pores through the nuclear envelope and simultaneously generate a passive diffusion barrier composed of disordered protein sequences enriched in phenylalanine-glycine (FG) repeats. Each NPC is composed of a ~60 MDa symmetric core that is decorated by different proteins on its nuclear and cytoplasmic faces, which are referred to as the nuclear basket and cytoplasmic filament nucleoporins, respectively (Fig. 1a).

The architecture of the symmetric core of the human NPC has recently been elucidated^2,3^. In humans, the symmetric core is composed of an inner ring that resides in the plane of the membrane and two concentric outer rings that reside above the inner and outer nuclear membranes. The outer rings, which serve as the attachment sites for the nuclear basket and cytoplasmic filaments, are structurally connected to the inner ring via bridging Nup155 molecules. In addition to large interaction interfaces between scaffold proteins, the assembly of the symmetric core requires interactions mediated by flexible linker sequences residing in Nup98, Nup53, and Nup93^2,4,5^. In contrast, the molecular organization of the cytoplasmic filaments and nuclear basket remains poorly understood.

Preparation of mRNAs for nuclear export is a highly coordinated process that begins co-transcriptionally and results in the addition and removal of mRNA-binding proteins during transcription and nuclear processing until an export-competent messenger ribonucleoprotein particle (mRNP) is formed^6^. Although some mRNAs may be exported through specialized pathways, the bulk of mRNA export is mediated by the evolutionarily conserved, heterodimeric transport factor complex Nxf1•Nxt1^7^. Nxf1•Nxt1 binds mRNAs without strong sequence preference and can shuttle mRNAs through the NPC diffusion barrier by binding to FG repeats. At the cytoplasmic face of the NPC, Nxf1•Nxt1-bound mRNPs encounter the cytoplasmic filament nucleoporins Gle1, Nup42, and Nup214, which specifically activate the DEAD-box helicase DDX19 to remove Nxf1•Nxt1 from the mRNP^8^. This spatial regulation of activity prevents re-import of mRNPs into the nucleus, and thus ensures the directionality of mRNA export.

DDX19 is a member of the DEAD-box helicase family, which are RNA-dependent ATPases composed of two RecA domains (referred to as DDX19^NTD^ and DDX19^CTD^ throughout the text) (Fig. 1b). Many insights into the regulation of DDX19 come from studies of the fungal homolog, Dbp5. Genetic, biochemical, and structural studies of the yeast proteins have revealed that the interaction between Gle1 and Dbp5 occurs via their C-terminal domains (CTDs) and is bridged by the small molecule inositol hexaphosphate (IP_6_)^9–12^. In yeast, Gle1, IP_6_, and RNA cooperate to stimulate the ATPase activity of Dbp5, although the precise mechanism remains debated^10,13^. The functional roles of Nup42 and Nup214 are also unclear. The interaction between Nup214 and DDX19 is required for steady-state localization of DDX19 to the NPC, but Nup214 binding also inhibits DDX19 activity^14–17^. Nup42 binds to Gle1, but its contributions to DDX19 activity are unknown^18,19^. DDX19 and Gle1 have been implicated in other cellular processes including transcription regulation, DNA damage response, translation initiation, and RNA processing^20–25^.

Impairment of nucleocytoplasmic transport through the NPC has been linked to both Huntington’s disease and amyotrophic lateral sclerosis (ALS)^26–30^. Nucleocytoplasmic transport factors and nucleoporins, including Gle1, are genetic modifiers of disease in model systems and are mislocalized in both model organism and patient samples^26–29,31,32^. Specific mutations of Gle1 are associated with lethal contracture congenital syndrome 1 (LCCS1), lethal arthrogryposis with anterior horn cell disease (LAAHD), and ALS^33,34^. However, deciphering how defects in nucleocytoplasmic transport can lead to disease will require an improved understanding of the regulation of transport through the NPC.

To gain further mechanistic insight into the role of nucleoporins in mRNA export, we characterized the molecular architecture of the cytoplasmic filament nucleoporins involved in regulating DDX19 activity. We mapped the Gle1-binding site on Nup155 and found that it overlaps with the Nup98 binding site, thereby acquiring a spatial restraint for Gle1 localization in the NPC. Crystal structures of the Gle1•Nup42 complex from *Saccharomyces cerevisiae*, *Chaetomium thermophilum*, and *Homo sapiens* revealed the evolutionarily conserved structural basis of their interaction. Nup42 is critical for the thermostability of human Gle1, enabling Gle1 purification from a recombinant source and characterization of its role in human DDX19 activation, which unlike the yeast system does not require IP_6_ binding. Crystal structures of the human Gle1•Nup42•DDX19 complex bound to ADP and AMP-PNP•Mg^2+^ uncovered the adaptations that facilitate IP_6_-independent activation in humans and the specific Gle1-induced conformational changes that release DDX19 from an auto-inhibited state. Lastly, Gle1 mutations that are associated with motor neuron diseases possess severe thermostability defects, suggesting that nucleoporin misfolding contributes to disease.

## Results

### Gle1 and Nup98 recognize overlapping surfaces on Nup155

To gain a better understanding of the molecular architecture of the nucleoporins that regulate mRNA export, we set out to reconstitute the interactions with purified, recombinant proteins. We use the names of the human proteins unless otherwise specified. In humans, there are two splice variants of the Gle1 transcript, Gle1A and Gle1B, which encode proteins that are primarily localized in the cytoplasm or at the cytoplasmic face of the NPC, respectively^35^. We focused on the NPC-localized Gle1B, referred to as Gle1 throughout the text. Human Gle1 can be divided into three structural domains: an unstructured N-terminal region (Gle1^N^, residues 1−123), a coiled-coil region (Gle1^CC^, residues 124–355), and a highly conserved CTD (Gle1^CTD^, residues 382–698) (Fig. 1b). Gle1^CTD^ is the domain that binds and stimulates DDX19, but previous studies have identified features in all three regions that are important for NPC localization^19,36,37^.

We began our analysis by focusing on the interaction between Gle1 and the adaptor nucleoporin Nup155 (Nup170 in fungi). Although the first 28 residues of Gle1 were previously shown to be sufficient for an interaction, we used a SUMO-fusion construct that also included several charged residues at the C-terminus to enhance protein solubility (SUMO-Gle1^N^, residues 2–33)^37^. The Nup155 CTD (Nup155^CTD^, residues 870–1391) and SUMO-Gle1^N^ formed a stoichiometric complex in size exclusion chromatography (SEC) experiments (Fig. 1c; Supplementary Fig. 1a). Nup155^CTD^ also contains a binding site for Nup98, which is both a component of the symmetric core of the NPC and of the cytoplasmic filaments^2,4^. Stoichiometric complex formation also occurred between Nup155^CTD^ and a construct of Nup98 lacking the FG repeat region (Nup98^∆FG^, residues 498–880) (Supplementary Fig. 1b). However, when we attempted to reconstitute a heterotrimeric complex by adding Nup98^∆FG^ to Nup155^CTD^•SUMO-Gle1^N^, complex formation between Nup155^CTD^ and Nup98^∆FG^ coincided with displacement of SUMO-Gle1^N^, suggesting that the interactions were mutually exclusive (Fig. 1c; Supplementary Fig. 1a). Similarly, addition of SUMO-Gle1^N^ to Nup155^CTD^•Nup98^∆FG^ failed to yield a heterotrimeric complex (Supplementary Fig. 1b). This effect was specific to Nup98, as addition of the N-terminal domain of Nup214 (Nup214^NTD^) to Nup155^CTD^•SUMO-Gle1^N^ did not displace SUMO-Gle1^N^ (Supplementary Fig. 1c). In GST-pull-down experiments, Gle1^N^ efficiently outcompeted Nup98^∆FG^ for Nup155^CTD^ binding, whereas conversely, Nup98^∆FG^ could not outcompete Gle1^N^, suggesting that Gle1^N^ binds with greater affinity to Nup155^CTD^ than Nup98^∆FG^ (Supplementary Fig. 1d).

We next expanded upon previous mutational and structural analyses using the *C. thermophilum* proteins^2^. Amino acid substitutions at positions homologous to those that abolished binding for the *C. thermophilum* proteins also disrupted binding between human Nup155^CTD^ and Nup98^∆FG^, demonstrating that the mechanism of interaction between these two nucleoporins is evolutionarily conserved (Fig. 1d; Supplementary Fig. 2a). Moreover, most residues that were critical for Nup98^∆FG^ binding were also important for Gle1^N^ binding, except for two residues that each affected only one nucleoporin, indicating that Gle1^N^ and Nup98^∆FG^ recognize overlapping surfaces on Nup155^CTD^ (Fig. 1d; Supplementary Fig. 2b). Hydrophobic residues in Gle1^N^ were critical for Nup155^CTD^ binding, consistent with utilization of the same hydrophobic pockets as Nup98^∆FG^ (Fig. 1e; Supplementary Fig. 2c).

In summary, we found that Gle1^N^ and Nup98^∆FG^ recognize overlapping surfaces on Nup155^CTD^. Nup155 molecules bridge the inner ring to the cytoplasmic and nuclear outer coat nucleoporin complex (CNC) double rings (Fig. 1a)^2,3^. Because Gle1^N^ efficiently outcompetes Nup98^∆FG^ for binding, Gle1 would likely prevail for binding to Nup155 molecules exposed to the cytoplasmic face. Indeed, in the cytoplasmic outer ring, but not the nuclear outer ring, there is a volume of unaccounted density directly adjacent to the Nup155^CTD^ surface that binds Gle1^N^ (Fig. 1f). Our analysis suggests this density contains the remainder of the Gle1 molecule and its binding partners.

### Identification of a minimal Nup42 Gle1-binding fragment

**Fig. 2:**
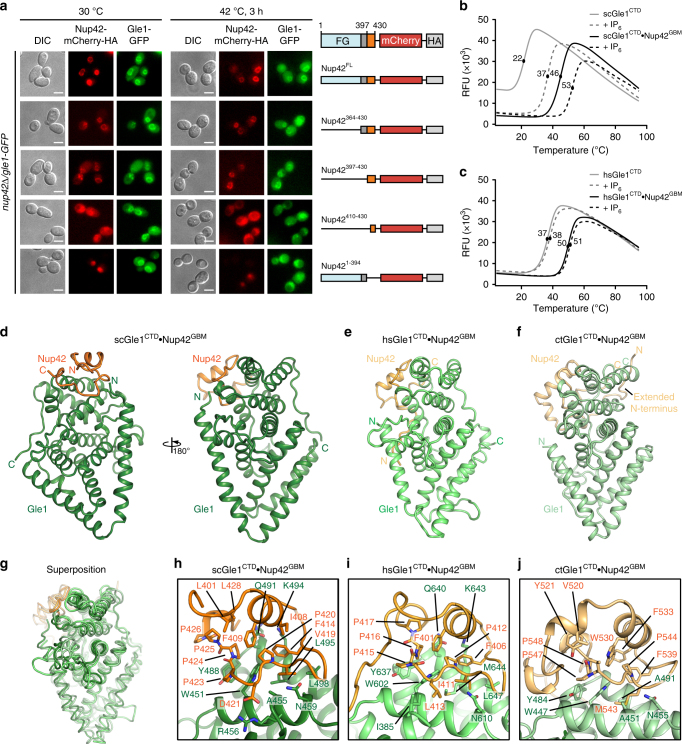
A conserved mechanism for Gle1•Nup42 complex formation. **a** In vivo localization analysis of Gle1-GFP and Nup42-mCherry-HA variants in *nup42∆/gle1-GFP S. cerevisiae* cells. Scale bar is 5 μm. Schematics on the right indicate the Nup42 fragments that were included in the construct, with omitted fragments indicated by replacement of the domain with a solid line. Residue numbers indicate the fragment included in each construct. See also Supplementary Fig. 3. **b**, **c** Differential scanning fluorimetry analysis of **b** *S. cerevisiae* or **c** *H. sapiens* Gle1^CTD^ thermostability in the presence and absence of Nup42^GBM^ and IP_6_. Exposure of hydrophobic residues was monitored by an increase in relative fluorescence units (RFUs). Curves represent the average of three experiments. See also Supplementary Fig. 4. **d**−**f** Crystal structures of **d** *S. cerevisiae*, **e** *H. sapiens*, or **f** *C. thermophilum* Gle1^CTD^•Nup42^GBM^. See also Supplementary Figs. 5 and 8–11. **g** Superposition of the structures of *S. cerevisiae, H. sapiens*, and *C. thermophilum* Gle1^CTD^•Nup42^GBM^, with same coloring as in **d**−**f**. See also Supplementary Movie 1. **h**−**j** Zoom views of **h** *S. cerevisiae*, **i** *H. sapiens*, or **j** *C. thermophilum* Gle1^CTD^•Nup42^GBM^ interactions with residues mediating the interaction labeled

We next focused on the interaction between Gle1^CTD^ and Nup42. Previous studies have shown that the C-terminal, non-FG repeat region of Nup42 binds Gle1^CTD^^19,38^. To identify the minimally sufficient fragment of Nup42 that recognizes Gle1, we monitored the localization of Gle1-GFP and mCherry-HA-tagged Nup42 variants in *S*. *cerevisiae*^39^. Nup42 truncations that contained residues 397–430 displayed nuclear rim staining consistent with localization to the NPC, whereas an Nup42 variant containing only residues 410–430 did not (Fig. 2a). From these results, we concluded that the Nup42 Gle1-binding motif (Nup42^GBM^) is located within residues 397–430 (See Supplementary Note 1).

When we recombinantly purified the minimal *S. cerevisiae* Gle1^CTD^•Nup42^GBM^ complex, we noticed that the purified complex was more stable than *apo* Gle1^CTD^, which typically requires the addition of IP_6_ in purification buffers for stability^10^. This observation led us to test the effect of IP_6_ and Nup42^GBM^ on the stability of Gle1^CTD^ using two different assays: differential scanning fluorimetry and a protein solubility assay. In both experiments, IP_6_ potently improved Gle1^CTD^ stability, with saturating amounts of IP_6_ shifting the melting temperature (*T*_m_) from 22 to 37 °C (Fig. 2b; Supplementary Fig. 4a). Nup42^GBM^ had an even more dramatic effect, increasing the *T*_m_ from 22 to 46 °C, and the presence of both IP_6_ and Nup42^GBM^ shifted the *T*_m_ of Gle1^CTD^ to 53 °C (Fig. 2b; Supplementary Fig. 4a).

The difficulty of purifying human Gle1^CTD^ has previously prevented a detailed biochemical analysis of the human proteins, but the dramatic effect of yeast Nup42^GBM^ on Gle1^CTD^ stability led us to test whether human Nup42^GBM^ has the same effect on human Gle1^CTD^. Co-purification of a homologous human Gle1^CTD^•Nup42^GBM^ complex (containing Gle1 residues 382−698 and Nup42 residues 379−423) resulted in dramatic improvements in the stability of Gle1^CTD^ and the yields increased from ~0.5 to ~50 mg/100 l of bacterial culture. In thermostability experiments, the *T*_m_ of human Gle1^CTD^ increased from 37 to 50 °C in the presence of Nup42^GBM^ (Fig. 2c; Supplementary Fig. 4b). However, in contrast to the yeast proteins, IP_6_ had almost no effect on thermostability for human Gle1^CTD^ (Fig. 2c; Supplementary Fig. 4b). In summary, we identified an evolutionarily conserved C-terminal fragment of Nup42 that binds Gle1 and has a profound effect on Gle1 stability in both yeast and humans.

### Evolutionary conservation of the Gle1−Nup42 interaction

To understand the molecular basis for the interaction between Gle1^CTD^ and Nup42^GBM^ and the resulting stabilization of Gle1^CTD^, we determined the crystal structure of *S. cerevisiae* Gle1^CTD^•Nup42^GBM^ at 1.75 Å resolution (Fig. 2d; Supplementary Table 1). Nup42^GBM^ folds into a compact domain with a hydrophobic core that buries a solvent-exposed hydrophobic surface on Gle1^CTD^, yielding an interaction interface area of ~835 Å^2^ (Fig. 2d; Supplementary Fig. 5a; See Supplementary Note 2 for details). Consistent with the extensive interaction surface, the interaction between Gle1^CTD^ and Nup42^GBM^ was robust against several individual alanine substitutions in SEC experiments (Supplementary Fig. 6a). Instead, highly disruptive substitutions that introduced negative charge into the hydrophobic core (F414D or F409D/F414D) were required to completely disrupt the interaction between Gle1^CTD^ and Nup42^GBM^ (Supplementary Fig. 6b). In agreement with these results, Nup42 variants containing the F414D or F409D/F414D substitutions were unable to rescue Nup42 deletion in *S. cerevisiae* (Supplementary Fig. 3). In contrast, the F409D substitution in the Nup42 hydrophobic core did not ablate binding, but did alter the elution profile, suggesting a conformational difference in the complex (Supplementary Fig. 6b). Accordingly, an Nup42 variant harboring the F409D substitution only had a mild effect on growth at 37 °C (Supplementary Fig. 3a).

To evaluate whether the mode of interaction observed for *S. cerevisiae* Gle1^CTD^•Nup42^GBM^ was conserved in other eukaryotes, we determined the crystal structures of human Gle1^CTD^•Nup42^GBM^ at 2.8 Å resolution and of *C. thermophilum* Gle1^CTD^•Nup42^GBM^ in the presence and absence of IP_6_ at 2.17 and 2.65 Å resolution, respectively (Fig. 2e, f; Supplementary Fig. 7a; Supplementary Tables 1 and 2). The overall structure of Gle1^CTD^ is conserved between fungi and humans, with minor differences resulting from small insertions or different loop sizes (Fig. 2g; Supplementary Fig. 8 and Supplementary Movie 1). Both human and *C. thermophilum* Nup42^GBM^ adopt the same fold as *S. cerevisiae* Nup42^GBM^ and recognize the same surface on Gle1^CTD^, with the critical hydrophobic residues nearly universally conserved (Fig. 2i, j; Supplementary Figs. 8–11; See Supplementary Note 2 for details). Many of the critical interaction interfaces in the NPC likely possess a similar degree of structural conservation. Burial of the exposed hydrophobic residues and the thermodynamic favorability of Nup42^GBM^ binding and folding explain the large effect Nup42^GBM^ has on Gle1^CTD^ stability. Nup42^GBM^ binding may also help prevent Gle1^CTD^ from sampling partially unfolded states that could lead to aggregation. The remainder of Nup42 is comprised primarily of FG repeats. In yeast, deletion of these FG repeats was detrimental when combined with the deletion of other FG repeats in the cytoplasmic filaments^40^. Thus, in addition to ensuring the stability of Gle1^CTD^, Nup42^GBM^ also has a role in anchoring FG repeats proximal to Gle1 in the NPC.

### The IP_6_ binding pocket is not conserved in metazoan Gle1

**Fig. 3:**
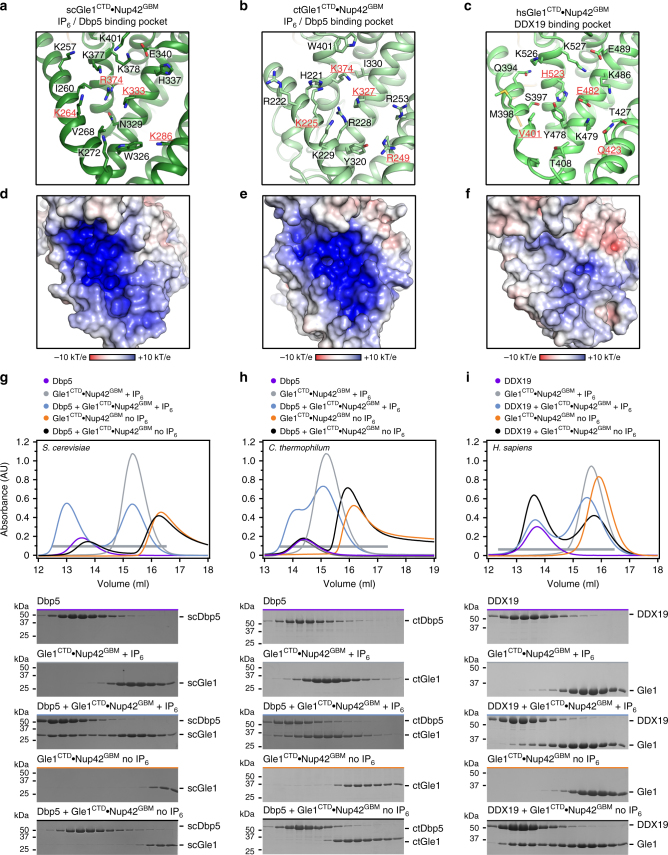
Human Gle1^CTD^ binding to DDX19 is IP_6_ independent. **a**−**c** Zoom view of the IP_6_ binding pocket of **a** *S. cerevisiae*, **b** *C. thermophilum*, or **c** *H. sapiens* Gle1^CTD^. Residues that are conserved in fungi but not metazoans are highlighted in red. See also Supplementary Figs. 7 and 12. **d**−**f** Surface electrostatic potential analysis of IP_6_ binding pockets for **d** *S. cerevisiae*, **e** *C. thermophilum*, or **f** *H. sapiens* Gle1. The same view as **a**−**c** is shown in surface representation colored by electrostatic potential, from red (−10 k_B_T/e) to white (0 k_B_T/e) to blue (+10 k_B_T/e), revealing a dramatically reduced electrostatic potential for human Gle1^CTD^. See also Supplementary Figs. 5, 10, and 11. **g**−**i** SEC analysis of the interaction between Dbp5/DDX19 and Gle1^CTD^•Nup42^GBM^ for **g** *S. cerevisiae*, **h** *C. thermophilum*, or **i** *H. sapiens*, in the presence or absence of IP_6_. The elution profiles for Dbp5/DDX19 are shown in purple, Gle1^CTD^•Nup42^GBM^ and IP_6_ are shown in gray, Gle1^CTD^•Nup42^GBM^ without IP_6_ are shown in orange, Dbp5/DDX19 with Gle1^CTD^•Nup42^GBM^ and IP_6_ are shown in light blue, and Dbp5/DDX19 with Gle1^CTD^•Nup42^GBM^ without IP_6_ are shown in black. Fungal Gle1^CTD^•Nup42^GBM^ interacts strongly with the Superdex matrix in the absence of IP_6_. The gray horizontal bar indicates fractions visualized with Coomassie-stained SDS-PAGE gels shown below

In yeast, activation of Dbp5, the fungal homolog of DDX19, requires the small molecule IP_6_, which binds to a highly positively charged pocket in Gle1^CTD^ adjacent to the Dbp5 binding surface and bridges the two proteins^9–11^. Our Gle1^CTD^•Nup42^GBM^ structure reveals that Nup42^GBM^ interacts with Gle1^CTD^ at a surface that is distinct and well separated from the IP_6_ binding pocket and the Dbp5 interface (Supplementary Fig. 5a)^10^. Thus, in order for Nup42^GBM^ binding to affect Dbp5 activation, it would have to do so through an allosteric mechanism. However, there were minimal conformational differences in Gle1^CTD^ between our Gle1^CTD^•Nup42^GBM^ structure and the previously reported structure of Gle1^CTD^•IP_6_•Dbp5 (Supplementary Fig. 7b)^10^. Similarly, *C. thermophilum* Gle1^CTD^•Nup42^GBM^ undergoes minimal conformational changes upon IP_6_ binding, which are mostly limited to a loop directly adjacent to the IP_6_ pocket. IP_6_ binds to *C. thermophilum* Gle1^CTD^ and to *S. cerevisiae* Gle1^CTD^ in a similar orientation, suggesting that IP_6_ could function similarly in *C. thermophilum* as in *S. cerevisiae* (Fig. 3a, b, d, e; Supplementary Fig. 7d; See Supplementary Note 3). However, because there are no *apo* Gle1^CTD^ structures available, we could not exclude the possibility that Nup42^GBM^ binding caused Gle1^CTD^ to adopt the same conformation as the one observed upon IP_6_ and Dbp5 binding.

In contrast to the IP_6_ binding pockets of *S. cerevisiae* and *C. thermophilum* Gle1^CTD^, the same location on the surface of the *H. sapiens* molecule was significantly altered, resulting in a substantial reduction in positive electrostatic potential (Fig. 3a−f). This was the consequence of several positively charged residues that are essentially invariant in fungi (*S. cerevisiae* residues K264, K286, K333, and R374; *C. thermophilum* residues K225, R249, K327, and K374), but are not conserved in humans (V401, Q423, E482, and H523) (Fig. 3a−c). This trend held for all the metazoan sequences inspected, indicating that the altered electrostatic potential in this pocket may be a general feature of metazoan Gle1 (Supplementary Fig. 12a). Although there are two positions in human Gle1 that contained lysine residues not present in the fungal proteins, sequence conservation analysis also indicated that the positively charged residues in Dbp5 that bind IP_6_ were not conserved in metazoans (Supplementary Fig. 12b). Combined, these structural observations raised the question of what roles Nup42 and IP_6_ had on the activation of human DDX19.

### The roles of IP_6_ and Nup42 in DDX19/Dbp5 stimulation

To assess the role of IP_6_ in DDX19 activation, we first tested whether complex formation between human DDX19 and Gle1 was dependent on IP_6_ in SEC experiments. Consistent with previous reports, *S. cerevisiae* Dbp5 and Gle1^CTD^•Nup42^GBM^ only formed a complex in the presence of IP_6_ (Fig. 3g)^10^. The interaction between *C. thermophilum* Gle1^CTD^•Nup42^GBM^ and Dbp5 also required IP_6_, consistent with the evolutionary conservation of the IP_6_ binding residues among fungi (Fig. 3h,; Supplementary Fig. 12a). In contrast, complex formation of the human proteins did not require IP_6_, and the presence of IP_6_ instead partially reduced complex formation (Fig. 3i).

To directly test the effect of IP_6_ and Nup42 on Dbp5 and DDX19 activity, we measured steady-state ATP hydrolysis rates with an NADH-coupled reaction using conditions identical to those previously reported^41^. Due to concerns that observed differences in activity were due to the stabilizing effects of IP_6_ and Nup42 on Gle1 rather than direct roles in Dbp5/DDX19 activation, we measured ATP hydrolysis rates at multiple temperatures to decouple the effects due to stabilization from bona fide stimulatory roles. Because human DDX19 was intrinsically less active than *S. cerevisiae* Dbp5, we used fivefold higher concentrations of human DDX19, Gle1^CTD^, Nup42^GBM^, and IP_6_ to ensure accurate measurements of ATPase activity. Furthermore, because Dbp5 and DDX19 are slow ATPases, we developed extensive protein purification protocols to ensure all of the measured activity was directly attributable to Dbp5 or DDX19 (Supplementary Fig. 13a, b).

**Fig. 4:**
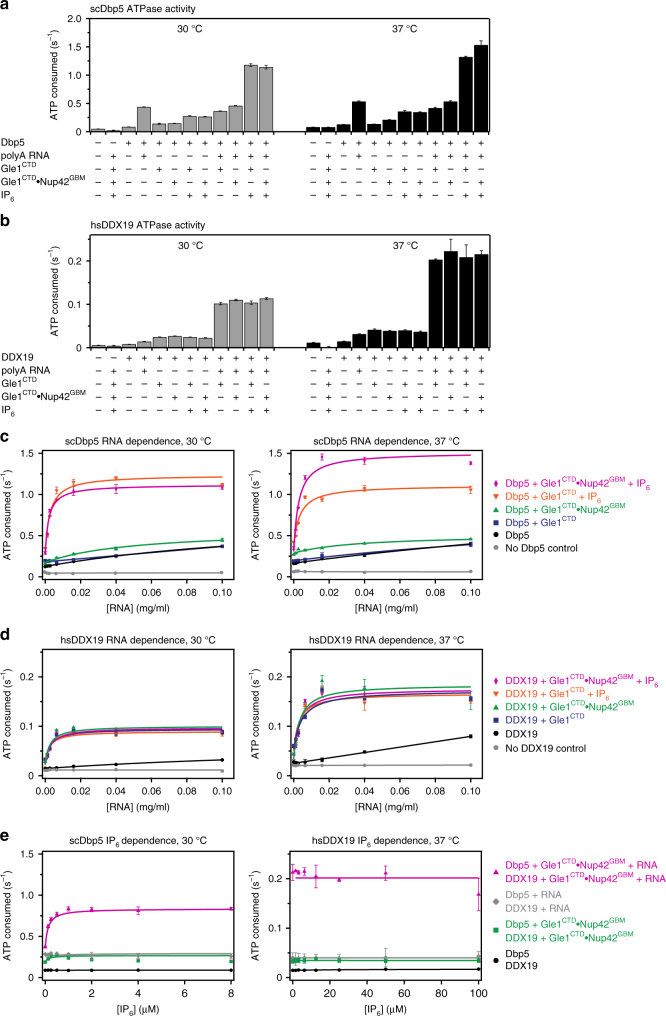
Role of Nup42, RNA, and IP_6_ in Dbp5/DDX19 activation. **a** Steady-state colorimetric ATPase assay with *S. cerevisiae* Dbp5 performed at 30 and 37 °C with either purified *S. cerevisiae* Gle1^CTD^ or Gle1^CTD^•Nup42^GBM^. Reactions were performed with 0.5 μM Dbp5, 1.0 μM Gle1^CTD^ or Gle1^CTD^•Nup42^GBM^, 0.1 mg/ml polyA RNA, and 2.0 μM IP_6_. Values shown are the average of three experiments. Error bars indicate the standard deviation. See also Supplementary Fig. 13. **b** Steady-state colorimetric ATPase assay with *H. sapiens* DDX19 performed at 30 and 37 °C with either purified *H. sapiens* Gle1^CTD^ or Gle1^CTD^•Nup42^GBM^. Reactions were performed with 2.5 μM DDX19, 5.0 μM Gle1^CTD^ or Gle1^CTD^•Nup42^GBM^, 0.1 mg/ml polyA RNA, and 10.0 μM IP_6_. Values shown are the average of three experiments. Error bars indicate the standard deviation. See also Supplementary Fig. 13. **c** RNA dependence of *S*. *cerevisiae* Dbp5 activation using the same conditions as in **a**, but with the indicated amounts of RNA performed at 30 and 37 °C. **d** RNA dependence of *H. sapiens* DDX19 activation using the same conditions as in **b**, but with the indicated amounts of RNA performed at 30 and 37 °C. **e** IP_6_ dependence of (left) *S*. *cerevisiae* Dbp5 or (right) *H*. *sapiens* DDX19 activation using the same conditions as in **a** or **b**, respectively, but with the indicated amounts of IP_6_

In the yeast system, and across a range of RNA concentrations, the addition of Nup42^GBM^ to Gle1^CTD^ increased Dbp5 activity at 37 °C, but this stimulatory effect was greatly diminished at 30 °C, a temperature below the *T*_m_ of Gle1^CTD^•IP_6_, which is consistent with thermostability having a detectable effect in our assays (Fig. 4a, c; Supplementary Fig. 13c). Altogether, these data indicate that Nup42^GBM^ does not have a direct role in Dbp5 stimulation in the yeast system, but rather functions primarily to ensure Gle1 stability. In the yeast system, IP_6_ was required for full stimulation of Dbp5 at all temperatures tested, indicating that in addition to its ability to stabilize yeast Gle1^CTD^, IP_6_ also has a direct role in Dbp5 stimulation (Fig. 4a; Supplementary Fig. 13c, d). These results are in agreement with the observation that stable association of Gle1^CTD^ and Dbp5 requires IP_6_ binding. In contrast, neither Nup42^GBM^ nor IP_6_ were stimulatory in the human system at either temperature we tested (Fig. 4b, d; Supplementary Fig. 13e).

Because differences in the IP_6_ binding pocket in human Gle1 and DDX19 could have weakened the affinity for IP_6_ rather than completely abolishing binding, we tested whether higher concentrations of IP_6_ would stimulate DDX19. IP_6_ stimulated Dbp5 activity in a dose-dependent manner that saturated at 1 μM (twice the Dbp5 concentration), but had no effect on human DDX19 activity up to concentrations of 100 μM, which is an upper estimate for the total (bound and free) IP_6_ concentration in human cells (Fig. 4e)^42^. Additionally, IP_6_ bound tightly to *S*. *cerevisiae* Gle1^CTD^•Nup42^GBM^ in isothermal titration calorimetry experiments (ITC) with a dissociation constant (*K*_d_) of ~3 nM. However, no binding of IP_6_ to human Gle1^CTD^•Nup42^GBM^ was detected up to concentrations of 200 μM (Supplementary Fig. 14). Taken together, our results indicate that IP_6_ binding is conserved in fungi but not in humans. Moreover, sequence analysis of other metazoan Gle1 and DDX19 sequences suggests that IP_6_ binding may not be a feature of metazoan DDX19 activation in general, as IP_6_-binding residues are not present in a diverse array of metazoan sequences (Supplementary Fig. 12a). These results do not exclude the possibility that another small molecule may serve a similar function in humans as IP_6_ does in fungi. However, unlike their fungal homologs, the human proteins alone are sufficient for complex formation.

### Structural basis for IP_6_-independent human DDX19 activation

**Fig. 5:**
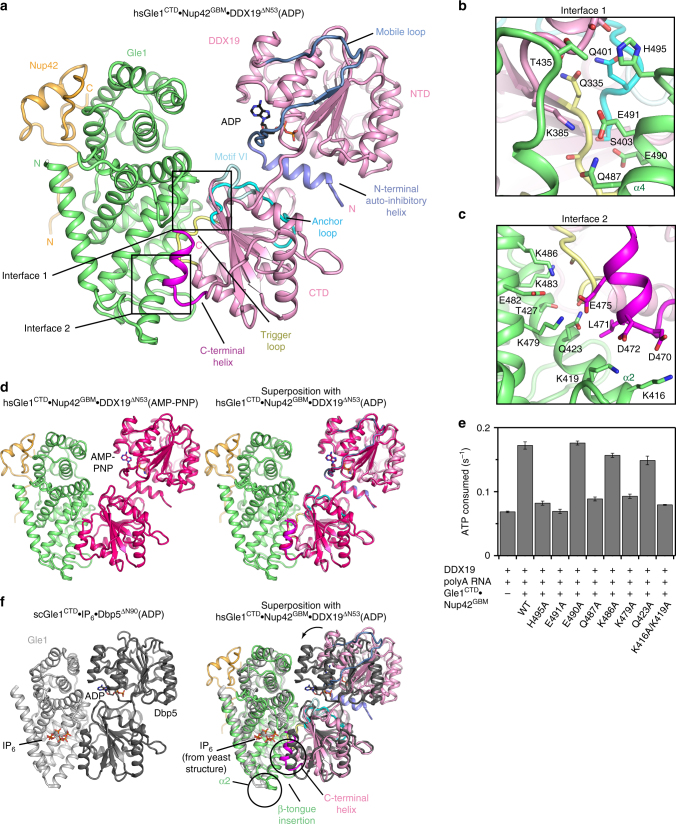
Structure of the human Gle1^CTD^•Nup42^GBM^•DDX19^∆N53^ complex. **a** Crystal structure of *H. sapiens* Gle1^CTD^•Nup42^GBM^•DDX19^∆N53^(ADP). Motifs of interest are colored and labeled: auto-inhibitory helix (residues 54–67, purple); mobile loop (residues 68–91, blue); trigger loop (residues 328–335, yellow); anchor loop (residues 390–403, cyan); motif VI (residues 429–435, light cyan); C-terminal helix (residues 468–479, magenta). Boxes indicate the regions shown to the right. **b**, **c** Close-up views of critical complex-forming interactions in interfaces 1 and 2. **d** Left: crystal structure of *H. sapiens* Gle1^CTD^•Nup42^GBM^•DDX19^∆N53^(AMP-PNP•Mg^2+^). DDX19 is colored magenta for clarity. Right: Superposition of the ADP and AMP-PNP•Mg^2+^ bound structures. **e** Analysis of the effect of single amino acid substitutions in the Gle1-DDX19 interface on Gle1-mediated stimulation of DDX19, using the same conditions as in Fig. 4b at 37 °C. Values shown are the average of three experiments. Error bars indicate standard deviation. **f** Left: crystal structure of *S. cerevisiae* Gle1^CTD^•IP_6_•Dbp5^∆N90^(ADP) (PDB ID 3RRN)^10^. Right: superposition of the *S. cerevisiae* and *H. sapiens* structures. The arrow indicates the rotation relating the conformations observed in the two crystal structures. Circles highlight structural differences. See also Supplementary Fig. 17

To understand the structural basis for IP_6_-independent DDX19 activation, we determined crystal structures of human Gle1^CTD^•Nup42^GBM^ in complex with DDX19 in the presence of ADP or AMP-PNP•Mg^2+^ at 3.6 and 3.4 Å resolution, respectively (Fig. 5a, d; Supplementary Table 3). Previous structural studies have revealed that DDX19 possesses an auto-inhibitory helix N-terminal to DDX19^NTD^ (residues 54–67) that can bind between DDX19^NTD^ and DDX19^CTD^, preventing RNA binding or formation of a catalytically competent active site^43^. This helix appears to be conserved among metazoan DDX19 sequences, but is absent in their fungal Dbp5 homologs (Supplementary Fig. 15). Therefore, we used a construct of DDX19 that contained the N-terminal auto-inhibitory helix but did not contain the flexible N-terminal extension (DDX19^∆N53^, residues 54–479). In addition, we determined the crystal structure of *apo* DDX19^∆N53^(AMP-PNP•Mg^2+^) at 2.2 Å resolution (Supplementary Fig. 16a; Supplementary Table 3).

The heterotrimeric Gle1^CTD^•Nup42^GBM^•DDX19^∆N53^ complex exhibited similar conformations in the presence of ADP or AMP-PNP•Mg^2+^, with the auto-inhibitory helix still bound between DDX19^NTD^ and DDX19^CTD^ (Fig. 5a, d). The surprising observation that AMP-PNP-bound DDX19 could also adopt the auto-inhibited conformation was confirmed by the structure of *apo* DDX19^∆N53^(AMP-PNP•Mg^2+^), which adopted an identical conformation as *apo* DDX19^∆N53^(ADP) (Supplementary Fig. 16a)^43^. The inhibited state does not differ structurally in the presence of ADP or ATP, as the nucleotide-binding pocket readily accommodates the additional phosphate and Mg^2+^ ion, with minor changes in the sidechain conformations of K64 from the auto-inhibitory helix and E243 in the nucleotide binding pocket (Supplementary Fig. 16b).

**Fig. 6:**
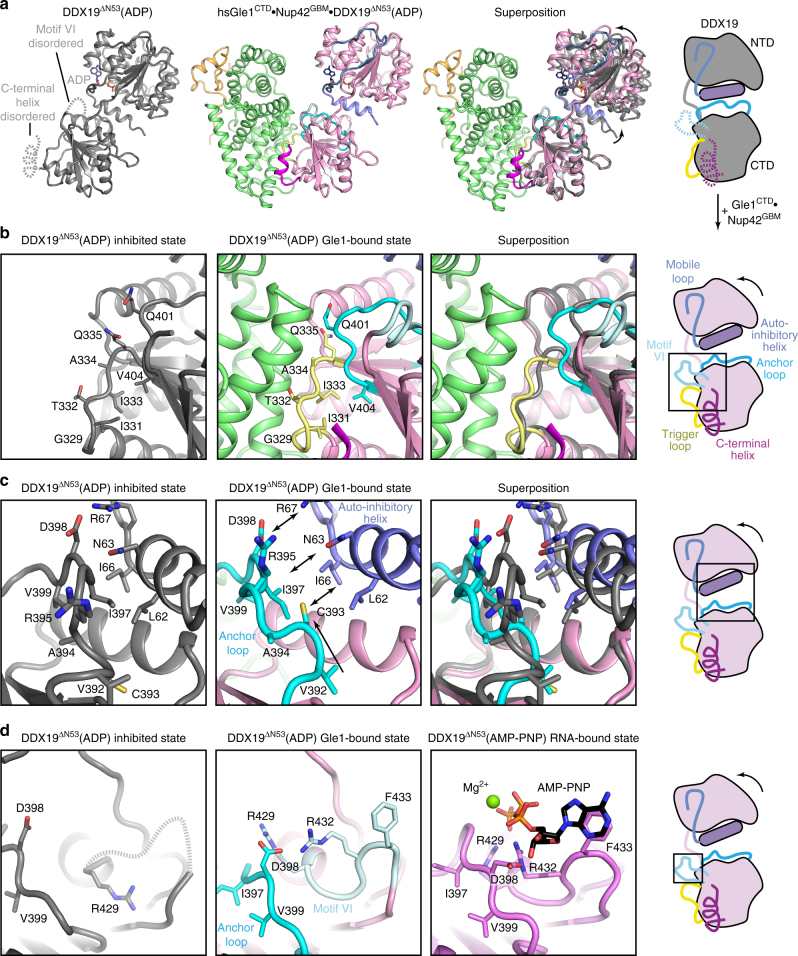
Conformational changes in DDX19 induced by Gle1 binding. **a** Left: crystal structure of *H. sapiens* DDX19^∆N53^(ADP) (PDB ID 3EWS)^44^. Disordered regions (C-terminal helix and motif VI) are indicated with dashed lines. Middle: crystal structure of *H. sapiens* Gle1^CTD^•Nup42^GBM^•DDX19^∆N53^(ADP) shown in the same orientation and colored as in Fig. 5a. Right: superposition of the two structures. Arrows indicate the rotation relating the conformations of DDX19^∆N53^(ADP) in the presence and absence of Gle1^CTD^•Nup42^GBM^. The cartoon on the right schematizes the transition from the inhibited state to the Gle1-bound state. See also Supplementary Fig. 16. **b** Zoom view of the DDX19 trigger loop (yellow) in (left) the inhibited state (PDB ID 3EWS), (middle) the Gle1-bound state, and (right) their superposition. The cartoon on the right indicates the region of DDX19 shown. **c** Zoom view of the DDX19 anchor loop (cyan) and auto-inhibitory helix (purple) in the (left) inhibited state (PDB ID 3EWS), (middle) the Gle1-bound state, and (right) their superposition. The cartoon on the right indicates the region of DDX19 shown. **d** Zoom view of DDX19 Motif VI in (left) the inhibited state (PDB ID 3EWS), (middle) the Gle1-bound state, and (right) the RNA-bound state (PDB ID 3G0H)^44^. The cartoon on the right indicates the region of DDX19 shown

The Gle1^CTD^−DDX19^CTD^ interface involves 40 Gle1 and 35 DDX19 residues to generate an interaction interface area of almost 1300 Å^2^ (Supplementary Fig. 10). As in the yeast complex, the human Nup42^GBM^ and DDX19 binding sites are on opposite sides of the Gle1^CTD^ molecule (Fig. 5a). Most of the solvent exposed residues in helices α2 and α4 from Gle1^CTD^ are buried by the interaction involving two major interaction interfaces (Fig. 5b, c). In the first interface (interface 1), Gle1^CTD^ residues of helix α4 (H495, E491, and Q487) form an extensive hydrogen bond network with each other and DDX19^CTD^ main chain atoms (residues 380–382), as well as a salt bridge between Gle1^CTD^ residue E491 and DDX19^CTD^ residue K385 (Fig. 5b). In the second interface (interface 2), the acidic C-terminal helix of DDX19^CTD^ is recognized by lysines on the Gle1^CTD^ surface, with salt bridges forming between DDX19^CTD^ residues D470, D472, and E475 and Gle1^CTD^ residues K416, K419, and K479 (Fig. 5c). In several instances, DDX19^CTD^ residues (G329, T332, A334, and L471) packed directly against Gle1^CTD^ helices, interdigitating between the sidechains to form hydrophobic interactions (Fig. 6b). Single amino acid substitutions in Gle1^CTD^ at these interfaces were sufficient to disrupt Gle1^CTD^-mediated stimulation of DDX19 (Fig. 5e).

We next searched for the structural differences between the human and yeast complexes that allow the human complex to form in the absence of IP_6_ (Fig. 5f). In the structure of *S. cerevisiae* Gle1^CTD^•IP_6_•Dbp5^∆N90^(ADP), the interface between Gle1^CTD^ and Dbp5^CTD^ is only ~700 Å^2^ in surface area, compared to ~1300 Å^2^ for the human complex, a difference compensated for in yeast through extensive contacts made by both proteins with a bridging IP_6_ molecule (Supplementary Figs. 5a, 10a). For a summary of differences, see Supplementary Note 4. These observed structural differences result in a dramatic reduction of the local electrostatic potential at the human Gle1^CTD^−DDX19^CTD^ interface compared to the yeast Gle1^CTD^−DDX19^CTD^ interface (Supplementary Fig. 17a, b). Thus, for *S*. *cerevisiae* Dbp5 activation, the extreme negative charge of the IP_6_ molecule would be necessary to overcome the electrostatic repulsion generated in forming such a positively charged pocket, but for human DDX19 this requirement no longer exists. To test whether IP_6_ was indeed unable to bind, we also determined crystal structures of the human Gle1^CTD^•Nup42^GBM^•DDX19^∆N53^ complex grown in the presence of IP_6_ or soaked with IP_6_, but observed no electron density for IP_6_ in these crystals (Supplementary Fig. 17d, e; Supplementary Table 4).

To gain further insight into how Gle1^CTD^•Nup42^GBM^ binding facilitates DDX19 activation, we compared the structures of Gle1^CTD^•Nup42^GBM^•DDX19^∆N53^ to those of *apo* DDX19^∆N53^. The most apparent difference was a rotation of both DDX19^NTD^ and the auto-inhibitory helix away from DDX19^CTD^, despite the auto-inhibitory helix still being bound between the two domains (Fig. 6a). In the structure of *S. cerevisiae* Gle1^CTD^•IP_6_•Dbp5^∆N90^(ADP), Dbp5^NTD^ is rotated even further away from Dbp5^CTD^ leading Dbp5^NTD^ to also contact Gle1^CTD^ (Fig. 5f)^10^. In yeast, a Gle1^CTD^ triple amino acid substitution (V513D/A516D/I520D) at the Dbp5^NTD^ binding interface abolishes Gle1-mediated stimulation of Dbp5^10^. A homologous triple amino acid substitution in human Gle1^CTD^ (G666D/I669D/Q673D, DDD mutant) similarly abolished Gle1^CTD^-mediated stimulation of DDX19, suggesting that the human proteins can also form the fully rotated conformation observed for the *S*. *cerevisiae* complex (Supplementary Fig. 13f). Therefore, we considered our structure as an early intermediate state that forms immediately after Gle1^CTD^ binding to DDX19, but before release of the auto-inhibitory helix and separation of DDX19^NTD^ and DDX19^CTD^.

We next searched for the conformational changes in DDX19^CTD^ that might explain how Gle1^CTD^-binding would stimulate ATPase activity. Several differences could be attributed to Gle1^CTD^ binding, including contacts causing ordering of the C-terminal helix and rearrangement of the trigger loop (residues 328–335) (Fig. 6a, b). Through direct interactions between neighboring residues and relief of steric constraints, these changes propagated to the adjacent anchor loop (residues 390–403) (Fig. 6a, b). Remodeling of the anchor loop removes contacts between DDX19^CTD^ and the auto-inhibitory helix, resulting in partial separation of DDX19^NTD^ and DDX19^CTD^ (Fig. 6c). Remodeling of the anchor loop also results in ordering of a loop containing ATP-binding residues in DEAD-box motif VI (Fig. 6d). For details, see Supplementary Note 5. In summary, Gle1^CTD^ binding to DDX19^CTD^ causes a cascade of conformational changes that partially releases the auto-inhibitory helix and prepares the residues involved in nucleotide binding to form the closed, active conformation.

### Human Gle1•Nup42 relieves auto-inhibition

Our structural data are consistent with a mechanism wherein relief from inhibition by the N-terminal auto-inhibitory helix is one of the primary mechanisms for Gle1^CTD^ stimulation of DDX19 activity. Removal of the N-terminal 90 residues of *S. cerevisiae* Dbp5 increases unstimulated Dbp5 activity, but does not increase Gle1-stimulated activity^10^. Similarly, removal of the N-terminal 53 residues also increased DDX19 activity, but activity in the presence of Gle1^CTD^ could not be examined^43^. To obtain a better understanding of the role of various regulatory elements in the N-terminal region of human DDX19, we tested the activation of a series of truncation variants that included DDX19^∆N53^, DDX19^∆N67^, DDX19^∆N91^, and a variant of full-length DDX19, DDX19^S60D/K64D^, containing two aspartate substitutions in the auto-inhibitory helix that would disrupt the inhibited state (Supplementary Fig. 18a; See Supplementary Note 6 for a detailed description of variants).

**Fig. 7:**
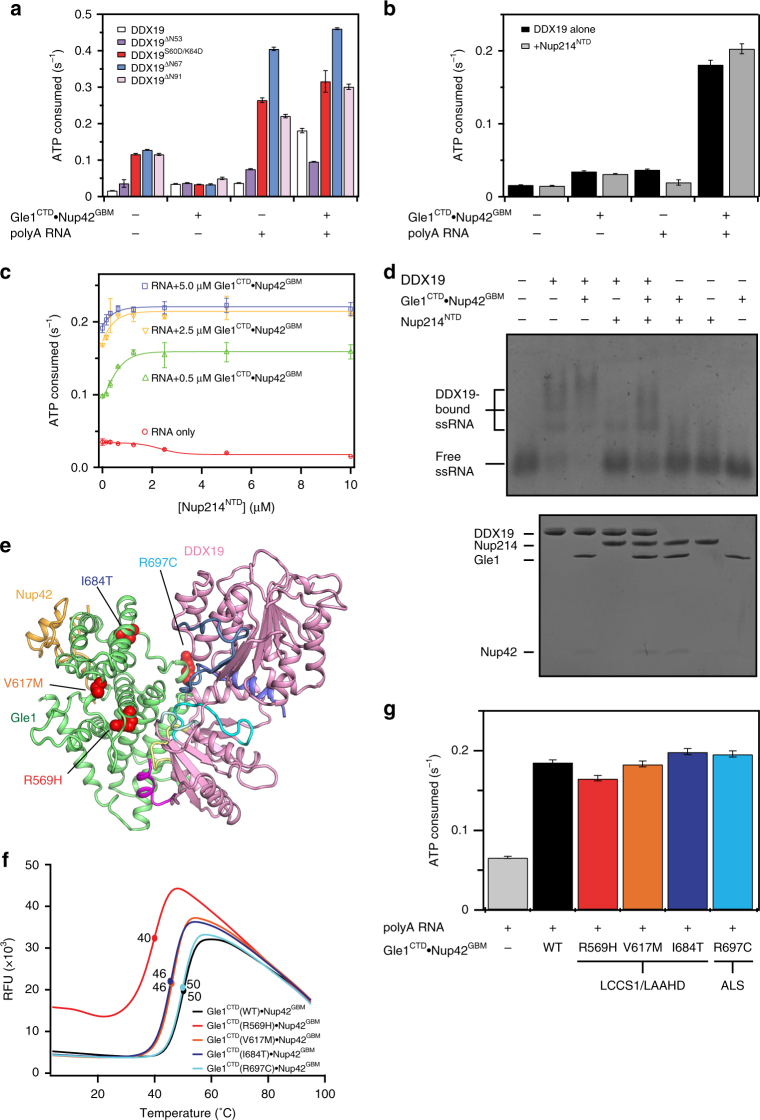
Biochemical analysis of DDX19 activity. **a** Steady-state ATPase activity of DDX19 variants in the presence and absence of RNA and Gle1^CTD^•Nup42^GBM^. For schematics of the various truncation constructs, see Supplementary Fig. 18a. **b** Analysis of the effect of Nup214^NTD^ on DDX19 stimulation. See also Supplementary Fig. 18. **c** Analysis of concentration dependence of Nup214^NTD^ on DDX19 stimulation in the presence of RNA and Gle1^CTD^•Nup42^GBM^. **d** Electrophoretic mobility shift assay analysis of the effect of Gle1^CTD^•Nup42^GBM^ (1 μM) and Nup214^NTD^ (2 μM) on DDX19 (1 μM) binding to a 53-nucleotide ssRNA. RNA was visualized by staining with SYBR gold. The proteins loaded in each lane were also visualized with a Coomassie-stained SDS-PAGE gel, shown below. **e** Mapping single amino acid substitutions associated with human diseases LCCS1/LAAHD and ALS onto the structure of Gle1^CTD^•Nup42^GBM^•DDX19^∆N53^(ADP). Coloring is the same as in Fig. 5a. See also Supplementary Fig. 19. **f** Differential scanning fluorimetry analysis of the effect of human disease mutations on Gle1^CTD^•Nup42^GBM^ stability. Exposure of hydrophobic residues monitored by an increase in relative fluorescence units (RFUs). Curves represent the average of three experiments. **g** Steady-state ATPase activity of DDX19 stimulation by Gle1^CTD^ variants containing single amino acid substitutions associated with human disease. All reported values are the average of three experiments. Error bars indicate standard deviation

All four variants had higher basal ATPase activity than wild-type DDX19, consistent with inhibitory roles for the disrupted sequences (Fig. 7a). Removal of the entire unstructured N-terminal extension, DDX19^∆N53^, yielded ~2.5-fold higher basal ATPase activity, which was similar to the reported ~3-fold higher basal ATPase activity upon removal of the entire Dbp5 N-terminal extension (Fig. 7a)^10^. Further N-terminal truncations, DDX19^∆N67^ and DDX19^∆N91^, as well as the DDX19^S60D/K64D^ double amino acid substitution, exhibited ~7.5-fold higher basal activity (Fig. 7a). Strikingly, in contrast to wild-type DDX19, the activity of all four DDX19 variants was not stimulated by Gle1^CTD^•Nup42^GBM^, but instead was inhibited to an activity level comparable to wild-type DDX19 (Fig. 7a). Moreover, RNA stimulated DDX19 activity in the absence of Gle1^CTD^•Nup42^GBM^ for the DDX19 variants, yielding rates between ~2-fold to ~10-fold faster than wild-type DDX19 (Fig. 7a). For wild-type DDX19, addition of both RNA and Gle1^CTD^•Nup42^GBM^ resulted in greater stimulation than either alone. However, for the variants that perturbed the auto-inhibitory helix, addition of both RNA and Gle1^CTD^•Nup42^GBM^ yielded only a barely detectable further stimulation over RNA-mediated levels, resulting in fully stimulated activities ~2- to 3-fold faster than wild-type DDX19 (Fig. 7a). In contrast, the truncation variant that still possessed an intact auto-inhibitory helix, DDX19^∆N53^, exhibited a ~2-fold slower fully stimulated activity than wild-type.

These data explain several features of DDX19 activation. First, although human DDX19 has ~7.5-fold lower ATPase activity than Dbp5, a large part of this difference can be attributed to the auto-inhibitory helix present in human DDX19 but not yeast Dbp5, as disruption of this helix yields activities only ~3-fold slower than fully stimulated Dbp5. Second, the N-terminal 53 residues of DDX19 that precede the auto-inhibitory helix have a subtle but distinct role, possibly similar to the role of the N-terminal residues in yeast Dbp5. Lastly, the weak Gle1-mediated stimulation of the hyperactive variants in the presence of RNA suggests that relief from auto-inhibition is indeed a major mechanism for Gle1-mediated stimulation.

### Human Nup214 is stimulatory in the presence of Gle1

We next analyzed the effect of the nucleoporin and mRNA export factor Nup214 on DDX19 activation in the context of Gle1^CTD^•Nup42^GBM^ stimulation. Nup214^NTD^ binds to DDX19^NTD^ via an interface that partially overlaps with the RNA binding interface, and consequently, binding of Nup214^NTD^ to DDX19^NTD^ impairs RNA binding and RNA-mediated stimulation of DDX19 ATPase activity^14,15^. In yeast, Nup214^NTD^ enhances ADP release from Dbp5 but the consequence of this effect is unclear, as Gle1^CTD^ stimulates Dbp5 to the same activity levels in the presence and absence of Nup214^NTD^^10,44^.

In the human system, addition of Nup214^NTD^ did not affect basal DDX19 ATPase activity or activity in the presence of Gle1^CTD^•Nup42^GBM^ (Fig. 7b). Consistent with previous reports, Nup214^NTD^ inhibited RNA-mediated stimulation (Fig. 7b)^14,15^. However, when DDX19 was stimulated by both Gle1^CTD^•Nup42^GBM^ and RNA, the addition of Nup214^NTD^ further increased ATPase activity (Fig. 7b). This effect was concentration dependent and more pronounced when Gle1^CTD^•Nup42^GBM^ was present in substoichiometric amounts (Fig. 7c). Thus, unlike what was found for the yeast system, Nup214^NTD^ can have a stimulatory effect in the context of Gle1-mediated DDX19 stimulation^10^. We next utilized an electrophoretic mobility shift assay to probe the effects of Gle1^CTD^•Nup42^GBM^ and Nup214^NTD^ on RNA binding by DDX19. Whereas Gle1^CTD^•Nup42^GBM^ enhanced DDX19 binding to RNA, Nup214^NTD^ inhibited DDX19 binding to a 53-nucleotide single-stranded RNA (Fig. 7d). However, Gle1^CTD^•Nup42^GBM^ rescued RNA binding in the presence of Nup214^NTD^, consistent with its ability to rescue DDX19 from Nup214^NTD^ inhibition (Fig. 7b, d). Thus, Nup214 not only recruits DDX19 to the cytoplasmic face of the NPC, but can also play an active role in promoting DDX19 ATPase activity (See Supplementary Note 7).

### Human disease mutations in Gle1

Several human diseases have been linked to Gle1 dysfunction, including ALS, Huntington’s disease, and the related disorders LCCS1 and LAAHD, which lead to spinal cord motor neuron atrophy and premature death, often prior to birth^30,33,34^. For ALS and LCCS1/LAAHD, specific mutations have been mapped to Gle1^CTD^. In ALS, three mutations have been associated with Gle1: a nonsense mutation that results in a truncated protein after residue 70, a splice site mutation that replaces the C-terminal 44 residues of Gle1^CTD^ with a novel 88-residue sequence, and a missense mutation leading to the amino acid substitution R697C^34^. LCCS1 and LAAHD are related disorders, in which the more phenotypically severe LCCS1 patients are typically homozygous for a 3-residue insertion at position 144 in the coiled-coil region of Gle1 (Fin_Major_) and the less phenotypically severe LAAHD patients are compound heterozygous for the Fin_Major_ mutation and a mutation in Gle1^CTD^ (V617M and I684T)^33^. The R569H variant in Gle1^CTD^ was identified in a patient diagnosed with the more severe LCCS1, suggesting it has a more potent effect than the V617M and I684T variants.

When mapped onto the structure of Gle1^CTD^•Nup42^GBM^•DDX19^∆N53^, none of the four amino acid substitutions directly participated in binding to Nup42 or DDX19 (Fig. 7e; Supplementary Fig. 19a−d). We next evaluated the Gle1^CTD^•Nup42^GBM^ variants for thermostability and ability to stimulate DDX19. All three LCCS1/LAAHD mutations possessed altered thermostability that correlated with their phenotypic disease manifestation, with the R569H variant exhibiting the largest difference in *T*_m_, from 50 °C for wild-type protein to 40 °C for the disease variant (Fig. 7f). In contrast, there were no substantial defects in ATPase stimulation for any of the disease variants (Fig. 7g). In our crystal structures, R569 is involved in an extensive network of intra-molecular interactions (Supplementary Fig. 19a), whereas V617 and I684 are both buried in hydrophobic cores (Supplementary Fig. 19b, c), supporting the conclusion that these mutations reduce the stability of Gle1 rather than affecting their ability to stimulate the activity of DDX19. Future work will need to address whether Gle1 stability is also a contributing factor in human disease.

## Discussion

Using an interdisciplinary approach, we have garnered insight into the mRNA export events that occur at the cytoplasmic face of the NPC. Gle1^CTD^ plays an important organizational role by providing spatially separated binding sites for Nup42^GBM^ and the DEAD-box helicase DDX19/scDbp5. Strikingly, the Gle1^CTD^•Nup42^GBM^ interaction mechanism is virtually identical in species separated by more than a billion years of evolution. A comprehensive structure-function analysis of DDX19 indicates that binding of Gle1^CTD^•Nup42^GBM^ induces a conformational change in DDX19, thereby stimulating its ATPase activity. Biochemical analyses of Gle1 variants associated with human disease suggest that nucleoporin stability is a contributing factor in motor neuron disease.

While this paper was in revision, another group reported that both Nup42 and IP_6_ directly affect Gle1-mediated DDX19 stimulation^45^. Informed by our studies of Gle1^CTD^ thermostability, we attribute the observed stimulation by Nup42^GBM^ to its role in increasing Gle1^CTD^ stability, rather than a direct role in activation. In the yeast system, we also observed apparent stimulation by Nup42^GBM^ at 37 °C, which is the temperature used in the other study^46^. However, our thermostability experiments indicate that at 37 °C, Gle1^CTD^ solubility is compromised (Fig. 2b). In contrast, stimulation by Nup42^GBM^ at 30 °C was largely undetectable (Supplementary Fig. 13c). This conclusion is further supported by examination of the crystal structures, which revealed that Nup42^GBM^ binds Gle1^CTD^ on the opposite face as the scDbp5/DDX19-binding interfaces. Furthermore, we did not detect any stimulatory effect of Nup42^GBM^ in the human system.

In addition, we found that unlike yeast Dbp5, human DDX19 activation by Gle1^CTD^ does not require IP_6_. This conclusion was supported by multiple lines of evidence: (1) robust and indistinguishable ATPase stimulation of human DDX19 by Gle1^CTD^ both in the presence and absence of IP_6_, (2) human Gle1^CTD^ and DDX19 formed a complex in SEC experiments in the absence of IP_6_, (3) no binding of IP_6_ to human Gle1^CTD^•Nup42^GBM^ was detected in ITC experiments, (4) crystal structures uncover that the human Gle1 pocket corresponding to the yeast Gle1 IP_6_ binding pocket possesses a dramatically reduced positive electrostatic surface potential, (5) IP_6_ coordinating residues in yeast Dbp5 were not conserved in human DDX19, and (6) no electron density for IP_6_ was observed in crystal structures of human Gle1^CTD^•Nup42^GBM^•DDX19^∆N53^ determined using crystals soaked or co-crystallized with IP_6_. We note that our studies were all performed with extensively purified proteins recombinantly expressed in bacteria (Supplementary Fig. 13a, b) whereas DDX19 was purified from an insect cell expression system in the other study^45^. Future work needs to assess whether other proteins, small molecules, or post-translational modifications play a role in regulating DDX19 activity.

**Fig. 8:**
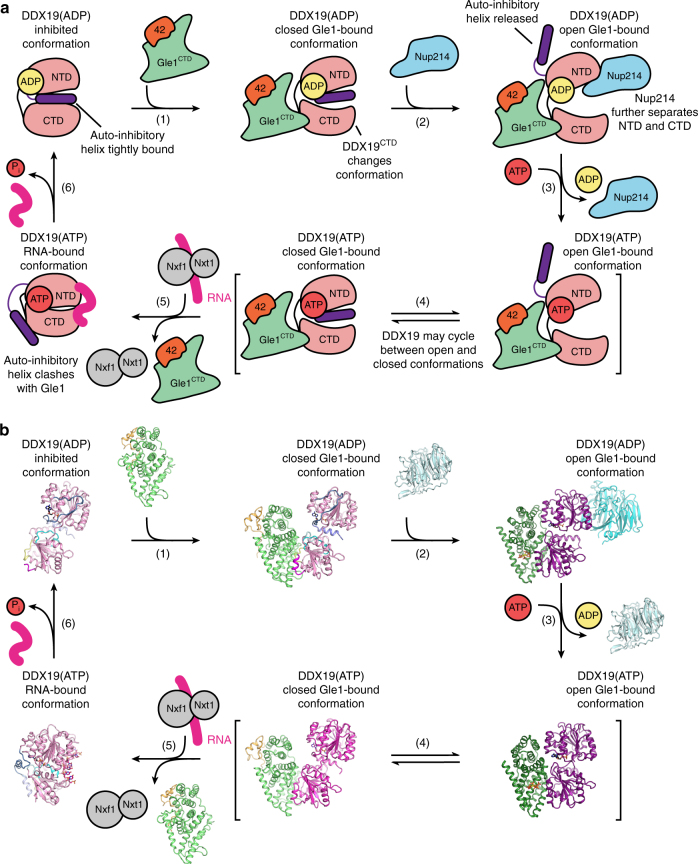
Proposed working model for the DDX19 catalytic cycle. **a** Schematic cartoon of the DDX19 catalytic cycle. **b** Schematic of the DDX19 catalytic cycle with crystal structures of each state. The inhibited conformation corresponds to the crystal structure of DDX19^∆N53^(ADP) (PDB ID 3EWS)^44^. The closed Gle1-bound conformations correspond to the crystal structures of Gle1^CTD^•Nup42^GBM^•DDX19^∆N53^(AMP-PNP•Mg^2+^) and Gle1^CTD^•Nup42^GBM^•DDX19^∆N53^(ADP). The Gle1-bound open conformations correspond to the yeast Gle1^CTD^•IP_6_•Dbp5^∆N90^(ADP) and Gle1^CTD^•IP_6_•Dbp5^∆N90^(ADP)•Nup159^NTD^ (PDB IDs 3RRM and 3RRN)^10^. The RNA bound conformation corresponds to the crystal structure of DDX19^∆N53^(AMP-PNP•Mg^2+^)•U_10_ RNA (PDB IDs 3RRM and PDB ID 3G0H)^44^. Gle1^CTD^•Nup42^GBM^ and Nup214^NTD^ correspond to the *apo* structures (PDB ID 2OIT)^69^

Combining these results, we can propose a working model of the DDX19 catalytic cycle (Fig. 8; Supplementary Movie 3). DDX19 is predominantly trapped in the auto-inhibited state, which can form in the presence of either ATP or ADP, although the ATP- and ADP-bound states could exhibit different dynamics for release of the auto-inhibitory helix. Gle1 binding facilitates release from auto-inhibition by first inducing conformational rearrangements in DDX19^CTD^ that partially open DDX19 and generate a cleft between DDX19^NTD^ and DDX19^CTD^ (Fig. 8, step 1). Nup214^NTD^ binding can accelerate the opening of the domains leading to complete separation of the DDX19^NTD^ and DDX19^CTD^, forming the conformation observed in the structure of *S*. *cerevisiae* Gle1^CTD^•IP_6_•Dbp5^∆N90^ (Fig. 8, step 2). Separation of DDX19^NTD^ and DDX19^CTD^ opens the nucleotide-binding pocket and enables exchange of ADP for ATP (Fig. 8, step 3). The auto-inhibitory helix could rebind and DDX19 would cycle between open and closed conformations until it encounters RNA (Fig. 8, step 4). RNA binding favors the formation of the closed, catalytically active state and based on crystallographic data, would also trigger release of Gle1 due to rearrangements in DDX19. Specifically, the auto-inhibitory helix displaces DDX19’s C-terminal helix and the mobile loop connecting the auto-inhibitory helix resulting in a conformation that is incompatible with Gle1 binding (Fig. 8, step 5). This process leads to displacement of Nxf1•Nxt1, although future studies are necessary to establish how DDX19 specifically removes Nxf1•Nxt1. After ATP hydrolysis and subsequent dissociation of *P*_i_ and RNA, DDX19 would then recycle back to the inhibited state and another cycle of ATP hydrolysis could then occur (Fig. 8, step 6).

There remains a substantial gap between rates of ATP hydrolysis measured in vitro (~1 ATP/second) and rates of mRNA export termination observed in vivo (<0.1 s per mRNA)^46,47^. A portion of this gap could be explained by a specific spatial organization at the cytoplasmic face of the NPC that efficiently accelerates the DDX19 cycle. Mapping of the Gle1-binding site on Nup155 provides a critical spatial restraint for the location of Gle1 and the entire cytoplasmic filament subcomplex in the NPC. Attempts to dock the newly determined structures of the human Gle1^CTD^•Nup42^GBM^•DDX19^∆N53^ into the cryoelectron tomographic reconstruction of the intact human NPC did not identify any unambiguous placements, although the rapid binding kinetics of DDX19 at the NPC suggest that it may not be present in the reconstructions (Supplementary Fig. 20)^48,49^. Furthermore, all of the cytoplasmic filament proteins studied here contain additional domains that may play important roles in DDX19 activity regulation. However, a complete characterization of these effects is expected to require the reconstitution of a fully assembled cytoplasmic filament nucleoporin complex, including the remaining domains of Nup42, Nup214, Gle1, their FG repeats, and their binding partners. This complex will need to be assembled onto the human CNC rings that serve as a scaffold in the intact NPC and assayed with Nxf1•Nxt1-bound mRNAs. The results presented here provide a rich foundation for the design of these experiments.

Lastly, the effect of Nup42 on Gle1 stability highlights protein instability as a potent modifier of Gle1 function, as the Gle1^CTD^ fold is unstable at physiological temperatures in vitro in the absence of its cofactors. The effects of mutations associated with human disease in our thermostability experiments largely correlated with their phenotypic severity, suggesting that protein stability may be a contributing factor in these diseases. Interestingly, measurements of *S. cerevisiae* Gle1^CTD^•IP_6_ thermostability in the absence of Nup42^GBM^ in vitro matched the temperatures at which we observed mislocalization of Gle1 in yeast cells, presumably a result of misfolding and subsequent dissociation from the NPC (Fig. 2a). A similar phenomenon occurs in human cells, as the mutations that destabilize human Gle1^CTD^ have previously been found to possess NPC localization defects^49^. Overall, our results reinforce the link between mislocalization or aggregation of proteins involved in RNA metabolism and nucleocytoplasmic transport with human disease. Future studies that build upon the results reported here to determine the contribution of protein destabilization in disease models should provide greater insight into the mechanisms underlying motor neuron disease.

## Methods

### Chemicals and reagents

Chemicals were purchased from Sigma at the highest purity available unless otherwise noted.

### Construct generation

DNA fragments were amplified using the polymerase chain reaction. SUMO-tagged proteins were cloned into a modified pET28a vector^50^. GST-tagged proteins were cloned into a pGEX-6P-1 vector. His_6_-tagged proteins were cloned into a modified pET28a vector^51^. *S. cerevisiae* and *H. sapiens* Gle1^CTD^•Nup42^GBM^ were cloned into a modified pETDuet-1 vector with Nup42^GBM^ in the first site and Gle1^CTD^ in the second site. Mutants were generated using the QuikChange mutagenesis protocol and confirmed by DNA sequencing. Details of bacterial expression constructs and expression conditions are shown in Supplementary Table 5.

### Protein expression and purification

Proteins were expressed in *E. coli* BL21-CodonPlus(DE3)-RIL cells (Stratagene) in Luria-Bertani media and induced at an OD_600_ of ~0.6 with 0.5 mM IPTG (Gold Biotechnology). Unless indicated otherwise, cells were harvested by centrifugation and resuspended in a buffer containing 20 mM TRIS (pH 8.0), 500 mM NaCl, 4 mM 2-mercaptoethanol (β-ME), and 15 mM imidazole, supplemented with complete EDTA-free protease inhibitor cocktail (Roche) and flash frozen in liquid nitrogen. Cells were supplemented with 1 mg deoxyribonuclease I (Roche), lysed with a cell disruptor (Avestin), and centrifuged at 4 °C and 30,000 × *g* for 1 h. Supernatants were loaded onto an Ni-NTA affinity column (GE Healthcare) equilibrated in a buffer containing 20 mM TRIS (pH 8.0), 500 mM NaCl, 4 mM β-ME, and 15 mM imidazole and eluted with a linear gradient of imidazole concentration to 500 mM. Eluted proteins were dialyzed overnight with a buffer containing 20 mM TRIS (pH 8.0), 100 mM NaCl, 4 mM β-ME, and 15 mM imidazole and subsequently purified through affinity, ion exchange, and SEC.

hsGle1^CTD^•His_6_-hsNup42^GBM^ variants, scGle1^CTD^•His_6_-scNup42^GBM^, and His_6_-scGle1^CTD^ were grown for 18 h at 18 °C. His_6_-ctGle1^CTD^ was co-expressed with GST-ctNup42^GBM^ and grown for 3 h at 37 °C. After elution from the Ni-NTA column, the His_6_ tag and the GST tag were removed by cleavage with PreScission protease concurrent with dialysis. Dialyzed protein was loaded onto an Ni-NTA column equilibrated in a buffer containing 20 mM TRIS (pH 8.0), 100 mM NaCl, 4 mM β-ME, and 15 mM imidazole. Protein-containing fractions were pooled and loaded onto a HiTrap Heparin HP column (GE Healthcare) equilibrated in a buffer containing 20 mM TRIS (pH 8.0), 100 mM NaCl, and 5 mM dithiothreitol (DTT) and eluted with a linear gradient of NaCl concentration to 2 M. Protein-containing fractions were concentrated and loaded onto a HiLoad Superdex 75 16/60 PG column (GE Healthcare) equilibrated in a buffer containing 20 mM TRIS (pH 8.0), 100 mM NaCl, and 5 mM DTT.

His_6_-SUMO-hsGle1^CTD^ was grown for 18 h at 18 °C and purified as hsGle1^CTD^•His_6_-hsNup42^GBM^, except the His_6_-SUMO tag was removed by Ulp1 cleavage.

His_6_-hsDDX19 variants, His_6_-scDbp5 and His_6_-SUMO-ctDbp5 were grown for 18 h at 18 °C. Cells were harvested by centrifugation and resuspended in a buffer containing 20 mM TRIS (pH 8.0), 500 mM NaCl, 4 mM β-ME, 15 mM imidazole, and 5% (v/v) glycerol, supplemented with Complete EDTA-free protease inhibitor cocktail (Roche) and flash frozen in liquid nitrogen. After lysis and centrifugation, the supernatant was loaded onto an Ni-NTA column equilibrated in a buffer containing 20 mM TRIS (pH 8.0), 500 mM NaCl, 4 mM β-ME, 15 mM imidazole, and 5% (v/v) glycerol. Protein was eluted with a linear gradient of imidazole concentration to 500 mM and protein-containing fractions were dialyzed overnight in a buffer containing 20 mM TRIS (pH 8.0), 100 mM NaCl, 4 mM β-ME, 15 mM imidazole, and 5% (v/v) glycerol. The His_6_ tags of His_6_-DDX19 variants and His_6_-scDbp5 were cleaved using PreScission protease concurrent with overnight dialysis. The His_6_-SUMO tag of His_6_-SUMO-ctDbp5 was cleaved with Ulp1 protease and the cleaved protein was desalted with dialysis buffer immediately after elution from the Ni-NTA column. The dialyzed/desalted protein was loaded onto an Ni-NTA column equilibrated in a buffer containing 20 mM TRIS (pH 8.0), 100 mM NaCl, 4 mM β-ME, 15 mM imidazole, and 5% (v/v) glycerol. Protein-containing flowthrough fractions were loaded onto a HiTrap Q HP column (GE Healthcare) equilibrated in a buffer containing 20 mM TRIS (pH 8.0), 100 mM NaCl, 5 mM DTT, and 5% (v/v) glycerol and eluted with a linear gradient of NaCl concentration to 2 M. Protein-containing fractions were concentrated and loaded onto a HiLoad Superdex 200 16/60 PG column (GE Healthcare) equilibrated in a buffer containing 20 mM TRIS (pH 8.0), 100 mM NaCl, 5 mM DTT, and 5% (v/v) glycerol.

His_6_-SUMO-hsGle1^N^ and variants were grown at 30 °C for 2 h after IPTG induction. After elution from the Ni-NTA column, proteins were dialyzed overnight in a buffer containing 20 mM TRIS (pH 8.0), 100 mM NaCl, and 5 mM DTT. After dialysis, proteins were loaded onto a HiTrap Q HP column and eluted with a linear gradient of NaCl concentration to 2 M. Protein-containing fractions were concentrated and loaded onto a Superdex 200 10/300 GL column (GE Healthcare) equilibrated in a buffer containing 20 mM TRIS (pH 8.0), 100 mM NaCl, and 5 mM DTT.

His_6_-SUMO-scNup42^GBM^-His_6_ and variants were grown at 37 °C for 2 h after IPTG induction. After elution from the Ni-NTA column, the His_6_-SUMO tag was removed by cleavage with Ulp1 concurrent with overnight dialysis into a buffer containing 20 mM TRIS (pH 8.0), 100 mM NaCl, and 5 mM DTT. After dialysis, proteins were injected onto a HiTrap Q HP column and collected in the flowthrough. Protein-containing fractions were concentrated and loaded onto a Superdex Peptide 10/300 GL column (GE Healthcare).

His_6_-Nup155^CTD^ and His_6_-Nup214^NTD^ were grown for 18 h at 18 °C after IPTG induction. After elution from the Ni-NTA column, proteins were dialyzed overnight in 20 mM TRIS (pH 8.0), 500 mM NaCl, 4 mM β-ME, and 15 mM imidazole. His_6_ tags were removed by cleavage with PreScission protease concurrent with dialysis. Dialyzed protein was loaded onto an Ni-NTA column equilibrated in a buffer containing 20 mM TRIS (pH 8.0), 100 mM NaCl, 4 mM β-ME, and 15 mM imidazole. Protein-containing fractions were pooled and loaded onto a HiTrap Q HP column equilibrated in a buffer containing 20 mM TRIS (pH 8.0), 100 mM NaCl, and 5 mM DTT and eluted with a linear gradient of NaCl concentration to 2 M. Protein-containing fractions were concentrated and loaded onto a HiLoad Superdex 200 16/60 PG column equilibrated in a buffer containing 20 mM TRIS (pH 8.0), 100 mM NaCl, and 5 mM DTT.

His_6_-SUMO-Nup98^∆FG^ was purified as Nup155^CTD^ and Nup214^NTD^, except the His_6_-SUMO tag was removed by cleavage with Ulp1.

GST-Nup155^CTD^ was grown for 18 h at 18 °C after IPTG induction. After harvesting, lysis, and centrifugation, supernatant was loaded onto a Glutathione Sepharose 4 Fast Flow column (GE Healthcare) equilibrated in a buffer containing 20 mM TRIS (pH 8.0), 150 mM NaCl, and 5 mM DTT. GST-Nup155^CTD^ was eluted with a linear gradient of reduced glutathione concentration to 10 mM. Protein-containing fractions were loaded onto a HiTrap Q HP column equilibrated in a buffer containing 20 mM TRIS (pH 8.0), 100 mM NaCl, and 5 mM DTT and eluted with a linear gradient of NaCl concentration to 2 M. Protein-containing fractions were concentrated and loaded onto a HiLoad Superdex 200 16/60 PG column equilibrated in a buffer containing 20 mM TRIS (pH 8.0), 100 mM NaCl, and 5 mM DTT.

### Crystallization and structure determination

*S. cerevisiae* Gle1^CTD^•Nup42^GBM^ was crystallized at 23 °C with the hanging drop method using 1 μl of protein solution (22.5 mg/ml) and 1 μl of reservoir solution, containing 0.1 M HEPES (pH 8.2), 11% (w/v) PEG 3350, and 0.2 M l-Proline. Crystals were cryoprotected with a solution identical to the reservoir solution, but supplemented with 30% (v/v) ethylene glycol. X-ray diffraction data were collected at beamline 12-2 at the Stanford Synchotron Radiation Lightsource (SSRL) with a wavelength of 0.9795 Å and processed with the XDS package^52^. The structure was solved by molecular replacement with Phaser, using the structure of Gle1^CTD^ (PDB ID 3RRN) as a search model^10,53^. The structure was refined using the PHENIX package with TLS refinement^54^. One of the Gle1 molecules in the asymmetric unit displays two distinct conformations for half the molecule and was modeled as two alternate conformations for the entire residue range. The final structure was refined to *R*_work_ and *R*_free_ values of 18.5 and 21.1%, respectively, with excellent geometry (98.3% of residues in favored region of the Ramachandran plot, 1.7% in the allowed region, and 0.0% outliers; MolProbity clashscore 1.92; MolProbity score 0.96)^55^.

*H. sapiens* Gle1^CTD^•Nup42^GBM^ was crystallized at 23 °C with the hanging drop method using 1 μl of protein solution (20 mg/ml) and 1 μl of reservoir solution, containing 0.2 M sodium potassium phosphate (pH 7.6) and 26% (w/v) PEG 3350. Crystals were cryoprotected by gradual supplementation of ethylene glycol in 5% steps to a final concentration of 30% (v/v). X-ray diffraction data were collected at beamline 23-ID-D at the Advanced Photon Source (APS) with a wavelength of 0.9794 Å and processed with the XDS package^52^. The diffraction data were anisotropic, with diffraction limits along the three principal components of 2.8, 3.1, and 3.1 Å. The structure was solved by molecular replacement with Phaser, using the structure of *S. cerevisiae* Gle1^CTD^ (PDB ID 3RRN) as a search model^10,53^. Refinement was performed using torsion NCS restraints with the PHENIX package using anisotropically truncated and scaled data generated using the UCLA-DOE anisotropy server^54,56^. The final structure was refined to *R*_work_ and *R*_free_ values of 24.5 and 27.4%, respectively, with excellent geometry (95.2% of residues in favored region of the Ramachandran plot, 4.8% in the allowed region, and 0.0% outliers; MolProbity clashscore 1.88; MolProbity score 1.31)^55^.

*C. thermophilum* Gle1^CTD^•Nup42^GBM^ was crystallized at 23 °C with the hanging drop method using 1 μl of protein solution (10 mg/ml) and 1 μl of reservoir solution, containing 0.1 M MES (pH 6.3) and 12% (w/v) PEG 20,000. Crystals were cryoprotected by gradual supplementation of ethylene glycol in 5% steps to a final concentration of 25% (v/v). X-ray diffraction data were collected at beamline 23-ID-D at APS with a wavelength of 1.0332 Å and processed with the XDS package^52^. The structure was solved by molecular replacement with Phaser, using the structure of *H. sapiens* Gle1^CTD^ as a search model^53^. The structure was refined with torsion NCS restraints using the PHENIX package^54^. The final structure was refined to *R*_work_ and *R*_free_ values of 24.0 and 27.7%, respectively, with excellent geometry (97.5% of residues in favored region of the Ramachandran plot, 2.5% in the allowed region, and 0.0% outliers; MolProbity clashscore 3.34; MolProbity score 1.22)^55^.

*C. thermophilum* Gle1^CTD^•Nup42^GBM^•IP_6_ was crystallized at 23 °C with the hanging drop method using 1 μl of protein solution (7.5 mg/ml) and 1 μl of reservoir solution, containing 10 mM zinc sulfate heptahydrate, 0.1 M MES (pH 6.3), and 18% (v/v) Polyethylene glycol monomethyl ether 550. Crystals were improved by microseeding. Crystals were cryoprotected by gradual supplementation of ethylene glycol in 5% steps to a final concentration of 25% (v/v). SeMet-labeled crystals were grown using the same conditions. X-ray diffraction data were collected at beamline 12-2 at SSRL with a wavelength of 0.9792 Å for SeMet crystals and 1.0332 Å for native crystals, and processed with the XDS package^52^. The structure was solved using Crank2, using the structure of *H. sapiens* Gle1^CTD^ as an initial search model^57,58^. The structure was refined using the PHENIX package^54^. The presence of Zn^2+^ ions was confirmed using anomalous difference Fourier maps. The final structure was refined to *R*_work_ and *R*_free_ values of 19.3 and 23.0%, respectively, with excellent geometry (98.3% of residues in favored region of the Ramachandran plot, 1.7% in the allowed region, and 0.0% outliers; MolProbity clashscore 0.56; MolProbity score 0.69)^55^.

For crystallization of the *H. sapiens* Gle1^CTD^•Nup42^GBM^•DDX19^∆N53^, the complex was reconstituted by mixing equimolar amounts of purified Gle1^CTD^•Nup42^GBM^ and DDX19^∆N53^ to form a 400 μM stock solution. The stock solution was supplemented with 400 μM ADP or AMP-PNP and 400 μM MgCl_2_. Protein stock solutions were diluted with a buffer containing 20 mM TRIS (pH 8.0), 100 mM NaCl, 5 mM DTT, 1 mM MgCl_2_, and 1 mM ADP or AMP-PNP. Crystals of Gle1^CTD^•Nup42^GBM^•DDX19^∆N53^(ADP) were grown at 4 °C with the hanging drop method using 1 μl of protein solution (7.5 mg/ml) and 1 μl of reservoir solution, containing 13% (w/v) PEG 3350 and 0.2 M sodium potassium phosphate. Crystals of Gle1^CTD^•Nup42^GBM^•DDX19^∆N53^(AMP-PNP•Mg^2+^) were grown at 4 °C with the hanging drop method using 1 μl of protein solution (5 mg/ml) and 1 μl of reservoir solution in 15% (w/v) PEG 3350 and 0.3 M sodium potassium phosphate (pH 6.6). Crystals were cryoprotected by gradual supplementation of ethylene glycol to a final concentration of 25% (v/v) in 5% (v/v) steps.

Diffraction data for Gle1^CTD^•Nup42^GBM^•DDX19^∆N53^(ADP) were collected at beamline 23-ID-D at APS with a wavelength of 1.0333 Å and processed with XDS^52^. The diffraction data were anisotropic, with diffraction limits along the three principal components of 3.6, 3.7, and 4.0 Å. The crystal structure was solved by molecular replacement with Phaser, using the structure of *H. sapiens* Gle1^CTD^•Nup42^GBM^ and *H. sapiens* DDX19 (PDB ID 3EWS) as search models^43^. Refinement was performed using torsion NCS restraints with the PHENIX package using anisotropically truncated and scaled data generated using the UCLA-DOE anisotropy server^54,56^. The final structure was refined to *R*_work_ and *R*_free_ values of 23.2 and 28.1%, respectively, with excellent geometry (95.9% of residues in favored region of the Ramachandran plot, 4.1% in the allowed region, and 0.0% outliers; MolProbity clashscore 3.86; MolProbity score 1.45)^55^.

Diffraction data for Gle1^CTD^•Nup42^GBM^•DDX19^∆N53^(AMP-PNP•Mg^2+^) were collected at the Frontier Microfocusing Macromolecular Crystallography beamline (FMX) at the National Synchotron Lightsource-II (NSLS-II) with a wavelength of 0.9793 Å. The crystals grew in space group P2_1_ but were non-merohedrally twinned with the twin domains related by a 180° rotation along *a*. Diffraction data were integrated with DIALS and scaled with AIMLESS^59,60^. The structure was solved by molecular replacement with Phaser, using the structures of *H. sapiens* Gle1^CTD^•Nup42^GBM^ and *H. sapiens* DDX19 (PDB ID 3EWS) as search models^43,53^. The diffraction data were anisotropic, with diffraction limits along the three principal components of 3.4, 3.7, and 4.2 Å. Refinement was performed with PHENIX using anisotropically truncated and scaled data generated with the UCLA-DOE anisotropy server^54,56^. The final structure was refined to *R*_work_ and *R*_free_ values of 26.1 and 31.3%, respectively, with excellent geometry (95.0% of residues in favored region of the Ramachandran plot, 5.0% in the allowed region, and 0.0% outliers; MolProbity clashscore 2.72; MolProbity score 1.43)^55^.

For the structures of Gle1^CTD^•Nup42^GBM^•DDX19^∆N53^(AMP-PNP•Mg^2+^) grown in the presence of or soaked with IP_6_, crystals of Gle1^CTD^•Nup42^GBM^•DDX19^∆N53^(AMP-PNP•Mg^2+^) were grown in a drop additionally supplemented with 0.5 mM IP_6_ or soaked for 4 days with 0.5 mM IP_6_, respectively. Crystals grown or soaked with IP_6_ were cryoprotected with solutions supplemented with 0.5 mM IP_6_. Diffraction data were collected at beamline 23-ID-D at APS, integrated with DIALS and scaled with AIMLESS^59,60^. The structures were solved by direct substitution of the coordinates of the structure of Gle1^CTD^•Nup42^GBM^•DDX19^∆N53^(AMP-PNP•Mg^2+^) and refined with the PHENIX package^54^. The structure of Gle1^CTD^•Nup42^GBM^•DDX19^∆N53^(AMP-PNP•Mg^2+^) soaked with IP_6_ was refined to *R*_work_ and *R*_free_ values of 24.6 and 29.7%, respectively, with a MolProbity score of 1.38^55^. The structure of Gle1^CTD^•Nup42^GBM^•DDX19^∆N53^(AMP-PNP•Mg^2+^) co-crystallized with IP_6_ was refined to *R*_work_ and *R*_free_ values of 25.8 and 31.3%, respectively, with a MolProbity score of 1.41^55^. Simulated annealing omit maps were calculated using the PHENIX package by omitting all ligand molecules^54^.

Crystals of DDX19^∆N53^(AMP-PNP•Mg^2+^) grew during crystallization trials of Gle1^CTD^•Nup42^GBM^•DDX19^∆N53^(AMP-PNP•Mg^2+^) in heavy precipitate. The reservoir solution contained 0.1 M malonate, imidazole, borate (MIB) buffer (pH 5.0) and 13% (w/v) PEG 1500. Crystals were cryoprotected with the reservoir solution supplemented with 20% (v/v) ethylene glycol. X-ray diffraction data were collected at beamline 23-ID-D at APS with a wavelength of 1.0333 Å and the data were integrated with DIALS and scaled with AIMLESS^59,60^. The crystal structure was solved by molecular replacement using the structure of DDX19^∆N53^(ADP) (PDB ID 3EWS)^43^. The structure was refined with TLS groups using the PHENIX package^54^. The final structure was refined to *R*_work_ and *R*_free_ values of 20.7 and 24.8%, respectively, with excellent geometry (98.9% of residues in favored region of the Ramachandran plot, 1.1% in the allowed region, and 0.0% outliers; MolProbity clashscore 2.04; MolProbity score 0.97)^55^.

For details of the data collection and refinement statistics for all structures, see Supplementary Tables 1–4. For representative views of electron density for all structures, see Supplementary Figure 21.

### Analytical size-exclusion chromatography

Protein−protein interaction experiments were carried out on a Superdex 200 10/300 GL or Superdex 75 10/300 GL gel filtration column equilibrated in a buffer containing 20 mM TRIS (pH 8.0), 100 mM NaCl, and 5 mM DTT (when present, 0.5 mM IP_6_ was added to the buffer). The different combinations were mixed and incubated for 30 min on ice using a twofold molar excess of the smaller component. Complex formation was evaluated by comparing the mobility on the gel filtration column of pre-incubated proteins versus individual proteins. Complex formation was confirmed by SDS-PAGE of the protein-containing fractions, followed by Coomassie brilliant blue staining.

### GST pull-down interaction analysis

Glutathione-S-transferase (GST) pull-down experiments were performed with purified GST-Nup155^CTD^, Nup98^ΔFG^, and SUMO-Gle1^N^. 25 μl of Glutathione Sepharose 4 Fast Flow beads (GE Healthcare) were equilibrated with a buffer containing 20 mM TRIS (pH 8.0), 100 mM NaCl and 5 mM DTT. GST-Nup155^CTD^ (20 μM) was preincubated for 30 min with a twofold molar ratio of either Nup98^ΔFG^ or SUMO-Gle1^N^ on ice. Preincubated mixtures were subsequently incubated with 0, 0.5, 1, 1.5, 2, 4, or 8-fold molar ratios of the other protein for another 30 min on ice. Mixtures were then incubated with pre-equilibrated glutathione-coupled sepharose beads for 30 min and washed five times with a buffer containing 20 mM TRIS (pH 8.0), 100 mM NaCl and 5 mM DTT and centrifuged at 500 × *g* at 4 °C. Bound protein was eluted from the beads with 25 μl buffer containing 20 mM TRIS (pH 8.0), 100 mM NaCl, 5 mM DTT, and 10 mM reduced glutathione.

### Yeast strain generation

The *nup42∆/gle1-GFP* strain was generated in a haploid BY4741 parental strain by first introducing the *natNT2* cassette by homologous recombination into the *nup42* gene, followed by three rounds of selection on yeast extract peptone dextrose (YPD) plates containing Nourseothricin (Gold Biotechnology)^61^. Subsequently, a GFP-*kanMX* cassette was inserted into the C-terminus of Gle1 followed by three rounds of selection on YPD plates containing G418^61^. Nup42-mCherry-3xHA variants were introduced using a modified pRS415 plasmid followed by two rounds of selection on plates containing leucine-depleted synthetic dextrose complete medium (SDC-LEU). For details of yeast strains and constructs, see Supplementary Table 6.

### Yeast live cell fluorescence

Cells were grown in SDC-LEU medium to mid-log phase at 30 °C and shifted to 42 °C for 3 h. For fluorescence imaging, cells were pelleted by centrifugation for 2 min at 650 × *g*, resuspended in water, and imaged using a Carl Zeiss Observer Z.1 equipped with a Hamamatsu camera C10600 Orca-R2.

### Yeast growth assay

For growth analysis, cells were grown in SDC-LEU medium at 30 °C to an OD_600_ of 0.2. Fifteen microliters of a tenfold dilution series was spotted onto plates containing SDC-LEU medium, which were incubated at 30 and 37 °C.

### Western blot analysis

The expression levels of Nup42-mCherry-3xHA variants were assessed for the *S. cerevisiae nup42Δ/gle1-GFP* strain. Transformed cells were selected twice on SDC-LEU plates before analysis. Protein extraction from cells was performed via NaOH and SDS treatment, using a modified protocol^62^. Specifically, cells were grown at 30 °C to an OD_600_ of ∼1.0 before harvesting 10 ml of culture by centrifugation for 5 min at 3000 × *g*. Cell pellets were resuspended in 100 μl of 0.3 M NaOH and incubated for 5 min on ice, pelleted by centrifugation at 3000 × *g*, washed with water twice, and resuspended in a buffer containing 20 mM TRIS (pH 8.0), 250 mM NaCl and 2% (w/v) SDS, incubated at 80 °C for 10 min followed by centrifugation at 30,000 × *g* for 4 min. The supernatant was collected, diluted with SDS loading dye, and boiled for 3 min prior to loading on a SDS-PAGE gel. Western blot analysis of Nup42-mCherry-3xHA variants was performed with a 1 h incubation at room temperature with mouse anti-HA antibody (Covance; MMS-101P; 1:5000 dilution) and a 1 h incubation at room temperature with an anti-mouse antibody coupled to alkaline phosphatase (Promega; S3721; 1:5000 dilution) visualized by color development using SIGMAFAST 5-bromo-4-chloro-3-indolyl phosphate (BCIP)/nitro blue tetrazolium (NBT) tablets (Sigma; B5655). Equal loading was established with an overnight incubation at 4 °C with rabbit anti-hexokinase antibody (US Biological; H2035-02; 1:10,000 dilution) and 1 h incubation at room temperature with a goat anti-rabbit antibody fused to an IR800 fluorescent probe (Li-Cor, 926-32211; 1:10,000 dilution) imaged with an IR imager (Li-Cor Odyssey) using the 800 nm channels in a single scan at 169 μm resolution and a scan intensity of 5. Antibodies were diluted in TBS-T supplemented with 3% (w/v) milk powder and washes were carried out in TBS-T.

### Differential scanning fluorimetry assay

Differential scanning fluorimetry was performed using a previously described protocol^63^. Using a real-time PCR instrument (Bio-Rad C1000 96-well Thermal Cycler), fluorescence of a 20 μl mixture of 5 μM purified protein and 5× SYPRO orange dye (Invitrogen) was measured once per minute while the temperature was increased 1 °C/min from 4 to 95 °C. Reactions were performed in 20 mM TRIS (pH 8.0), 100 mM NaCl, and 5 mM DTT. When present, IP_6_ was supplemented to 25 μM for yeast proteins and 100 μM for human proteins. The reported *T*_m_ values were determined by finding the temperature corresponding to the maximum of the first derivative of the thermal melting curve. Traces represent the average of three experiments.

### Pelleting thermostability assay

Fifty-microliter samples of purified protein (10 μg each) were incubated for 30 min at indicated temperatures between 25 and 55 °C. Soluble and pellet fractions were isolated by centrifugation at 30,000 × g for 35 min at 4 °C. Reactions were performed in a buffer containing 20 mM TRIS (pH 8.0), 100 mM NaCl, and 5 mM DTT. When present, IP_6_ was added to a final concentration of 20 μM. Proteins were resolved by SDS-PAGE gel and visualized with Coomassie brilliant blue staining.

### NADH-coupled ATPase assay

Steady-state ATPase activity rates were determined at 30 and 37 °C for scDbp5 and hsDDX19 using previously established conditions^41^. The reaction mixture (80 μl) contained purified scDbp5 and hsDDX19 (WT and variants) at 0.5 and 2.5 μM, respectively. Unless otherwise noted, all other *S. cerevisiae* and *H. sapiens* proteins (Gle1^CTD^, Gle1^CTD^•Nup42^GBM^, or Nup214^NTD^) were present at concentrations of 1 and 5 μM, respectively. When present, polyA RNA with length ranging between 465 and 660 bases (GE Healthcare, 27411001) was added to a final concentration of 0.1 mg/ml. When present, IP_6_ was supplemented to a final concentration of 2 and 10 μM for yeast and human, respectively, unless otherwise noted. The reaction mixture contained 30 mM HEPES (pH 7.5), 100 mM NaCl, 2 mM MgCl_2_, 1 mM DTT, 6 mM phosphoenolpyruvate (PEP, Alfa Aesar), 1.2 mM NADH, 1.6 μl pyruvate kinase (PK)/lactate dehydrogenase enzyme solution (LDH), and 2.5 mM ATP, unless otherwise noted.

For each reaction, two separate mixtures were prepared and kept separate until initiation of the reaction. The first mixture, containing the protein components, IP_6_, polyA RNA, and buffer (HEPES, NaCl, and MgCl_2_), was prepared to a final volume of 20 μl and incubated on ice. The second mixture, containing buffer, DTT, PEP, NADH, ATP, and PK/LDH, was prepared to a final volume of 60 μl and dispensed into a 96-well plate. Reactions were initiated by addition of the protein mixture. Plates were centrifuged at 4000 × *g* for 2 min at 4 °C prior to being loaded in the pre-warmed plate reader. A_340_ was measured every 30 s for 30 min using a FlexStation 3 microplate reader (Molecular Devices). Rates were calculated by fitting the linear portion of the reaction. Reported rates were determined by dividing the rate of ATP consumption by the concentration of DDX19/Dbp5 in the reaction. All values reported are the average of three experiments.

### Isothermal titration calorimetry experiments

ITC measurements were performed at 21 °C using a MicroCal ITC200 Calorimeter (GE Healthcare/Malvern) in a buffer containing 100 mM HEPES (pH 7.4), 100 mM NaCl, 2 mM MgCl_2_, and 4 mM β-ME. Protein solutions were dialyzed overnight against ITC buffer and IP_6_ was dissolved in the dialysate. All measurements were performed in triplicate. Because of the acidic nature of IP_6_, the heat generated from dilution was subtracted for baseline correction. Baseline corrected data were analyzed with Origin 7.0 software. For *S*. *cerevisiae* Gle1^CTD^•Nup42^GBM^, cell and syringe concentrations of 5 and 50 μM were used for Gle1^CTD^•Nup42^GBM^ and IP_6_, respectively. For human Gle1^CTD^•Nup42^GBM^, measurements were made at identical conditions to *S*. *cerevisiae* Gle1^CTD^•Nup42^GBM^, as well as at cell and syringe concentrations of 200 μM and 2 mM for Gle1^CTD^•Nup42^GBM^ and IP_6_, respectively.

### Electrophoretic mobility shift assay

The electrophoretic mobility of free or DDX19-bound RNA was evaluated on native 1.4% (w/v) agarose gels using a 53-nucleotide single-stranded RNA probe (5′-GUUUU UUUUU UUUUU UUUUU UCUCG AUCCG UAGUG AUCUA CUGAG CAUCU CCC-3′). The RNA was prepared by in vitro transcription using T7 polymerase and according to the MEGAscript protocol (Ambion). The transcribed RNA was loaded on a 8% denaturing polyacrylamide-urea gel (19:1 acryl:bisacryl ratio and 8.3 M urea). The RNA band was cut, eluted, and extracted with phenol:chloroform, followed by ethanol precipitation. The pellet was resuspended in 10 mM TRIS (pH 8.5) buffer and the concentration was detected by measuring the absorbance at 260 nm. The RNA-protein reactions were carried out in 20 mM TRIS (pH 8.0), 100 mM NaCl, 2 mM MgCl_2_, 1 mM DTT, 10% (v/v) glycerol, and 1 mM AMP-PNP. The recombinant proteins used in each assay were incubated in binding buffer containing AMP-PNP and then mixed with 25 ng of RNA. The final reaction volume was 10 μl and the final protein concentrations were 1 μM Gle1^CTD^•Nup42^GBM^, 2 μM Nup214^NTD^, and 1 μM DDX19. After mixing, samples were incubated on ice for 20 min before being loaded onto a 1.4% (w/v) native agarose gel using 0.25× TRIS borate EDTA (TBE) buffer. Electrophoresis was carried out at a constant voltage of 9 V/cm at room temperature in 0.25× TBE buffer for 40 min. The gel was stained with SYBR gold (1:10,000 dilution in 0.25× TBE buffer) for 3 min and imaged.

### Crystal structure docking

Searches using the structures of Gle1^CTD^•Nup42^GBM^•DDX19^∆N53^(AMP-PNP•Mg^2+^) or Gle1^CTD^•Nup42^GBM^ were performed in a previously reported cryo-electron tomography reconstruction of the intact human NPC (EMD-3103) using the Chimera FitMap tool^48,64^. Searches in unaccounted cytoplasmic density were performed in a map generated by removing density in the composite structure of the symmetric core of the NPC^2^. Searches were performed with 50,000 initial placements within 100 Å of the cytoplasmic outer ring that were locally optimized and scored based on correlation to a 25 Å simulated map of the structures.

### Illustrations and figures

All structural figures and movies were ray traced in PyMol (Schrödinger). Surface electrostatic potential plots were calculated using Adaptive Poisson-Boltzmann Solver (APBS)^65^. Secondary structure predictions were generated with the PSIPRED server^66^. Sequence alignments were generated using MUSCLE and visualized with ALSCRIPT^67,68^.

### Data availability

Atomic coordinates and related structure factors have been deposited in the Protein Data Bank with accession codes 6B4E, 6B4F, 6B4G, 6B4H, 6B4I, 6B4J, and 6B4K for the structures of *S. cerevisiae* Gle1^CTD^•Nup42^GBM^, *H. sapiens* Gle1^CTD^•Nup42^GBM^, *C. thermophilum* Gle1^CTD^•Nup42^GBM^, *C. thermophilum* Gle1^CTD^•Nup42^GBM^•IP_6_, *H. sapiens* Gle1^CTD^•Nup42^GBM^•DDX19^∆N53^(ADP), *H. sapiens* Gle1^CTD^•Nup42^GBM^•DDX19^∆N53^(AMP•PNP), and *H. sapiens* DDX19^∆N53^(AMP•PNP), respectively. Other data are available from the corresponding author upon reasonable request.

## Electronic supplementary material

Supplementary Information

Description of Additional Supplementary Files

Supplementary Movie 1

Supplementary Movie 2

Supplementary Movie 3
