# Supplementary material for: Structural and functional analysis of mRNA export regulation by the nuclear pore complex
Source: Nat Commun. 2018 Jun 13;9:2319. doi: 10.1038/s41467-018-04459-3 (PMC5998080; doi:10.1038/s41467-018-04459-3)
Supplement: Supplementary file 1 — Supplementary Information [file 41467_2018_4459_MOESM1_ESM.pdf]

Supplementary Information for

Structural and functional analysis of mRNA export regulation by the nuclear pore complex

Daniel H. Lin, Ana R. Correia,[1] Sarah W. Cai,[1] Ferdinand M. Huber, Claudia A. Jette, André Hoelz*

Affiliation:

California Institute of Technology, Division of Chemistry and Chemical Engineering, 1200 East California Boulevard, Pasadena, CA, 91125, USA

[1]These authors contributed equally

*Correspondence: hoelz@caltech.edu (A.H.)

Supplementary Notes 1-7

Supplementary Figures 1-21

Supplementary Tables 1-6

Supplementary References

Supplementary Note 1: Identification of a minimal Nup42 fragment sufficient for Gle1 binding. We utilized a haploid *nup42Δ S. cerevisiae* strain harboring a genomic C-terminal Gle1-GFP fusion analogous to a previously reported strain. This *nup42Δ/gle1-GFP* strain displays a temperature-sensitive phenotype causing mislocalization of Gle1 from the nuclear rim under heat shock conditions[1]. We monitored the localization of Gle1-GFP and mCherry-HA-tagged Nup42 variants: full-length Nup42 (residues 1-430), Nup42 lacking the entire N-terminal FG repeat region (residues 364-430), two further N-terminally truncated variants (residues 397-430 and 410-430), and Nup42 containing the FG repeats but harboring a 36-residue C-terminal truncation (residues 1-394). Consistent with previous reports, these strains were viable, and the deletion of Nup42 did not affect Gle1 localization at 30 °C (Fig. 2a; Supplementary Fig. 3a,b)[1]. Nup42 truncations that contained residues 397-430 displayed nuclear rim staining consistent with localization to the NPC, whereas a Nup42 variant containing only residues 410-430 did not (Fig. 2a). Strains harboring mislocalized Nup42 variants also displayed a temperature-sensitive growth phenotype at 37 °C along with a loss of Gle1-GFP from the nuclear rim following a 42 °C heat shock (Fig. 2a; Supplementary Fig. 3b). Furthermore, these data suggested that the $Nup42^{GBM}$-Gle1 interaction was not only required for Nup42 localization to the NPC, but also for maintenance of Gle1 localization at the NPC during heat shock. We note that the fluorescent protein fusions contributed to the growth phenotype; the Nup42-dependent phenotype at 37 °C was only observed for strains harboring a genomic C-terminal Gle1-GFP fusion, in agreement with previous reports that Nup42 is not required in wild-type yeast at 37 °C[2]. Moreover, given the artificial nature of the *nup42Δ/gle1-GFP* strain and the primarily unstructured Nup42 N-terminal region, we cannot exclude that other regions on Nup42 may contain additional binding sites for other nucleoporins.

Supplementary Note 2: Details of interactions between Nup42 and Gle1. The recognition of Gle1 by Nup42 is primarily mediated by a conserved core set of hydrophobic interactions supplemented by additional peripheral interactions. Specifically, the *S. cerevisiae* Nup42 hydrophobic core contains two proline residues (P420 and P424) that point their pyrrolidine rings inwards towards phenylalanine residues F409 and F414 (Fig. 2h). This core wraps around and buries the exposed hydrophobic Gle1 residues W451, Y488, and L495 (Fig. 2h). This hydrophobic interface is supplemented by polar interactions, including a salt bridge between Nup42 residue D421 and Gle1 residue R456, as well as a network of hydrogen bonds between Gle1 residues Q491 and K494

with the backbone carbonyls of Nup42 residues I408, F409, A411, and L428 (Fig. 2h). Furthermore, these interactions are highly conserved: human Gle1 residues W602, Y637, M644 are recognized by human Nup42 residues F401, F406, P412, and P416; *C. thermophilum* Gle1 residues W447, Y484, and A491 are recognized by *C. thermophilum* Nup42 residues W530, F539, P544, and P548. *C. thermophilum* $Nup42^{GBM}$ is extended by 24 additional N-terminal residues which increase the interaction surface area (Fig. 2f; Supplementary Fig. 11a). This insertion appeared to be present at the sequence level among species within the *Pezizomycotina* subphylum of fungi (Supplementary Fig. 9).

Supplementary Note 3: *C. thermophilum* IP_6-binding pocket. To directly assess the conformational changes in $Gle1^{CTD}$ induced by IP_6 binding independent of Dbp5 binding, we compared our *apo* and IP_6-bound *C. thermophilum* $Gle1^{CTD}$•$Nup42^{GBM}$ structures (Supplementary Fig. 7a). Minimal conformational changes occur upon IP_6 binding, mostly limited to a loop directly adjacent to the IP_6 pocket (Supplementary Fig. 7a). In the *C. thermophilum* $Gle1^{CTD}$•$Nup42^{GBM}$•IP_6 structure, two IP_6 molecules coordinating two Zn^{2+} ions from the crystallization buffer bind simultaneously to each $Gle1^{CTD}$. Multiple IP_6 molecules were also observed in structures of *S. cerevisiae* $Gle1^{CTD}$ determined in the presence of IP_6 (without divalent cations) and the IP_6 primary binding site overlaps directly for the two species (Supplementary Fig. 7c)[3]. Thus, despite differences in the location of positively charged residues in the *S. cerevisiae* and *C. thermophilum* IP_6 pockets, the electrostatic potential of the pocket was conserved and IP_6 bound in a similar orientation, suggesting that IP_6 could function in a similar role in *C. thermophilum* as in *S. cerevisiae* (Fig. 3a,b,d,e; Supplementary Fig. 7d).

Supplementary Note 4: Differences between human and yeast DDX19/Dbp5 complexes. Our sequence conservation analysis indicated that the IP_6-binding lysine residues in the C-terminal α-helix of *S. cerevisiae* Dbp5 are not conserved in human DDX19 (Supplementary Fig. 12b). Instead, the DDX19 C-terminal α-helix is two helical turns shorter and packs more closely to $Gle1^{CTD}$ to position D470, D472, and E475 to form salt bridges in the second interface (Fig. 5f). Another difference in human $Gle1^{CTD}$ is that helix $\alpha2$ curves to allow K416 and K419 to form salt bridges with $DDX19^{CTD}$ and other residues to form more contacts with $DDX19^{CTD}$. Additionally, a 12-residue β-tongue insertion between helices $\alpha3$ and $\alpha4$, unique to human $Gle1^{CTD}$, also forms new contacts

with DDX19 (Fig. 5f). Altogether, the human proteins utilize a more extensive interface to compensate for the absence of IP_6 binding.

Supplementary Note 5: Conformational changes in DDX19 induced by Gle1 binding. First, the C-terminal helix became ordered, positioning the acidic residues of the helix to interact with lysines on the $Gle1^{CTD}$ surface, resulting in the formation of interface 2 (Fig. 5c,6a; Supplementary Movie 2). In our structure of *apo* $DDX19^{\Delta N53}$(AMP-PNP•Mg^{2+}), the C-terminal helix is already partially ordered, likely because of crystal contacts (Supplementary Fig. 16). The second difference was a conformational rearrangement in the adjacent "trigger loop" (residues 328-335), allowing I331 to pack against the C-terminal helix, T332 to pack against Gle1, and moving Q335 out of the way of the neighboring "anchor loop" (residues 390-403) (Fig. 6b; Supplementary Movie 2). This rearrangement was reinforced by the extensive hydrogen bond network formed with $Gle1^{CTD}$ residues, resulting in the formation of interface 1 (Figs. 5b,6b; Supplementary Movie 2). Third, there was a large rearrangement of the DDX19 anchor loop that led to a shift in register (see residue C393) and movement away from the auto-inhibitory helix (Fig. 6c; Supplementary Movie 2). This loop rearrangement removes several contacts between the auto-inhibitory helix and $DDX19^{CTD}$, including a salt bridge between D398 and R67 and several hydrophobic interactions (Fig. 6c), which provides an explanation for the partial separation of $DDX19^{NTD}$ and $DDX19^{CTD}$. Lastly, the loop containing DEAD-box motif VI (residues 429-435), which was disordered in both the *apo* $DDX19^{\Delta N53}$(ADP) and $DDX19^{\Delta N53}$(AMP-PNP•Mg^{2+}) structures, became ordered. Motif VI contains several residues that directly bind ATP in the closed, active conformation (R429, R432, and F433), and they adopt very similar conformations to those observed in the structures of $DDX19^{\Delta N53}$(AMP-PNP•Mg^{2+})•RNA (Fig. 6d; Supplementary Movie 2). Importantly, the $Gle1^{CTD}$ residues involved in the $DDX19^{CTD}$-binding interfaces are also involved in stabilizing conformational changes in $DDX19^{CTD}$ through hydrogen bonds and salt bridges, explaining the sensitivity of DDX19 activation to the single point mutations we made in $Gle1^{CTD}$ (Fig. 5e). In summary, $Gle1^{CTD}$ binding to $DDX19^{CTD}$ causes a cascade of conformational changes that partially releases the auto-inhibitory helix and prepares the residues involved in nucleotide binding to form the closed, active conformation.

Supplementary Note 6: DDX19 variants. To obtain a better understanding of the role of various regulatory elements in the N-terminal region of human DDX19, we tested the activation of a series of truncation mutants that included DDX19$^{\Delta N53}$, the crystallized construct, which still contained the auto-inhibitory helix; DDX19$^{\Delta N67}$, which additionally removed the auto-inhibitory helix; and DDX19$^{\Delta N91}$, which further removed mobile residues that were a part of DDX19^{NTD} and was analogous to the previously used yeast Dbp5 truncation construct. Using our human DDX19 crystal structures, we also designed a mutant variant of full-length DDX19, DDX19$^{S60D/K64D}$, containing two aspartate substitutions in the auto-inhibitory helix that we predicted would disfavor formation of the inhibited state due to electrostatic repulsion.

Supplementary Note 7: How can Nup214^{NTD} binding be both inhibitory and stimulatory? We propose that Nup214^{NTD} binding also favors the separation of DDX19^{NTD} and DDX19^{CTD} due to steric clashes between Nup214^{NTD} loops and DDX19^{CTD} in the closed DDX19 conformations[4,5]. In previous structural work, we crystallized Nup214^{NTD} with full-length DDX19, but found only Nup214^{NTD} and DDX19^{NTD} to be ordered in the crystal structure due to the separation of the auto-inhibitory helix and DDX19^{CTD} from DDX19^{NTD} in that crystal[4]. Nup214^{NTD} also inhibited the activity of the hyperactive DDX19 mutants even in the absence of RNA (Supplementary Fig. 18), suggesting that Nup214^{NTD} binding to DDX19^{NTD} also prevents the formation of the closed, catalytically-competent conformation of DDX19. A similar role in separating yeast Dbp5^{NTD} and Dbp5^{CTD} was previously proposed for yeast Nup214^{NTD}, based on the crystal structure of yeast Gle1^{CTD}•IP_6•Dbp5$^{\Delta N90}$(ADP)•Nup214^{NTD}, in which the Dbp5^{NTD} is rotated further away from Dbp5^{CTD} than in the structure of yeast Gle1^{CTD}•IP_6•Dbp5$^{\Delta N90}$(ADP)[3]. Depending on which step is rate limiting, the separation of DEAD-box helicase domains may slow or enhance the DDX19 reaction rate. Notably, Gle1^{CTD} binding also inhibited the hyperactive DDX19 mutants, indicating that Gle1 binding also has multiple consequences on the DDX19 activity cycle (Fig. 7a). Further studies will be necessary to elucidate the precise molecular order of events in the DDX19 reaction cycle.

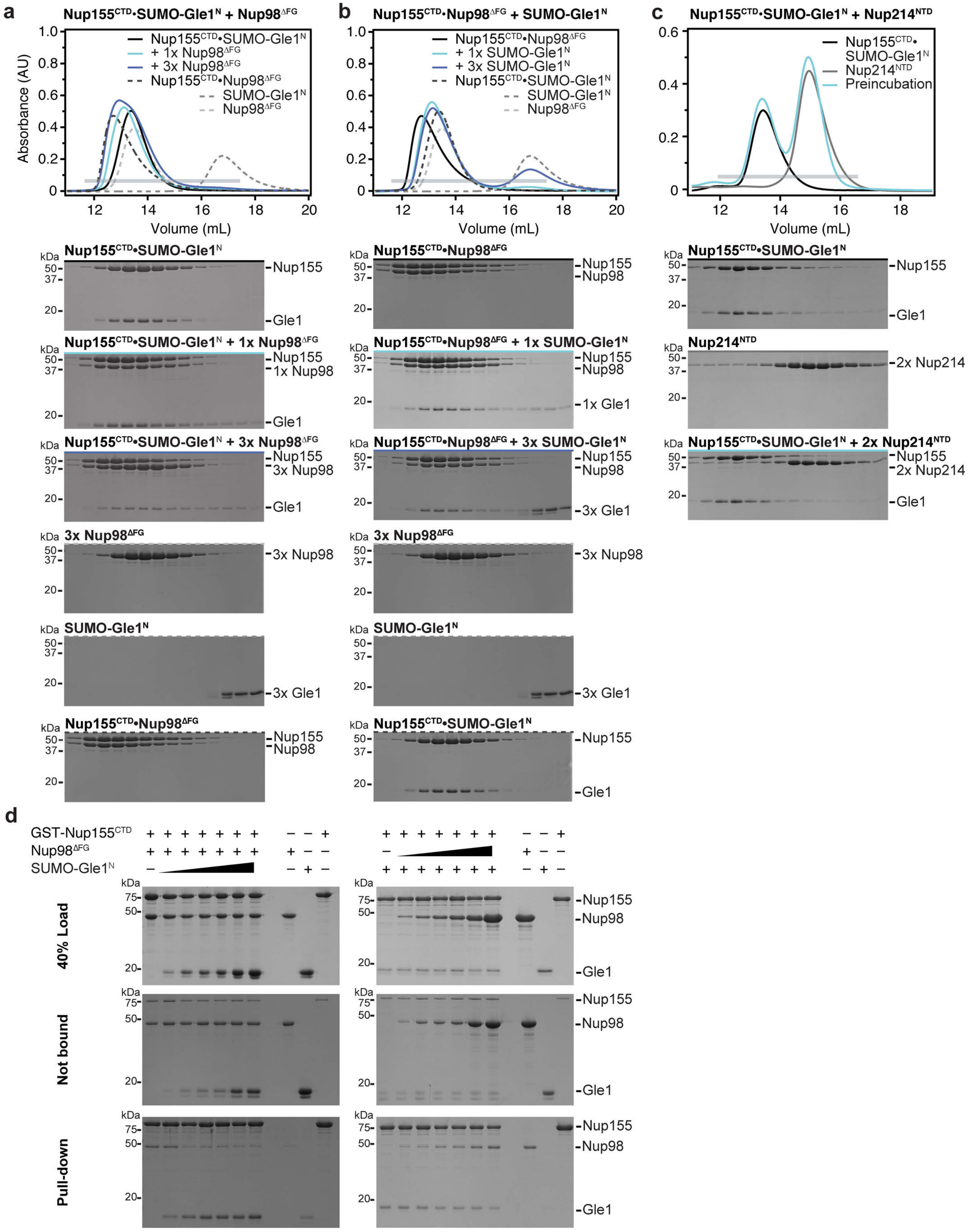

Supplementary Figure 1, Lin et al., 2018

Supplementary Figure 1: The interactions of Nup155^{CTD} with Nup98$^{\Delta FG}$ or Gle1^{N} are mutually exclusive. (a) Size exclusion chromatography analysis (SEC) of the interactions between Nup155^{CTD}•SUMO-Gle1^{N} and Nup98$^{\Delta FG}$ as in Figure 1c, but with additional control elution profiles. Purified Nup155•SUMO-Gle1^{N} complex was mixed with the indicated amounts of Nup98$^{\Delta FG}$ and loaded on a Superdex 200 10/300 GL column. The gray horizontal bar indicates the fractions visualized with Coomassie-stained SDS-PAGE gels. (b) SEC of the interactions between Nup155^{CTD}•Nup98$^{\Delta FG}$ and SUMO-Gle1^{N}. Purified Nup155•Nup98$^{\Delta FG}$ complex was mixed with the indicated amounts of SUMO-Gle1^{N} and loaded on a Superdex 200 10/300 GL column. Control elution profiles of Nup155•SUMO-Gle1^{N}, Nup98$^{\Delta FG}$ and SUMO-Gle1^{N} are included for reference. (c) Purified Nup155•SUMO-Gle1^{N} complex was mixed with 2-fold molar excess Nup214^{NTD} and loaded on a Superdex 200 10/300 GL column. No displacement is observed, demonstrating the specificity of the mutual exclusivity. (d) GST pull-down experiments with GST-Nup155^{CTD}. Complexes of (left) GST-Nup155^{CTD}•Nup98$^{\Delta FG}$ or (right) Nup155•SUMO-Gle1^{N} were preformed with a molar ratio of 1:2 and incubated for 30 minutes. After preincubation, the preformed complexes were incubated for 30 minutes with increasing concentrations of (left) SUMO-Gle1^{N} or (right) Nup98$^{\Delta FG}$ (molar ratios of 0.5, 1, 1.5, 2, 4, and 8). Samples were then pulled down with 25 µl of glutathione-coupled sepharose beads. Top gels contain the 40 % of the loaded sample, middle gels contain 40 % of the sample that was not pulled down, and bottom gels contain the glutathione-bound fractions.

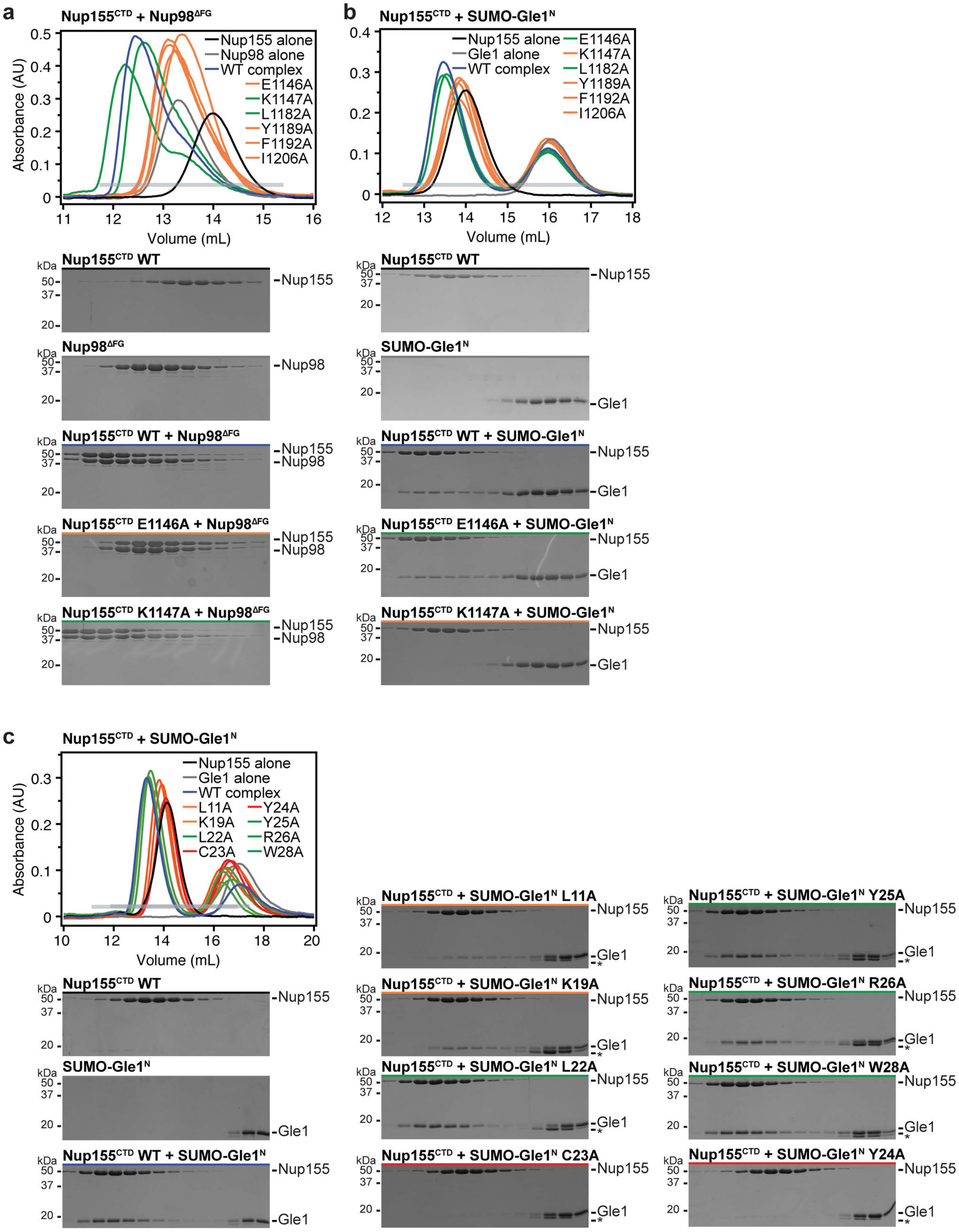

Supplementary Figure 2, Lin et al., 2018

Supplementary Figure 2: Identification of residues involved in the interactions of Nup98$^{\Delta FG}$ and Gle1^{N} with Nup155 binding. (a, b) SEC analysis of Nup155^{CTD} mutants for Nup98$^{\Delta FG}$ and SUMO-Gle1^{N} binding, respectively. Purified Nup155^{CTD} mutants were preincubated with (a) Nup98$^{\Delta FG}$ or (b) SUMO-Gle1^{N} and loaded on a Superdex 200 10/300 GL column. Control SEC profiles are shown for Nup155^{CTD} (black), SUMO-Gle1^{N} or Nup98$^{\Delta FG}$ (gray) and wild-type complex (blue). SEC profiles of Nup155^{CTD} mutants preincubated with either SUMO-Gle1^{N} or Nup98$^{\Delta FG}$ are colored green for wild-type levels of complex formation or orange for reduced binding. The gray horizontal bars in chromatograms indicate the fractions visualized with Coomassie-stained SDS-PAGE gels. (c) SEC analysis of the interactions between SUMO-Gle1^{N} alanine mutants and Nup155^{CTD}. Purified Nup155^{CTD} was mixed with the indicated SUMO-Gle1^{N} mutants and loaded on a Superdex 200 10/300 GL column. SEC profiles of Nup155^{CTD} (black), SUMO-Gle1^{N} (gray) and Nup155^{CTD} preincubated with SUMO-Gle1^{N} (blue) are shown as controls. SEC profiles of SUMO-Gle1^{N} alanine mutants preincubated with Nup155^{CTD} are colored green for wild-type levels of complex formation, orange for reduced binding, or red for complete disruption. Asterisks indicate degradation products of SUMO-Gle1^{N} variants.

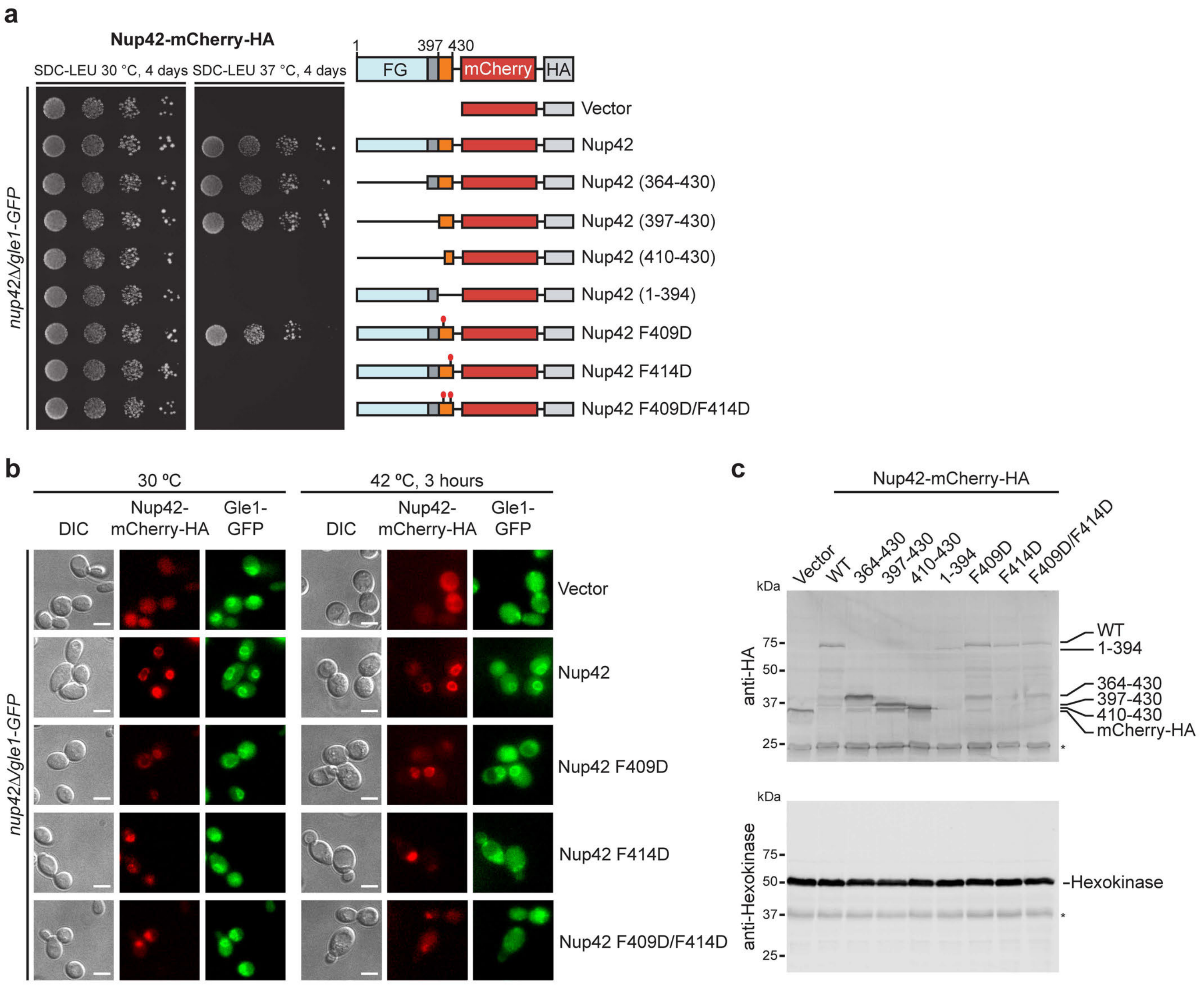

Supplementary Figure 3, Lin et al., 2018

Supplementary Figure 3: Analysis of interaction between Nup42^{GBM} and Gle1^{CTD} in *S. cerevisiae*. (a) Growth analysis of *S. cerevisiae nup42Δ/gle1-GFP* strains containing the indicated Nup42-mCherry-HA variants. 10-fold serial dilutions were spotted onto SDC-LEU plates and grown for 4 days at 30 °C and 37 °C. Schematics on the right indicate the domain boundaries of the Nup42 variants. A solid line indicates omitted regions. (b) *In vivo* localization analysis in *S. cerevisiae* of Gle1-GFP and Nup42-mCherry-HA variants. Constructs are the same as in panel a. Scale bar is 5 µm. (c) Western blot analysis of the expression levels of Nup42-mCherry-HA variants in a *S. cerevisiae nup42Δ/gle1-GFP* strain. Nup42-mCherry-HA variants and the hexokinase loading control were detected with anti-HA and anti-hexokinase antibodies, respectively. Asterisks (*) indicate nonspecific bands detected by the antibodies.

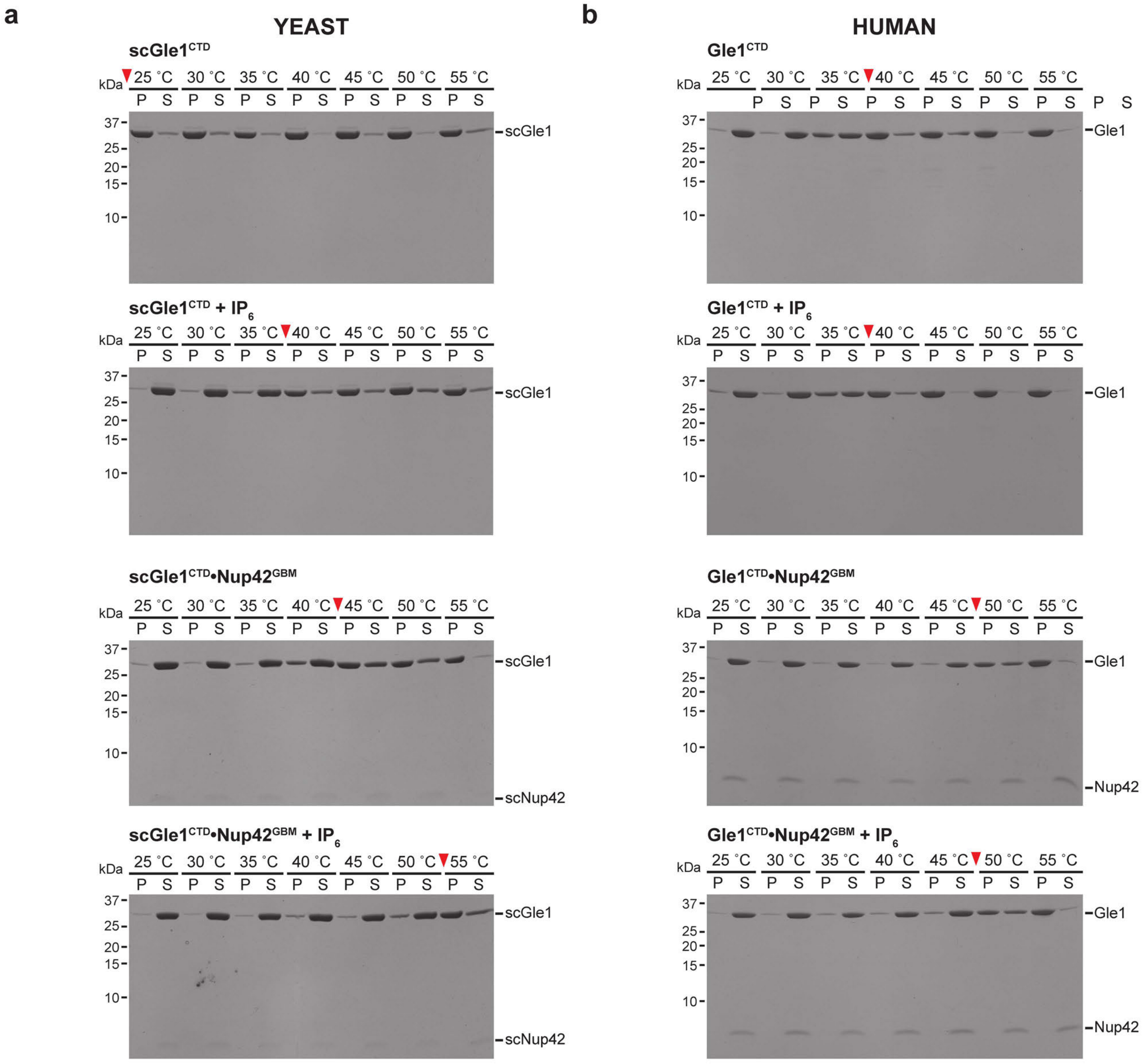

Supplementary Figure 4, Lin et al., 2018

Supplementary Figure 4: Pelleting thermostability assay. (a) *S. cerevisiae* $Gle1^{CTD}$ or $Gle1^{CTD}$•$Nup42^{GBM}$ was incubated at the indicated temperatures in the absence or presence of IP_6 for 30 minutes prior to centrifugation. (b) *H. sapiens* $Gle1^{CTD}$ or $Gle1^{CTD}$•$Nup42^{GBM}$ was incubated at the indicated temperatures in the absence or presence of IP_6 for 30 minutes prior to centrifugation. Pelleted (P) and soluble (S) fractions were analyzed by SDS-PAGE and visualized by Coomassie staining. Red arrows indicate the temperature at which more than 50 % of total $Gle1^{CTD}$ pelleted.

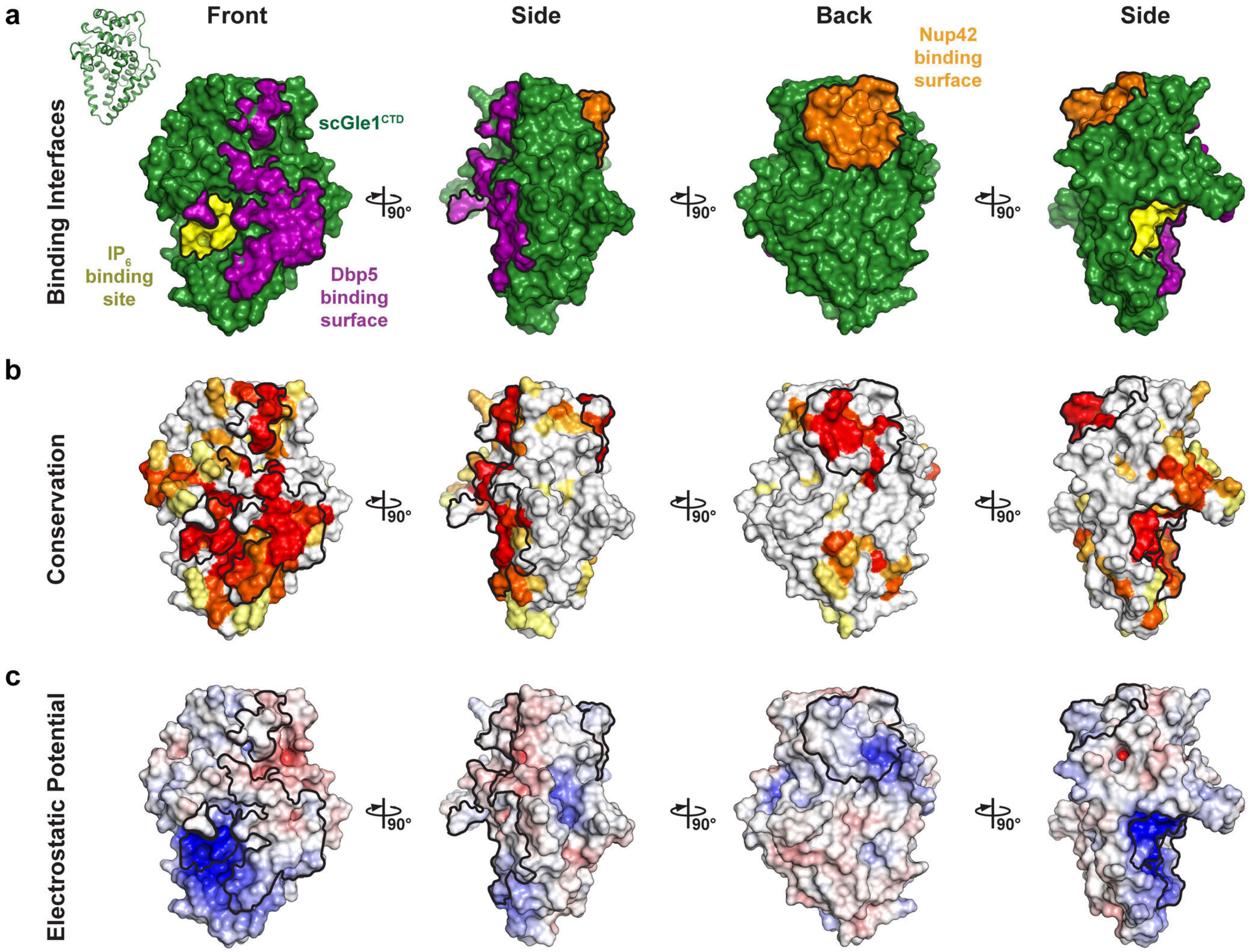

Supplementary Figure 5, Lin et al., 2018

Supplementary Figure 5: Surface properties of *S. cerevisiae* Gle1^{CTD}. Surface representations of Gle1^{CTD} in four orientations related by 90° rotations. The Nup42^{GBM}, IP_6, and Dbp5 binding interfaces are outlined in black. (a) Identification of *S. cerevisiae* Gle1^{CTD} binding surfaces. The IP_6 binding site is colored in yellow, Dbp5 binding interface is colored in purple, and Nup42^{GBM} interface is colored in orange. (b) Surface representation colored according to sequence conservation for fungi belonging to the Saccharomycotina and Schizosaccharomycetes groups using an alignment containing the species *S. cerevisiae*, *Z. rouxii*, *K. lactis*, *C. albicans*, *Y. liplytica, T. deformans, S. complicata,* and *S. pombe*. (c) Surface representation colored according to electrostatic potential from -10 k_BT/e (red) to 0 k_BT/e (white) to +10 k_BT/e (blue).

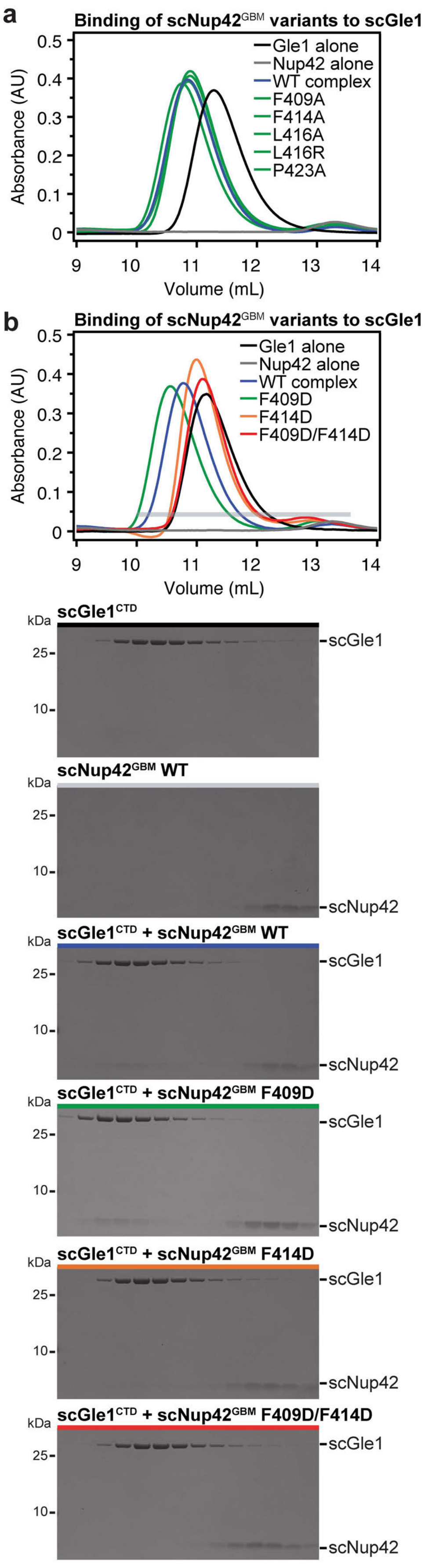

Supplementary Figure 6, Lin et al., 2018

Supplementary Figure 6: Analysis of interaction between scNup42^{GBM} and scGle1^{CTD}. (a,b) SEC analysis of the effect of mutations in scNup42^{GBM} on scGle1^{CTD} binding. Purified scGle1^{CTD} was mixed with the indicated scNup42^{GBM} mutants and loaded on a Superdex 75 10/300 GL column. SEC profiles of scGle1^{CTD} (black), scNup42^{GBM} (gray), and scGle1^{CTD} preincubated with scNup42^{GBM} (blue) are shown as controls. SEC profiles of scNup42^{GBM} mutants preincubated with scGle1^{CTD} are colored green for wild-type levels of complex formation, orange for reduced binding, or red for complete disruption. The gray horizontal bar indicates the fractions visualized with Coomassie-stained SDS-PAGE gels.

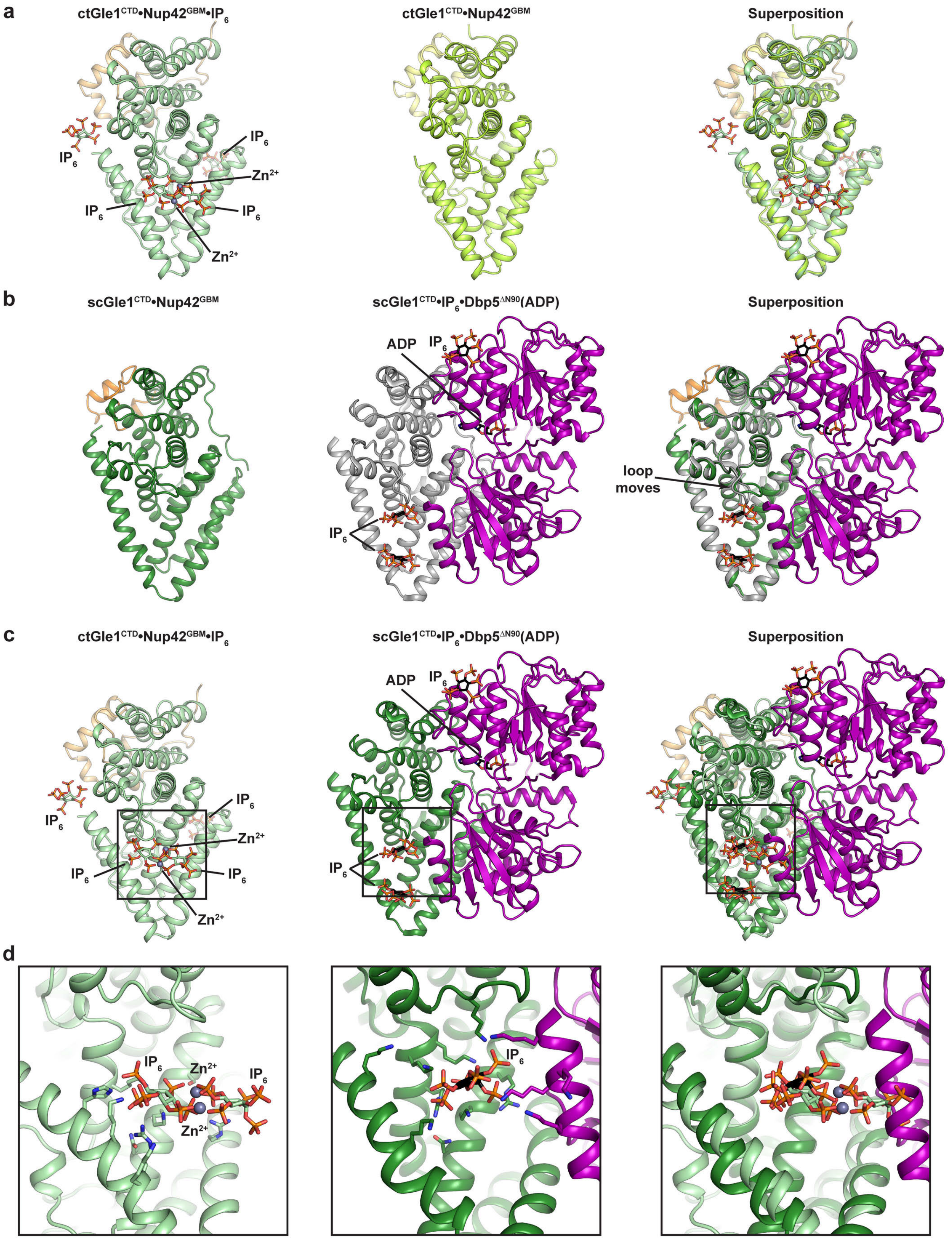

Supplementary Figure 7, Lin et al., 2018

Supplementary Figure 7: Analysis of the effect of IP_6 binding on $Gle1^{CTD}$ in fungi. (a) Comparison of the *C. thermophilum* $Gle1^{CTD}$•$Nup42^{GBM}$•IP_6 and $Gle1^{CTD}$•$Nup42^{GBM}$ structures. (b) Comparison of the *S. cerevisiae* $Gle1^{CTD}$•$Nup42^{GBM}$ and *S. cerevisiae* $Gle1^{CTD}$•IP6•$Dbp5^{\Delta N90}$(ADP) (PDB ID 3RRN) structures[3]. (c) Comparison of the *C. thermophilum* $Gle1^{CTD}$•$Nup42^{GBM}$•IP_6 and *S. cerevisiae* $Gle1^{CTD}$•IP_6•$Dbp5^{\Delta N90}$(ADP) structures (PDB ID 3RRN). (d) Zoomed view of the IP_6 binding pocket in the *C. thermophilum* $Gle1^{CTD}$•$Nup42^{GBM}$•IP_6 and *S. cerevisiae* $Gle1^{CTD}$•IP_6•$Dbp5^{\Delta N90}$(ADP) (PDB ID 3RRN) structures.

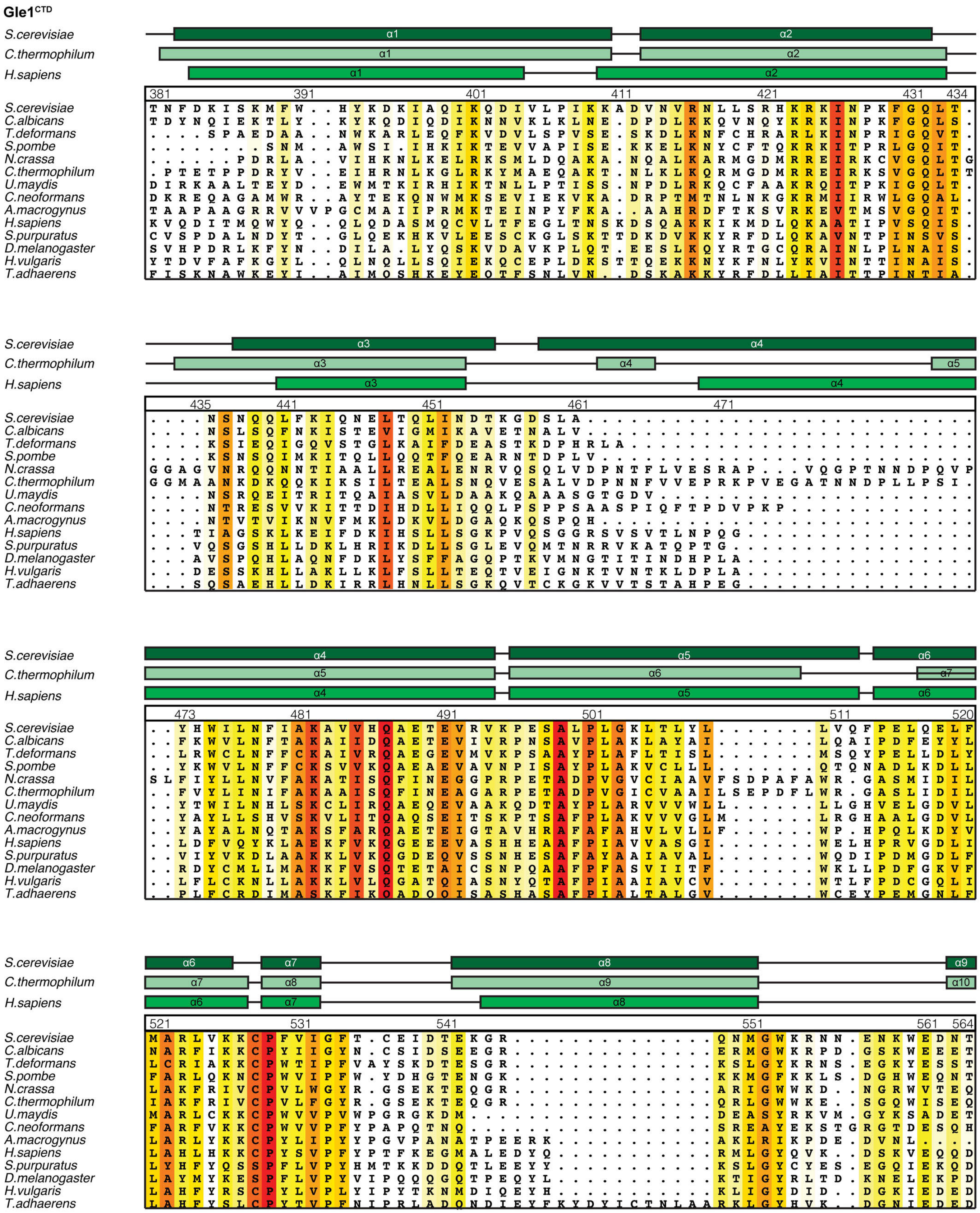

Supplementary Figure 8, Lin et al., 2018

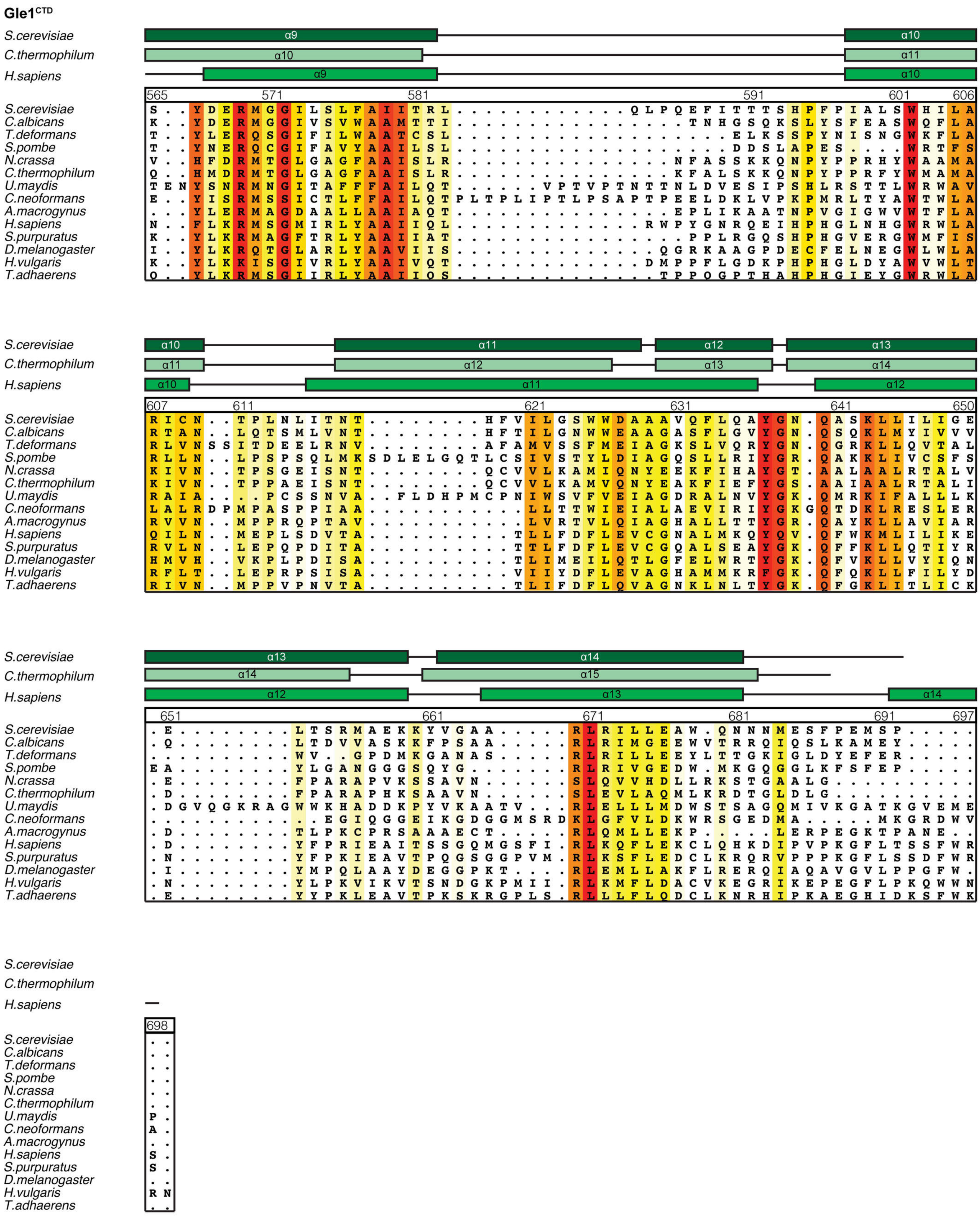

Supplementary Figure 8 continued, Lin et al., 2018

Supplementary Figure 8: Multispecies sequence alignment of Gle1^{CTD}. Sequences from fourteen diverse species were aligned and colored by sequence similarity according to the BLOSUM62 matrix from white (less than 40 % similarity), to yellow (55 % similarity), to red (100 % identity). The numbering is according to the *H. sapiens* protein. The secondary structure is indicated above the sequences as rectangles (α-helices) and lines (unstructured regions) for the *S. cerevisiae*, *C. thermophilum*, and *H. sapiens* proteins.

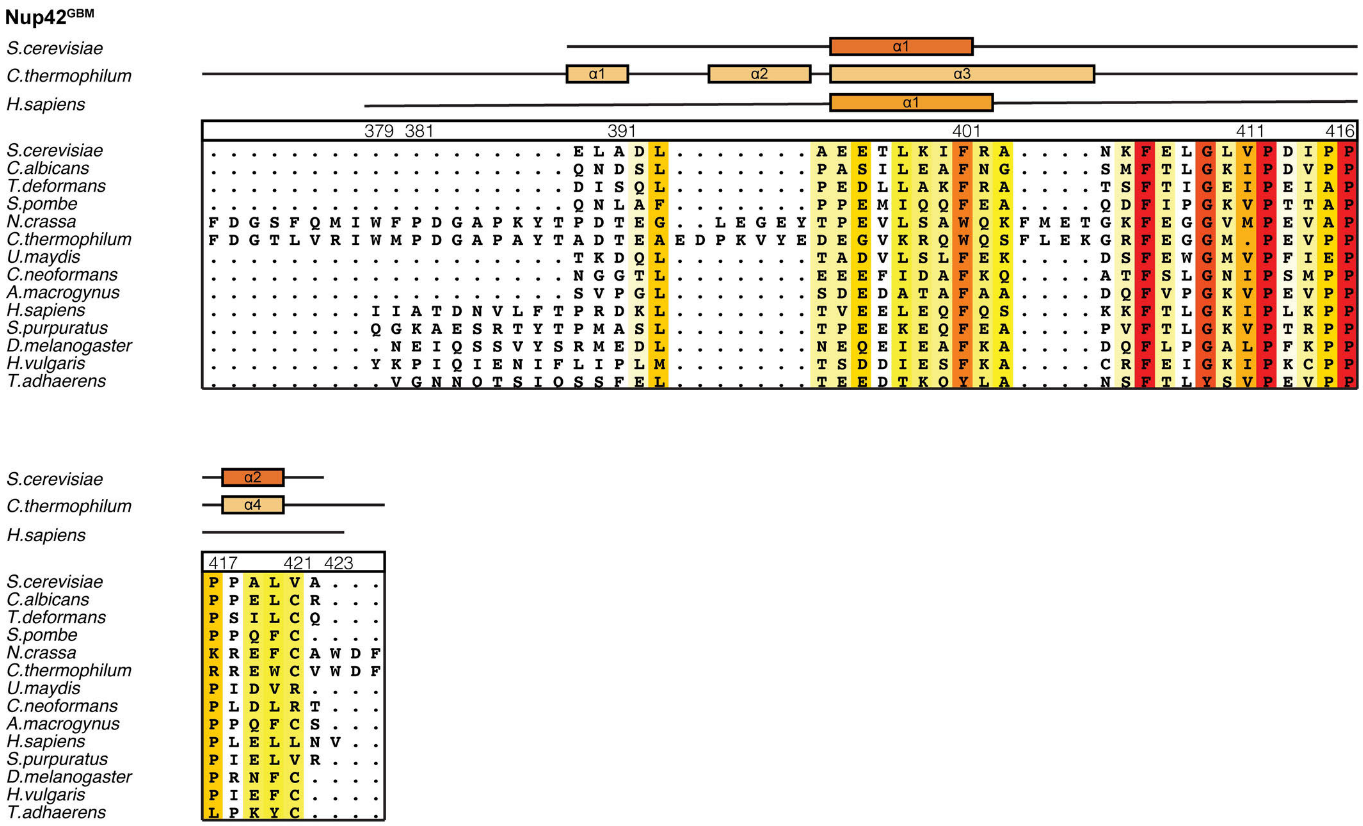

Supplementary Figure 9, Lin et al., 2018

Supplementary Figure 9: Multispecies sequence alignment of Nup42^{GBM}. Sequences from fourteen diverse species were aligned and colored by sequence similarity according to the BLOSUM62 matrix from white (less than 40 % similarity), to yellow (55 % similarity), to red (100 % identity). The numbering is according to the *H. sapiens* protein. The secondary structure is indicated above the sequences as rectangles (α-helices) and lines (unstructured regions) for the *S. cerevisiae*, *C. thermophilum*, and *H. sapiens* proteins.

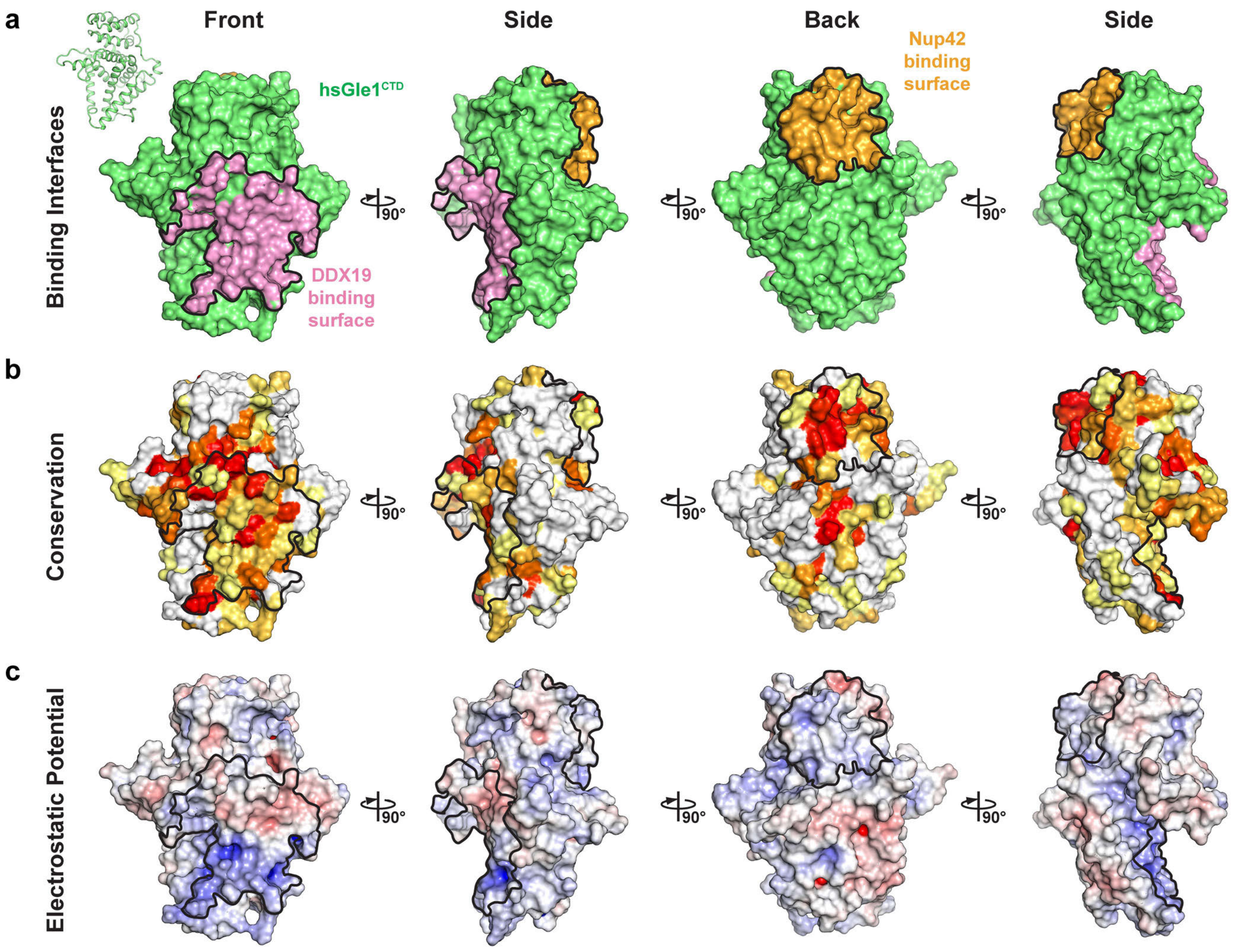

Supplementary Figure 10, Lin et al., 2018

Supplementary Figure 10: Surface properties of *H. sapiens* Gle1^{CTD}. Surface representations of Gle1^{CTD} in four orientations related by 90° rotations. The Nup42^{GBM} and DDX19 binding interfaces are outlined in black. (a) Identification of *H. sapiens* Gle1^{CTD} binding surfaces. The DDX19 and Nup42^{GBM} binding interfaces are colored pink and orange, respectively. (b) Surface representation colored according to sequence conservation for animals using an alignment containing the species *H. sapiens*, *X. tropicalis*, *D. rerio*, *S. purpuratus*, *T. castaneum*, *D. melanogaster*, *C. teleta*, *H. vulgaris*, *A. digitifera*, and *T. adhaerens*. (c) Surface representation colored according to electrostatic potential from -10 k_BT/e (red) to 0 k_BT/e (white) to +10 k_BT/e (blue).

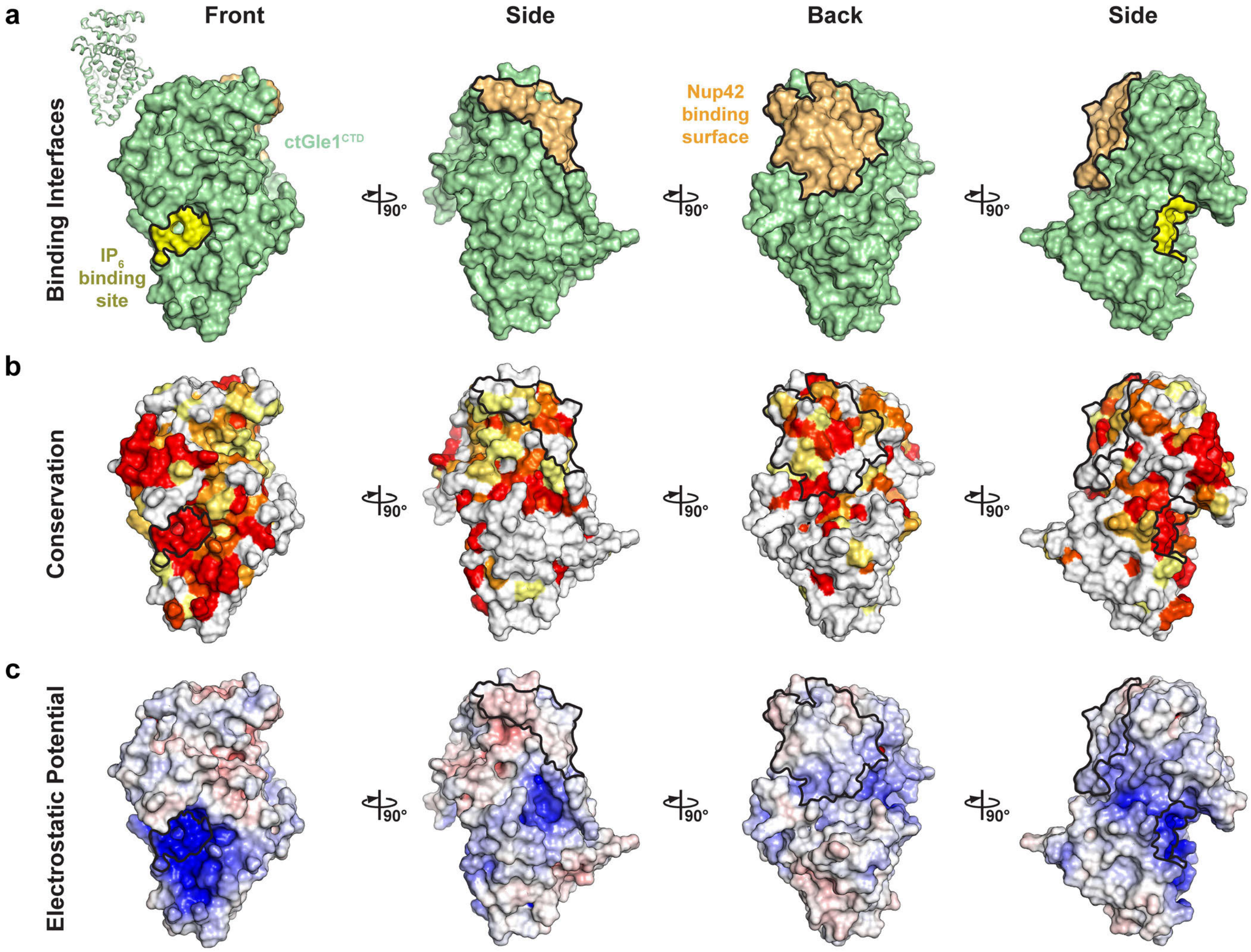

Supplementary Figure 11, Lin et al., 2018

Supplementary Figure 11: Surface properties of *C. thermophilum* Gle1^{CTD}. Surface representations of ctGle1^{CTD} in four orientations related by 90° rotations. The ctNup42^{GBM} and IP_6 binding interfaces are outlined in black. (a) Identification of ctGle1^{CTD} binding surfaces. The IP_6 and ctNup42^{GBM} binding sites are colored yellow and light orange, respectively. (b) Surface representation colored according to sequence conservation for fungi belonging to the Pezizomycotina subdivision using an alignment containing the species *C. thermophilum*, *X. heveae, S. sclerotiorum, C. militaris, P. digitatum,* and *F. verticilliodes*. (c) Surface representation colored according to electrostatic potential from -10 k_BT/e (red) to 0 k_BT/e (white) to +10 k_BT/e (blue).

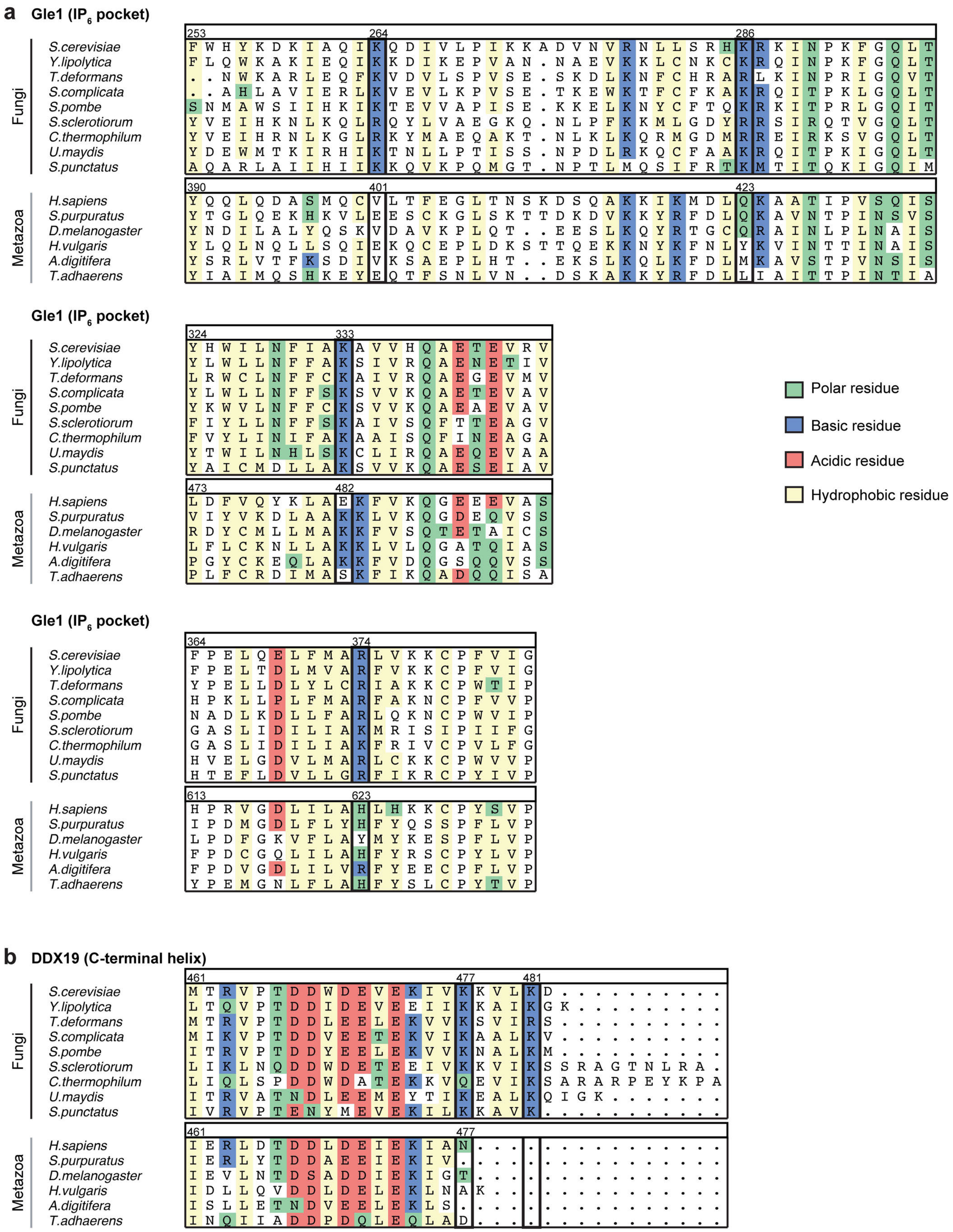

Supplementary Figure 12, Lin et al., 2018

Supplementary Figure 12: Multispecies sequence alignment of the Gle1 IP_6 pocket and the DDX19 C-terminal helix. Basic residues conserved in fungi are outlined with black boxes. Residues are colored according to an adapted Clustal color scheme.

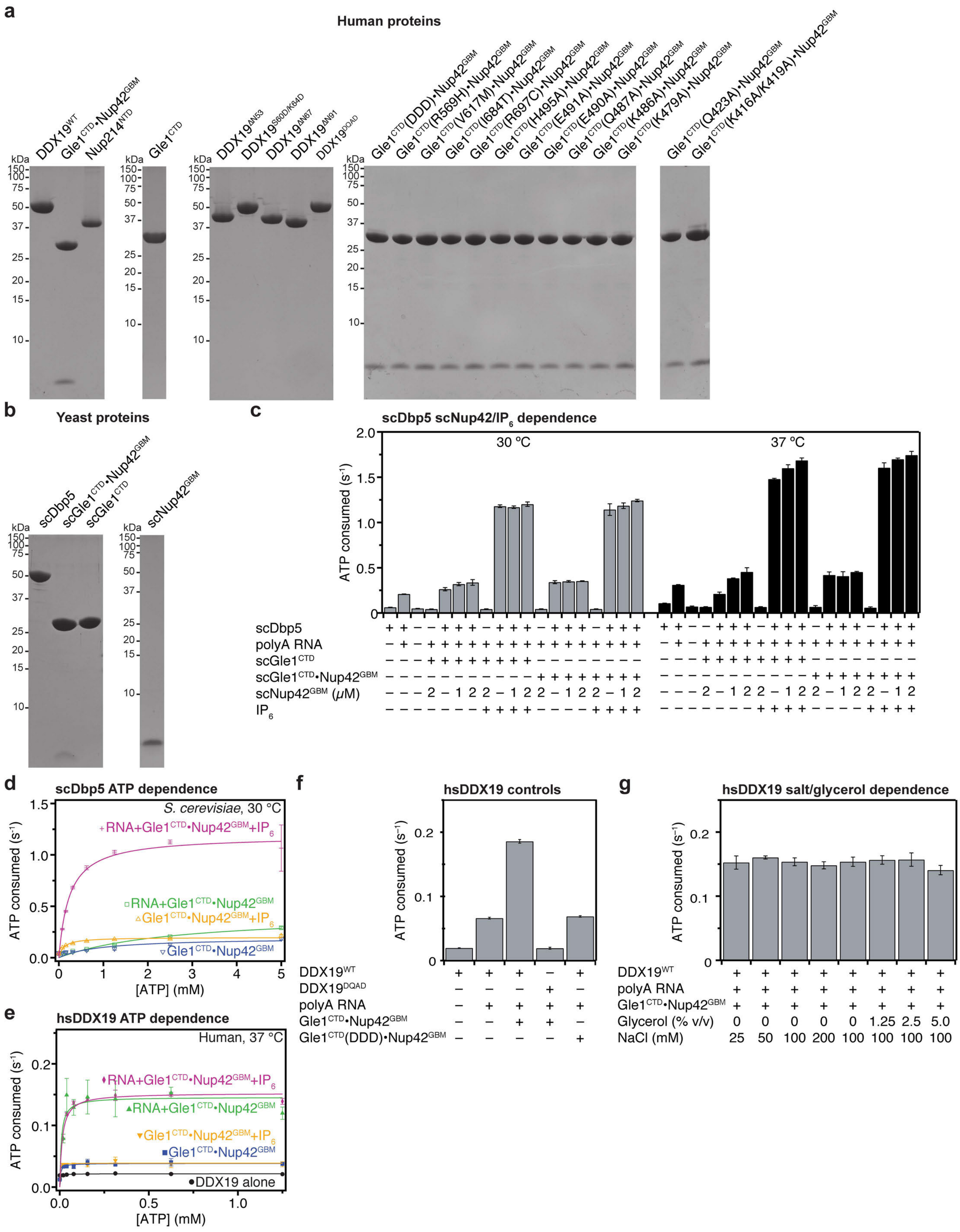

Supplementary Figure 13, Lin et al., 2018

Supplementary Figure 13: Steady state colorimetric ATPase activity assay. (a, b) SDS-PAGE analysis of the purified (a) human and (b) yeast proteins used in activity assays. (c) Steady-state ATPase activity of scDbp5 with increasing amounts of scNup42^{GBM}. Reactions were performed with 0.5 µM scDbp5, 1.0 µM scGle1^{CTD} or scGle1^{CTD}•Nup42^{GBM}, and 2.0 µM IP_6. (d) ATP dependence of scDbp5 activity. (e) ATP dependence of DDX19 activity. (f) Steady-state ATPase activity of a catalytically dead DDX19 mutant or a Gle1^{CTD} mutant that abolishes stimulation of DDX19 by Gle1^{CTD}, indicating that no activity can be attributed to contaminating factors. (f) Salt and glycerol concentration dependence of DDX19 activity. All reported values are the average of three experiments. Error bars represent standard deviation.

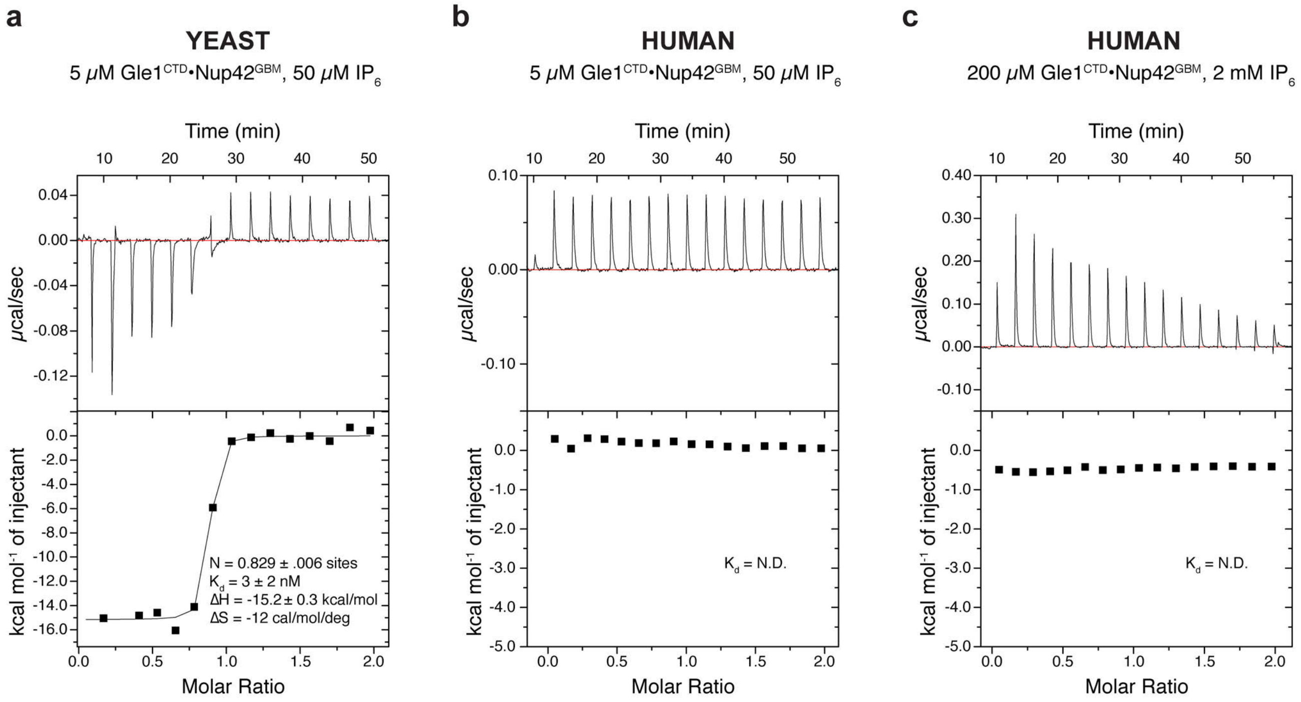

Supplementary Figure 14, Lin et al., 2018

Supplementary Figure 14: Isothermal titration calorimetry analysis of IP_6 binding. (a) Representative baseline-corrected ITC experiment for IP_6 binding to *S. cerevisiae* $Gle1^{CTD}$•$Nup42^{GBM}$ performed with 5 µM $scGle1^{CTD}$•$Nup42^{GBM}$ in the cell and 50 µM IP_6 at 21 °C. (b) Representative baseline-corrected ITC experiment IP_6 binding to human $Gle1^{CTD}$•$Nup42^{GBM}$ performed with identical conditions as (a). (c) Representative baseline-corrected ITC experiment for IP_6 binding to human $Gle1^{CTD}$•$Nup42^{GBM}$ performed with 200 µM $Gle1^{CTD}$•$Nup42^{GBM}$ in the cell and 2.0 mM IP_6 at 21 °C. All experiments were performed in triplicate. Baseline was corrected by subtracting heat release from a titration of the appropriate concentration of IP_6 into only buffer. N.D. indicates that binding could not be detected and the binding parameters were not determined.

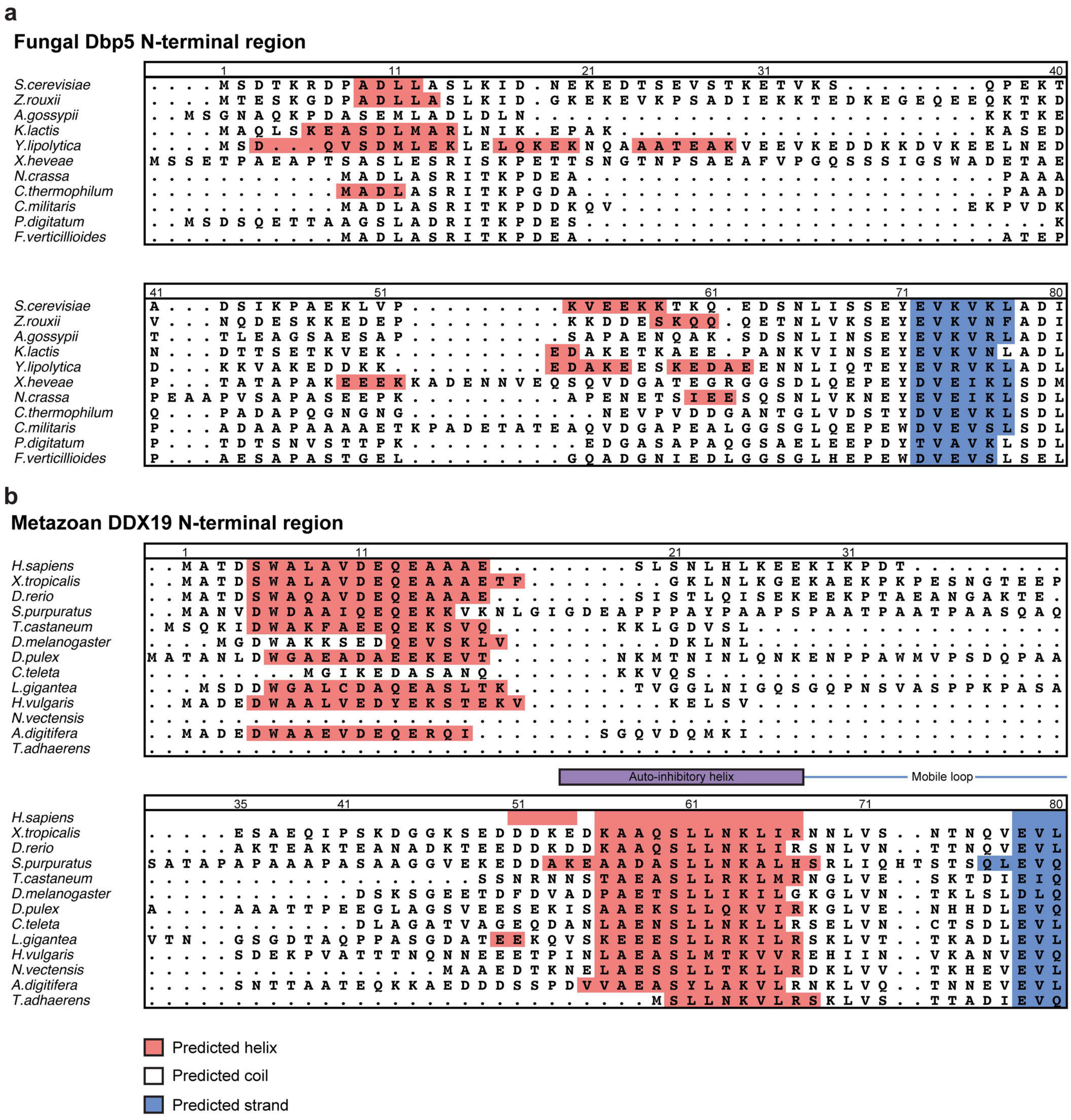

Supplementary Figure 15, Lin et al., 2018

Supplementary Figure 15: Secondary structure predictions for the N-terminal regions of fungal Dbp5 and metazoan DDX19. Sequences of the unstructured N-terminal regions of (a) fungal Dbp5 or (b) metazoan DDX19 proteins are shown and colored by predicted secondary structure from PSIPRED[6]. Predicted helical residues are colored red, predicted strand residues are colored blue, and predicted coil residues are colored white. Numbering above fungal sequences corresponds to *S. cerevisiae* residues and numbering above metazoan sequences corresponds to the human residues.

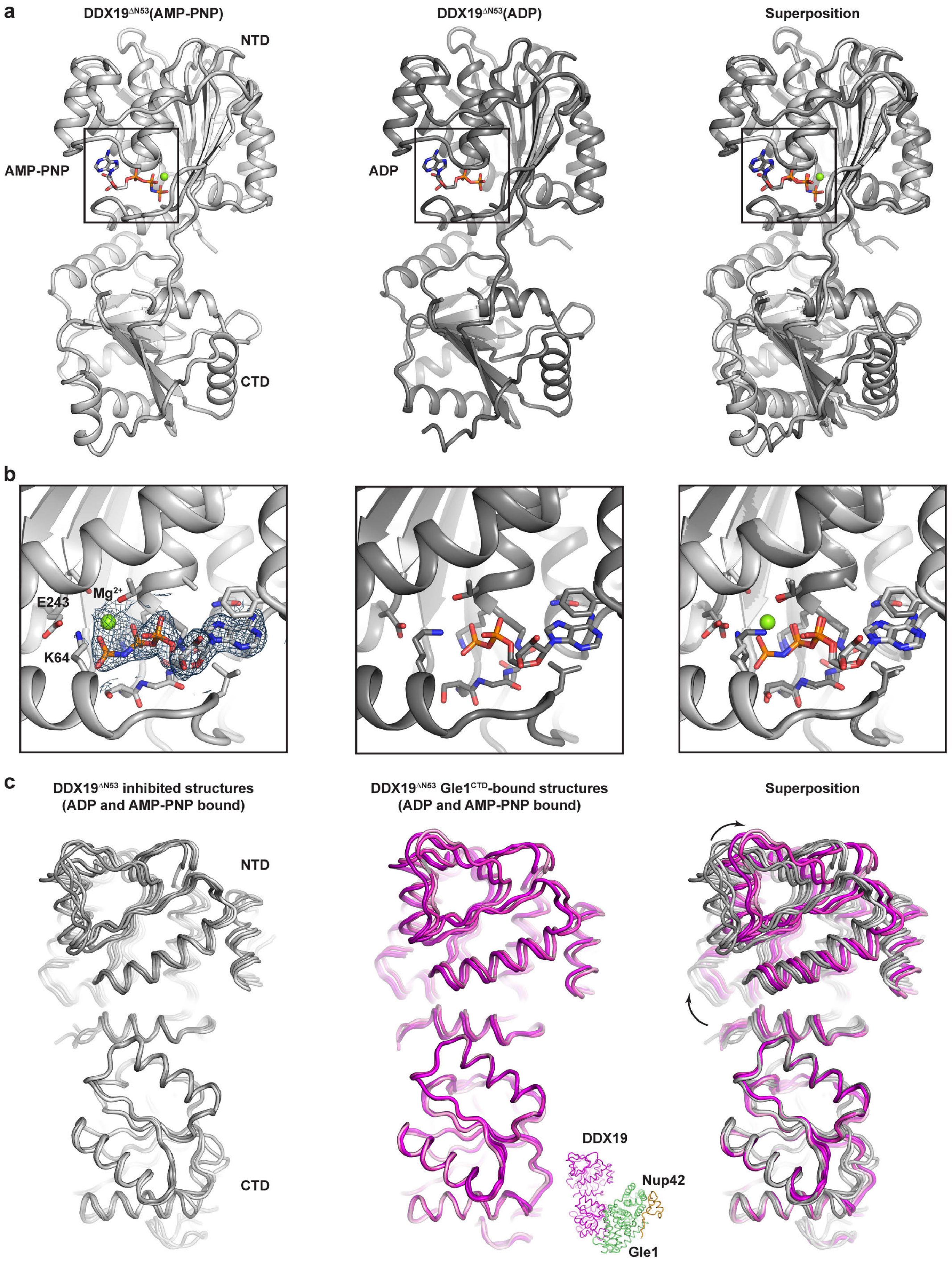

Supplementary Figure 16, Lin et al., 2018

Supplementary Figure 16: Structure of DDX19$^{\Delta N53}$(AMP-PNP•Mg^{2+}) and analysis of DDX19 conformations.

(a) Left: crystal structure of *H. sapiens* DDX19$^{\Delta N53}$(AMP-PNP•Mg^{2+}). Middle: crystal structure of *H. sapiens* DDX19$^{\Delta N53}$(ADP) (PDB ID 3EWS)[7]. Right: superposition of the two structures. (b) Zoomed view of the nucleotide binding pockets of the structures in panel (a). The $2|F_o|-|F_c|$ electron density map around the AMP-PNP molecule is shown at a contour level of 1.0 σ. (c) Left: superposition of all four molecules from the asymmetric units of the DDX19$^{\Delta N53}$(AMP-PNP•Mg^{2+}) and DDX19$^{\Delta N53}$(ADP) (PDB ID 3EWS) structures. Middle: superposition of all four molecules from the asymmetric units of the Gle1^{CTD}•Nup42^{GBM}•DDX19$^{\Delta N53}$(AMP-PNP•Mg^{2+}) and Gle1^{CTD}•Nup42^{GBM}•DDX19$^{\Delta N53}$(ADP) structures. A cartoon of the heterotrimeric complex is shown at the bottom right to indicate the orientation of the molecule shown. Right: superposition of all structures. Arrows indicate the direction of movement from the inhibited to the Gle1-bound structures.

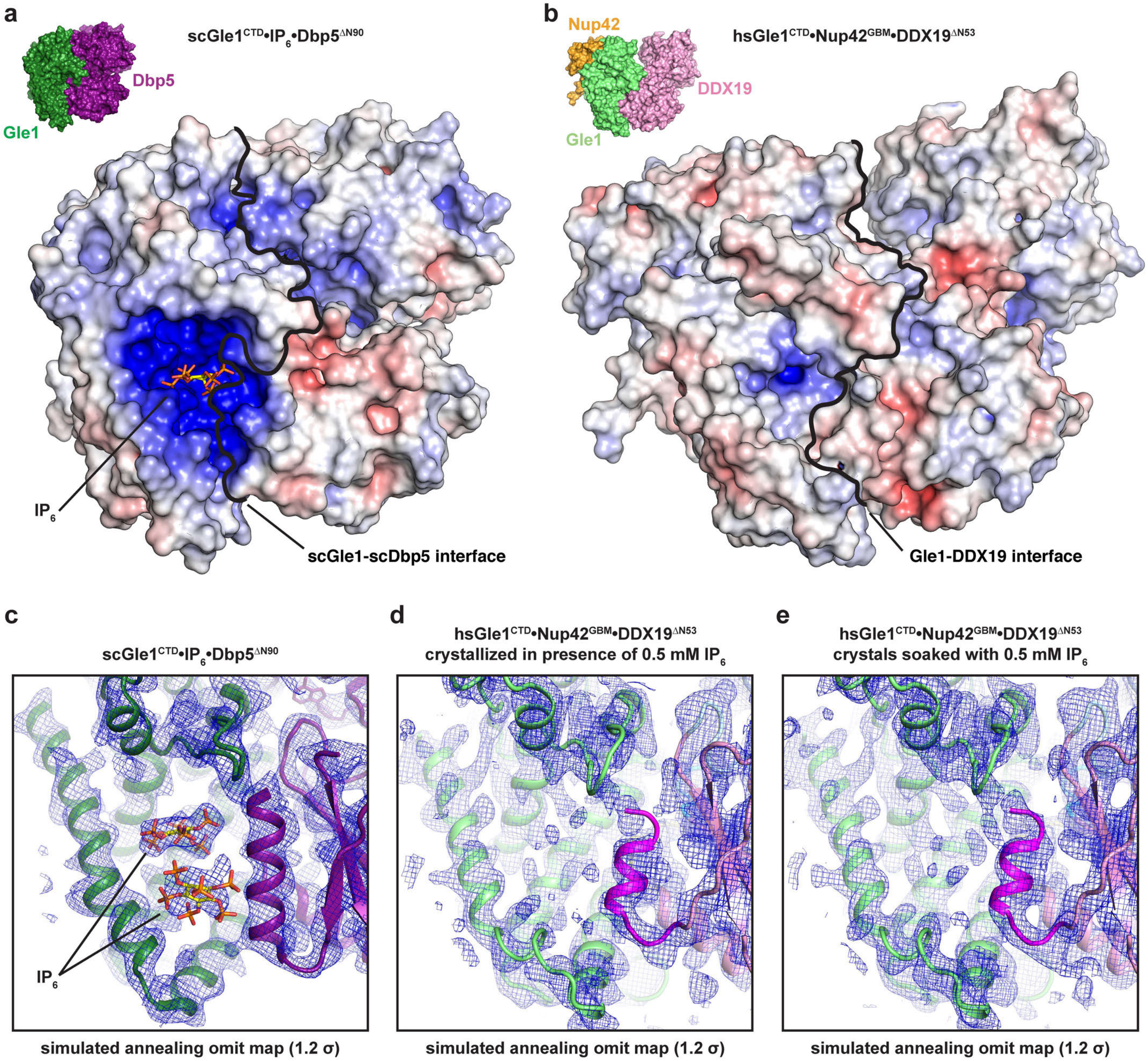

Supplementary Figure 17, Lin et al., 2018

Supplementary Figure 17: Analysis of the IP_6 binding pockets in the context of complex formation. (a) Surface view of the *S. cerevisiae* $Gle1^{CTD}$•IP_6•$Dbp5^{\Delta N90}$(ADP) complex (PDB ID 3RRN) colored according to electrostatic potential from -10 k_BT/e (red) to 0 k_BT/e (white) to +10 k_BT/e (blue)[3]. An IP_6 molecule is shown in stick representation to indicate the positively charged IP_6 binding pocket. The scGle1-scDbp5 interface is indicated by the black line. (b) Surface view of the human $Gle1^{CTD}$•$Nup42^{GBM}$•$DDX19^{\Delta N53}$(AMP-PNP•Mg^{2+}) complex colored according to electrostatic potential from -10 k_BT/e (red) to +10 k_BT/e (blue). The Gle1-Dbp5 interface is indicated by the black line. (c) A simulated annealing omit map calculated for the *S. cerevisiae* $Gle1^{CTD}$•IP_6•$Dbp5^{\Delta N90}$(ADP) complex (PDB ID 3RRN) contoured at 1.2 σ. (d) A simulated annealing omit map calculated for human $Gle1^{CTD}$•$Nup42^{GBM}$•$DDX19^{\Delta N53}$(AMP-PNP•Mg^{2+}) grown in the presence of 0.5 mM IP_6 contoured at 1.2 σ. (e) A simulated annealing omit map calculated for human $Gle1^{CTD}$•$Nup42^{GBM}$•$DDX19^{\Delta N53}$(AMP-PNP•Mg^{2+}) soaked with 0.5 mM IP_6 contoured at 1.2 σ.

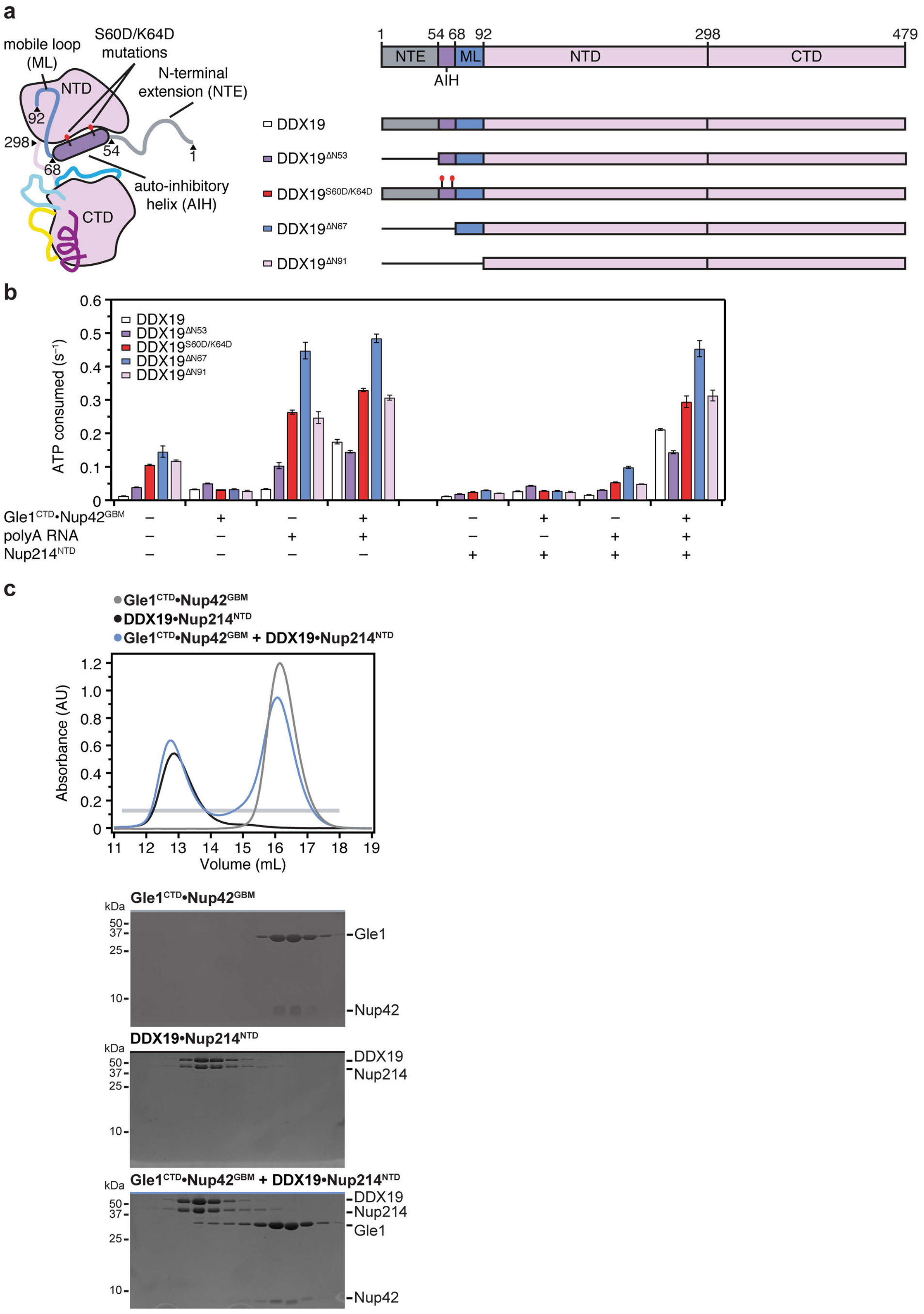

Supplementary Figure 18, Lin et al., 2018

Supplementary Figure 18: Gle1^{CTD}•Nup42^{GBM} rescues DDX19 inhibition by Nup214^{NTD} in the presence and absence of the auto-inhibitory helix. (a) Schematics of the construct boundaries of DDX19 variants used in steady-state ATPase assays. (b) Steady-state ATPase rates of DDX19 variants in the presence and absence of Nup214^{NTD}. Values reported are the average of three experiments. Error bars represent standard deviation. (c) SEC analysis of the interaction between Gle1^{CTD}•Nup42^{GBM} and DDX19•Nup214^{NTD}. 40 µM of purified Gle1^{CTD}•Nup42^{GBM} complex was mixed with 20 µM purified DDX19•Nup214^{NTD} and loaded onto a Superdex 200 10/300 GL column. The gray bar indicates the fractions visualized with Coomassie-stained SDS-PAGE gels.

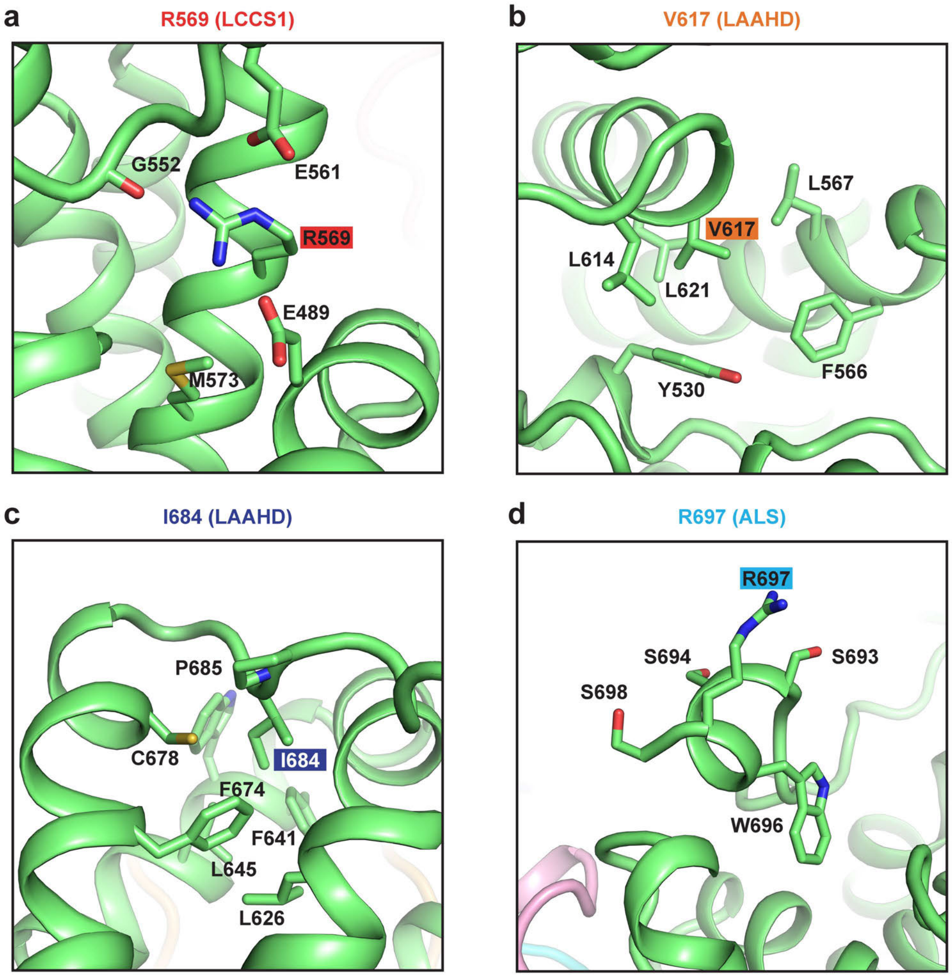

Supplementary Figure 19, Lin et al., 2018

Supplementary Figure 19: Impact of Gle1 disease-related mutations. (a-d) Zoomed views of the Gle1 residues mutated in human disease.

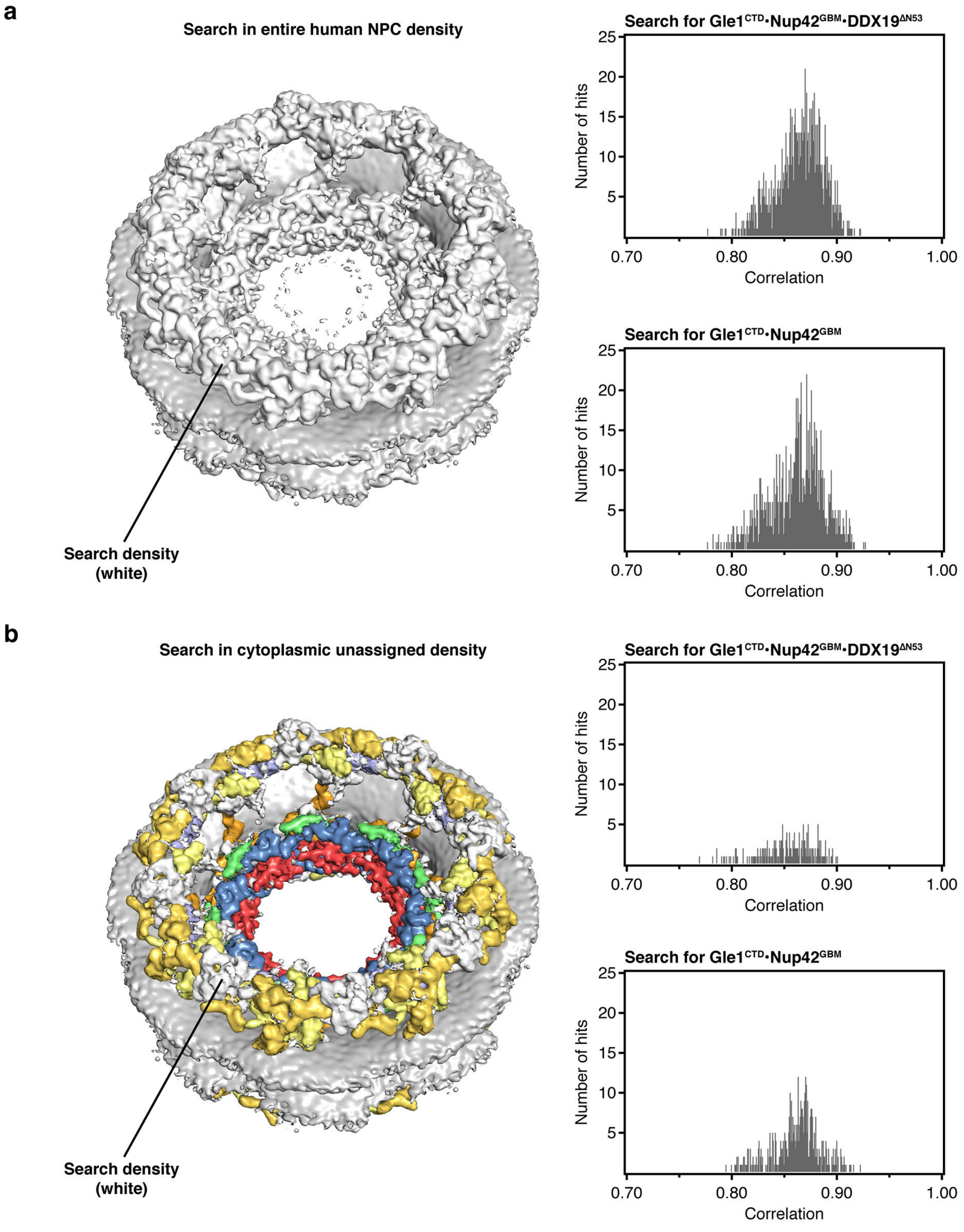

Supplementary Figure 20, Lin et al., 2018

Supplementary Figure 20: Searches for human crystal structures in the cryoelectron tomographic reconstruction of the intact human NPC. (a) Left: the cryoelectron tomographic reconstruction of the human NPC is shown on the left, with the protein density colored white and nuclear envelope colored in gray. Searches were performed with 50,000 random positions within 100 Å of the cytoplasmic outer ring. Histograms on the right show the distribution of scores. (b) Right: the cryoelectron tomographic reconstruction of the human NPC is colored according to the assigned density in the composite structure of the NPC[8]. Searches were performed with 50,000 random positions within 100 Å of the cytoplasmic outer ring into the remaining cytoplasmic density, colored in white. Histograms on the right show the distribution of scores.

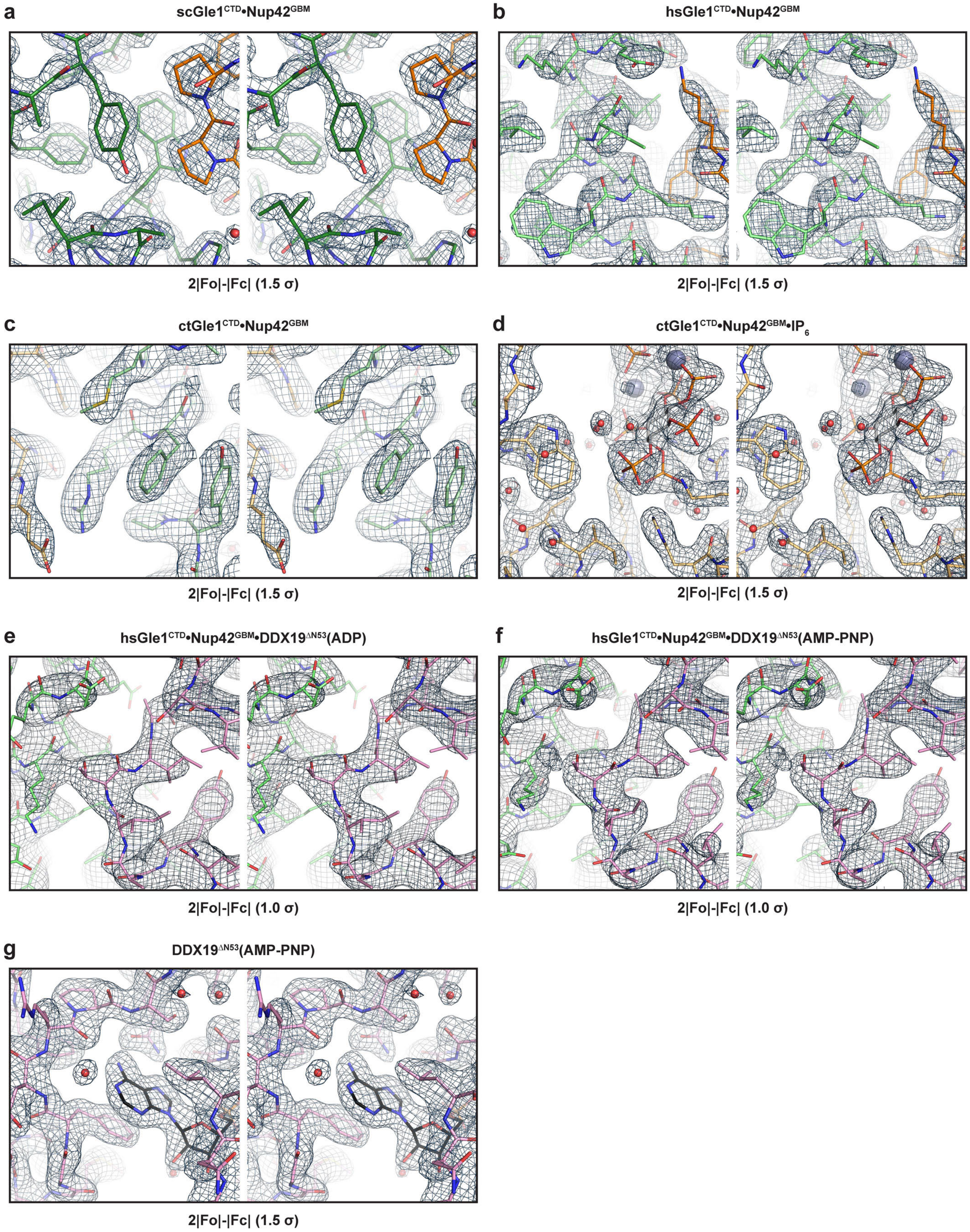

Supplementary Figure 21, Lin et al., 2018

Supplementary Figure 21: Representative stereo views of electron densities for reported structures. (a-g) Cross-eyed stereo views for each of the reported structures. All maps are $2|F_o|-|F_c|$ maps contoured at the threshold values indicated below each stereo view.

Supplementary Table 1.

Data collection and refinement statistics for *S. cerevisiae* and *H. sapiens* Gle1^{CTD}•Nup42^{GBM}structures

	scGle1^{CTD}•Nup42^{GBM}	hsGle1^{CTD}•Nup42^{GBM}
**Data collection**		
Space group	$P4_32_12$	C2
Cell dimensions		
a, *b*, *c* (Å)	64.5, 64.5, 361.7	163.7, 69.2, 93.0
α, β, γ (°)	90.0 90.0 90.0	90.0, 90.6, 90.0
Resolution (Å) [a,b]	50.0–1.75 (1.81–1.75)	50.0–2.8 (2.9–2.8)
R_{meas} [b]	12.4 (127.0)	10.7 (144.3)
$I / \sigma I$ [b]	16.4 (1.3)	10.1 (1.1)
Completeness (%) [b]	99.3 (93.5)	99.2 (97.8)
Redundancy [b]	21.8 (10.5)	3.8 (3.5)
**Refinement**		
Resolution (Å)	47.3–1.75	46.8–2.8
No. reflections	85,335	20,826[c]
R_{work} / R_{free}	18.5/21.1	24.5/27.4
No. atoms	7,314	5,835
Protein	6,674	5,794
Ligand/ion	8	12
Water	632	29
B-factors	39	59
Protein	39	59
Ligand/ion	43	63
Water	43	31
R.m.s. deviations		
Bond lengths (Å)	0.007	0.003
Bond angles (°)	1.1	0.6

[a]Diffraction data were obtained from a single crystal
[b]Values in parentheses are for highest-resolution shell.
[c]Refinement was performed with ellipsoidally truncated data

Supplementary Table 2.

Data collection, phasing and refinement statistics for *C. thermophilum* $Gle1^{CTD}$•$Nup42^{GBM}$ structures

	$ctGle1^{CTD}$•$Nup42^{GBM}$	$ctGle1^{CTD}$•$Nup42^{GBM}$•IP_6 Native	$ctGle1^{CTD}$•$Nup42^{GBM}$•IP_6 SeMet
**Data collection**			
Space group	$P2_12_12_1$	C2	C2
Cell dimensions			
a, *b*, *c* (Å)	84.5, 93.1, 229.7	119.2 73.3 117.7	118.7, 72.8, 117.7
α, β, γ (°)	90.0, 90.0, 90.0	90.0, 94.2, 90.	090.0, 95.5, 90.0
			Peak
Wavelength	1.0332	1.0332	0.9792
Resolution (Å) [a,b]	50.0-2.65 (2.73-2.65)	35.0-2.17 (2.25-2.17)	50.0-3.2 (3.31-3.20)
R_{meas} [b]	9.3 (148.7)	11.1 (131.6)	17.9 (86.0)
$I / \sigma I$ [b]	20.5 (1.4)	11.1 (1.9)	10.0 (3.2)
Completeness (%) [b]	99.8 (97.6)	98.4 (98.5)	98.6 (99.0)
Redundancy [b]	13.1 (10.5)	6.7 (6.9)	13.6 (14.0)
**Refinement**			
Resolution (Å)	48.4–2.65	35.0–2.17	
No. reflections	53,433	52,810	
R_{work} / R_{free}	24.0/27.7	19.3/23.0	
No. atoms	11,561	6,657	
Protein	11,418	6,060	
Ligand/ion	60	244	
Water	83	353	
B-factors	89	52	
Protein	89	51	
Ligand/ion	96	73	
Water	57	52	
R.m.s deviations			
Bond lengths (Å)	0.005	0.003	
Bond angles (°)	1.0	0.5	

[a]Diffraction data were obtained from a single crystal
[b]Values in parentheses are for highest-resolution shell.
[c]Refinement was performed with ellipsoidally truncated data

Supplementary Table 3.

Data collection and refinement statistics for DDX19$^{\Delta N53}$ and complexes

	hsGle1^{CTD}•Nup42^{GBM}• DDX19$^{\Delta N53}$•ADP	hsGle1^{CTD}•Nup42^{GBM}• DDX19$^{\Delta N53}$•AMP-PNP	DDX19$^{\Delta N53}$•AMP-PNP
**Data collection**			
Space group	$P2_1$	$P2_1$	$P2_1$
Cell dimensions			
a, *b*, *c* (Å)	87.7, 74.7, 146.8	87.6, 73.4, 145.3	83.4, 45.6, 127.6
α, β, γ (°)	90.0, 94.8, 90.0	90.0, 95.1, 90.0	90.0, 97.0, 90.0
Resolution (Å) [a,b]	50.0–3.6 (3.9-3.6)	50.0–3.4 (3.5–3.4)	50.0–2.2 (2.3-2.2)
R_{meas} [b]	15.9 (223.2)	29.8 (406.9)	14.0 (245.6)
$I / \sigma I$ [b]	8.5 (1.0)	6.6 (0.9)	10.0 (1.7)
Completeness (%) [b]	98.6 (98.9)	100.0 (100.0)	99.4 (97.9)
Redundancy [b]	6.9 (7.0)	10.2 (10.4)	6.9 (6.1)
**Refinement**			
Resolution (Å)	44.3–3.6	45.8–3.4	41.3–2.2
No. reflections	18,969[c]	21,298[c]	48,299
R_{work} / R_{free}	23.2/28.1	26.1/31.3	20.7/24.8
No. atoms	12,609	12,598	7,026
Protein	12,523	12,504	6,722
Ligand/ion	86	94	85
Water	-	-	219
B-factors	139	107	77
Protein	139	107	78
Ligand/ion	134	99	53
Water	-	-	51
R.m.s. deviations			
Bond lengths (Å)	0.003	0.003	0.004
Bond angles (°)	0.9	1.0	0.6

[a]Diffraction data were obtained from a single crystal
[b]Values in parentheses are for highest-resolution shell.
[c]Refinement was performed with ellipsoidally truncated data

Supplementary Table 4.

Data collection and refinement statistics for hsGle1^{CTD}•Nup42^{CTD}•DDX19$^{\Delta N53}$•AMP-PNP with IP_6

	hsGle1^{CTD}•Nup42^{GBM}• DDX19$^{\Delta N53}$•AMP-PNP soaked with IP_6	hsGle1^{CTD}•Nup42^{GBM}• DDX19$^{\Delta N53}$•AMP-PNP co-crystallized with IP_6
**Data collection**		
Space group	$P2_1$	$P2_1$
Cell dimensions		
a, *b*, *c* (Å)	87.8, 74.0, 147.1	87.8, 73.5, 147.0
α, β, γ (°)	90.0, 94.9, 90.0	90.0, 95.0, 90.0
Resolution (Å) [a,b]	50.0–3.6 (3.7–3.6)	50.0–3.4 (3.5–3.4)
R_{meas} [b]	15.9 (223.2)	29.8 (406.9)
$I / \sigma I$ [b]	8.5 (1.0)	6.6 (0.9)
Completeness (%) [b]	98.6 (98.9)	100.0 (100.0)
Redundancy [b]	6.9 (7.0)	10.2 (10.4)
**Refinement**		
Resolution (Å)	38.0–3.7	38.0–3.4
No. reflections	16,932[c]	16,848[c]
R_{work} / R_{free}	24.6/29.7	25.8/31.3
No. atoms	12,598	12,598
Protein	12,504	12,504
Ligand/ion	94	94
Water	-	-
B-factors	128	119
Protein	128	119
Ligand/ion	123	116
Water	-	-
R.m.s. deviations		
Bond lengths (Å)	0.003	0.003
Bond angles (°)	1.0	1.0

[a]Diffraction data were obtained from a single crystal
[b]Values in parentheses are for highest-resolution shell.
[c]Refinement was performed with ellipsoidally truncated data

Supplementary Table 5.

Bacterial expression constructs and expression conditions

#	Protein	Residues	Expression vector	Restriction sites 5’, 3’	N-terminal overhang	C-terminal overhang	Expression conditions
1	hsNup155 CTD	870-1391	pET28a-PreS	NdeI, NotI	GPHM	-	18 °C / 18 hours
2	hsGle1 N	2-33	pET28a-SUMO	BamHI, NotI	Smt3p-S	-	30 °C / 2 hours
3	hsNup98 ΔFG	498-880	pET28a-SUMO	BamHI, NotI	S	-	18 °C / 18 hours
4	hsNup155 CTD E1146A	870-1391	pET28a-PreS	NdeI, NotI	GPHM	-	18 °C / 18 hours
5	hsNup155 CTD K1147A	870-1391	pET28a-PreS	NdeI, NotI	GPHM	-	18 °C / 18 hours
6	hsNup155 CTD L1182A	870-1391	pET28a-PreS	NdeI, NotI	GPHM	-	18 °C / 18 hours
7	hsNup155 CTD Y1189A	870-1391	pET28a-PreS	NdeI, NotI	GPHM	-	18 °C / 18 hours
8	hsNup155 CTD F1192A	870-1391	pET28a-PreS	NdeI, NotI	GPHM	-	18 °C / 18 hours
9	hsNup155 CTD I1206A	870-1391	pET28a-PreS	NdeI, NotI	GPHM	-	18 °C / 18 hours
10	hsGle1 N L11A	2-33	pET28a-SUMO	BamHI, NotI	Smt3p-S	-	30 °C / 2 hours
11	hsGle1 N K19A	2-33	pET28a-SUMO	BamHI, NotI	Smt3p-S	-	30 °C / 2 hours
12	hsGle1 N L22A	2-33	pET28a-SUMO	BamHI, NotI	Smt3p-S	-	30 °C / 2 hours
13	hsGle1 N C23A	2-33	pET28a-SUMO	BamHI, NotI	Smt3p-S	-	30 °C / 2 hours
14	hsGle1 N Y24A	2-33	pET28a-SUMO	BamHI, NotI	Smt3p-S	-	30 °C / 2 hours
15	hsGle1 N R26A	2-33	pET28a-SUMO	BamHI, NotI	Smt3p-S	-	30 °C / 2 hours
16	hsGle1 N W28A	2-33	pET28a-SUMO	BamHI, NotI	Smt3p-S	-	30 °C / 2 hours
17	scGle1 CTD	244-538	pET28a-PreS	NdeI, XhoI	GPHM	-	18 °C / 18 hours
18♦	scGle1 CTD<br>scNup42 GBM	244-538<br>397-430	pETDuet PreS	NdeI, XhoI<br>BamHI, NotI	M<br>GPSGS	-<br>-	18 °C / 18 hours
19	hsGle1 CTD	382-698	pET28a-SUMO	BamHI, NotI	S	-	18 °C / 18 hours
20♦	hsGle1 CTD<br>hsNup42 GBM	382-698<br>379-423	pETDuet PreS	NdeI, XhoI<br>BamHI, NotI	M<br>GPSGS	-<br>-	18 °C / 18 hours
21♦	ctGle1 CTD	216-519	pET28a-PreS	NdeI, NotI	GPHM	-	37C °C / 3 hours co-expressed with ctNup42
22♦	ctNup42 GBM	494-558	pGex6P-1 PreS	EcoRI, XhoI	GPHMGSP EF	-	37C °C / 3 hours co-expressed with ctGle1
23	scNup42 GBM	397-430	pET28a-SUMO	BamHI, XhoI	S	YALEHHHHHH	37 °C / 2 hours
24	scNup42 GBM F409A	397-430	pET28a-SUMO	BamHI, XhoI	S	YALEHHHHHH	37 °C / 2 hours
25	scNup42 GBM F414A	397-430	pET28a-SUMO	BamHI, XhoI	S	YALEHHHHHH	37 °C / 2 hours
26	scNup42 GBM L416A	397-430	pET28a-SUMO	BamHI, XhoI	S	YALEHHHHHH	37 °C / 2 hours
27	scNup42 GBM L416R	397-430	pET28a-SUMO	BamHI, XhoI	S	YALEHHHHHH	37 °C / 2 hours
28	scNup42 GBM P423A	397-430	pET28a-SUMO	BamHI, XhoI	S	YALEHHHHHH	37 °C / 2 hours
29	scNup42 GBM F409D	397-430	pET28a-SUMO	BamHI, XhoI	S	YALEHHHHHH	37 °C / 2 hours
30	scNup42 GBM F414D	397-430	pET28a-SUMO	BamHI, XhoI	S	YALEHHHHHH	37 °C / 2 hours
31	scNup42 GBM F409D/F414D	397-430	pET28a-SUMO	BamHI, XhoI	S	YALEHHHHHH	37 °C / 2 hours
32	scDbp5	1-482	pET28a-PreS	NdeI, NotI	GPHM	-	18 °C / 18 hours
33♦	hsDDX19	1-479	pET28a-PreS	NdeI, NotI	GPH	-	18 °C / 18 hours
34	ctDbp5	1-477	pETMCN-SUMO	BamHI, NotI	S	-	18 °C / 18 hours
35♦	hsDDX19 ΔN53	54-479	pET28a-PreS	NdeI, NotI	GPHM	-	18 °C / 18 hours
36	hsGle1 CTD H495A<br>hsNup42 GBM	382-698<br>379-423	pETDuet PreS	NdeI, XhoI<br>BamHI, NotI	M<br>GPSGS	-<br>-	18 °C / 18 hours
37	hsGle1 CTD E491A<br>hsNup42 GBM	382-698<br>379-423	pETDuet PreS	NdeI, XhoI<br>BamHI, NotI	M<br>GPSGS	-<br>-	18 °C / 18 hours
38	hsGle1 CTD E490A<br>hsNup42 GBM	382-698<br>379-423	pETDuet PreS	NdeI, XhoI<br>BamHI, NotI	M<br>GPSGS	-<br>-	18 °C / 18 hours
39	hsGle1 CTD Q487A<br>hsNup42 GBM	382-698<br>379-423	pETDuet PreS	NdeI, XhoI<br>BamHI, NotI	M<br>GPSGS	-<br>-	18 °C / 18 hours
40	hsGle1 CTD K486A<br>hsNup42 GBM	382-698<br>379-423	pETDuet PreS	NdeI, XhoI<br>BamHI, NotI	M<br>GPSGS	-<br>-	18 °C / 18 hours
41	hsGle1 CTD K479A<br>hsNup42 GBM	382-698<br>379-423	pETDuet PreS	NdeI, XhoI<br>BamHI, NotI	M<br>GPSGS	-<br>-	18 °C / 18 hours
42	hsGle1 CTD Q423A<br>hsNup42 GBM	382-698<br>379-423	pETDuet PreS	NdeI, XhoI<br>BamHI, NotI	M<br>GPSGS	-<br>-	18 °C / 18 hours
43	hsGle1 CTD K416A/K419A<br>hsNup42 GBM	382-698<br>379-423	pETDuet PreS	NdeI, XhoI<br>BamHI, NotI	M<br>GPSGS	-<br>-	18 °C / 18 hours
44	hsDDX19 ΔN67	68-479	pET28a-PreS	NdeI, NotI	GPHM	-	18 °C / 18 hours
45	hsDDX19 ΔN91	92-479	pET28a-PreS	NdeI, NotI	GPHM	-	18 °C / 18 hours
46	hsDDX19 S60D/K64D	1-479	pET28a-PreS	NdeI, NotI	GPH	-	18 °C / 18 hours
47	Nup214 NTD	1-405	pET28a-PreS	NdeI, NotI	GPH	-	18 °C / 18 hours

#	Protein	Residues	Expression vector	Restriction sites 5’, 3’	N-terminal overhang	C-terminal overhang	Expression conditions
48	hsGle1 CTD G666D/I669D/Q673D<br>hsNup42 GBM	382-698<br>379-423	pETDuet PreS	NdeI, XhoI<br>BamHI, NotI	M<br>GPSGS	-<br>-	18 °C / 18 hours
49	hsGle1 CTD R569H<br>hsNup42 GBM	382-698<br>379-423	pETDuet PreS	NdeI, XhoI<br>BamHI, NotI	M<br>GPSGS	-<br>-	18 °C / 18 hours
50	hsGle1 CTD V617M<br>hsNup42 GBM	382-698<br>379-423	pETDuet PreS	NdeI, XhoI<br>BamHI, NotI	M<br>GPSGS	-<br>-	18 °C / 18 hours
51	hsGle1 CTD I684T<br>hsNup42 GBM	382-698<br>379-423	pETDuet PreS	NdeI, XhoI<br>BamHI, NotI	M<br>GPSGS	-<br>-	18 °C / 18 hours
52	hsGle1 CTD R697C<br>hsNup42 GBM	382-698<br>379-423	pETDuet PreS	NdeI, XhoI<br>BamHI, NotI	M<br>GPSGS	-<br>-	18 °C / 18 hours
53	hsDDX19 E243Q (DQAD)	1-479	pET28a-PreS	NdeI, NotI	GPH	-	18 °C / 18 hours
54	hsGle1 N Y25A	2-33	pET28a-SUMO	BamHI, NotI	Smt3p-S	-	30 °C / 2 hours
55	hsNup155 CTD	870-1391	pGex6P-1 PreS	NdeI, NotI	GST-LEVLFQGPHM	-	18 °C / 18 hours

♦ Constructs that were used for crystallization

Supplementary Table 6.

Yeast constructs

Plasmid	Protein	Residues (Mutations)	Vector	Restriction Sites 5', 3'	Selection
pRS415-P_{Nop1}-mCherry-3xHA	N/A	N/A	pRS415	N/A	LEU2
pRS415-P_{Nop1}-NUP42-mCherry-3xHA	Nup42	1-430	pRS415	NdeI, SpeI	LEU2
pRS415-P_{Nop1}-nup42 (364-430)-mCherry-3xHA	Nup42	364-430	pRS415	NdeI, SpeI	LEU2
pRS415-P_{Nop1}-nup42 (397-430)-mCherry-3xHA	Nup42	397-430	pRS415	NdeI, SpeI	LEU2
pRS415-P_{Nop1}-nup42 (410-430)-mCherry-3xHA	Nup42	410-430	pRS415	NdeI, SpeI	LEU2
pRS415-P_{Nop1}-nup42 (1-394)-mCherry-3xHA	Nup42	1-394	pRS415	NdeI, SpeI	LEU2
pRS415-P_{Nop1}-nup42 F409D-mCherry-3xHA	Nup42	1-430 (F409D)	pRS415	NdeI, SpeI	LEU2
pRS415-P_{Nop1}-nup42 F414D-mCherry-3xHA	Nup42	1-430 (F414D)	pRS415	NdeI, SpeI	LEU2
pRS415-P_{Nop1}-nup42 F409D/F414D-mCherry-3xHA	Nup42	1-430 (F409D/F414D)	pRS415	NdeI, SpeI	LEU2

Supplementary References

1. Rollenhagen, C., Hodge, C. A. & Cole, C. N. The nuclear pore complex and the DEAD box protein Rat8p/Dbp5p have nonessential features which appear to facilitate mRNA export following heat shock. *Mol Cell Biol* **24**, 4869-4879, (2004).
2. Stutz, F., Neville, M. & Rosbash, M. Identification of a novel nuclear pore-associated protein as a functional target of the HIV-1 Rev protein in yeast. *Cell* **82**, 495-506, (1995).
3. Montpetit, B. *et al.* A conserved mechanism of DEAD-box ATPase activation by nucleoporins and InsP6 in mRNA export. *Nature* **472**, 238-242, (2011).
4. Napetschnig, J. *et al.* Structural and functional analysis of the interaction between the nucleoporin Nup214 and the DEAD-box helicase Ddx19. *Proc Natl Acad Sci U S A* **106**, 3089-3094, (2009).
5. von Moeller, H., Basquin, C. & Conti, E. The mRNA export protein DBP5 binds RNA and the cytoplasmic nucleoporin NUP214 in a mutually exclusive manner. *Nat Struct Mol Biol* **16**, 247-254, (2009).
6. Buchan, D. W., Minneci, F., Nugent, T. C., Bryson, K. & Jones, D. T. Scalable web services for the PSIPRED Protein Analysis Workbench. *Nucleic Acids Res* **41**, W349-357, (2013).
7. Collins, R. *et al.* The DEXD/H-box RNA helicase DDX19 is regulated by an {alpha}-helical switch. *J Biol Chem* **284**, 10296-10300, (2009).
8. Lin, D. H. *et al.* Architecture of the symmetric core of the nuclear pore. *Science* **352**, aaf1015, (2016).
