# Supplementary material for: Structural and functional analysis of mRNA export regulation by the nuclear pore complex
Source: Nat Commun. 2018 Jun 13;9:2319. doi: 10.1038/s41467-018-04459-3 (PMC5998080; doi:10.1038/s41467-018-04459-3)
Supplement: Supplementary file 2 — Description of Additional Supplementary Files [file 41467_2018_4459_MOESM2_ESM.pdf]

Description of Additional Supplementary Files

File Name: Supplementary Movie 1

Description: An evolutionarily conserved mechanism of interaction between Gle1 and Nup42. Rotating views of the crystal structures of (left) S. cerevisiae, (middle) H. sapiens, and (right) C. thermophilum Gle1CTD•Nup42GBM complexes reveal highly conserved protein folds and interaction interfaces.

File Name: Supplementary Movie 2

Description: Conformational changes in DDX19 induced by Gle1CTD binding in the human system. A morph animation demonstrating the range of conformational changes in DDX19 observed before and after Gle1 binding. The starting state is derived from the crystal structure of DDX19ΔN53(ADP) (PDB ID 3EWS). The final state is derived from the crystal structure of Gle1CTD•Nup42GBM•DDX19ΔN53(ADP).

File Name: Supplementary Movie 3

Description: Proposed series of conformational changes in DDX19 induced by Gle1CTD-binding to facilitate RNA-loading in the human system. A morph movie cycling from the 2 DDX19 inhibited state to the late and early Gle1-bound states to the RNA-bound state. The starting state is derived from the crystal structure of DDX19ΔN53(ADP) (PDB ID 3EWS). The early Gle1-bound state is derived from the crystal structure of Gle1CTD•Nup42GBM•DDX19ΔN53(ADP). The late Gle1-bound state was derived by superposing the human structure onto the structure of Gle1CTD•IP6•Dbp5ΔN90(ADP) (PDB ID 3RRN)10. The RNA-bound state is derived from the crystal structure of DDX19ΔN53(AMP-PNP•Mg2+)•U10 RNA (PDB ID 3G0H).
